# Oncogenic dependency on β-catenin in liver cancer cell lines correlates with pathway activation

**DOI:** 10.18632/oncotarget.21298

**Published:** 2017-09-28

**Authors:** Zhihu Ding, Chaomei Shi, Lan Jiang, Tatiana Tolstykh, Hui Cao, Dinesh S. Bangari, Susan Ryan, Mikhail Levit, Taiguang Jin, Karl Mamaat, Qunyan Yu, Hui Qu, Joern Hopke, May Cindhuchao, Dietmar Hoffmann, Fangxian Sun, Mike W. Helms, Kerstin Jahn-Hofmann, Sabine Scheidler, Liang Schweizer, Douglas D. Fang, Jack Pollard, Christopher Winter, Dmitri Wiederschain

**Affiliations:** ^1^ Sanofi Oncology Therapeutic Area, Cambridge, MA, USA; ^2^ Sanofi Translational *In Vivo* Models, Framingham, MA, USA; ^3^ Sanofi Translational Sciences, Cambridge, MA, USA; ^4^ Sanofi Asia Pacific R&D Hub, Shanghai, People’s Republic of China; ^5^ Sanofi Biologics Research/Molecular Screening Technology, Cambridge, MA, USA; ^6^ Sanofi Biologics Research/Nucleic Acid Therapeutics, Frankfurt am Main, Germany; ^7^ Discovery Services, WuXi AppTec Co., Shanghai, China; ^8^ Current address: Leica Biosystems, Boston, MA; ^9^ Current address: Harbour BioMed, Shanghai, People’s Republic of China; ^10^ Current address: Ascentage Pharma Group, Ltd., Suzhou, People’s Republic of China

**Keywords:** hepatocellular carcinoma, β-catenin, cell proliferation, siRNA, phenotypic rescue

## Abstract

Hepatocellular carcinoma (HCC) represents a serious public health challenge with few therapeutic options available to cancer patients.Wnt/β-catenin pathway is thought to play a significant role in HCC pathogenesis. In this study, we confirmed high frequency of CTNNB1 (β-catenin) mutations in two independent cohorts of HCC patients and demonstrated significant upregulation of β-catenin protein in the overwhelming majority of HCC patient samples, patient-derived xenografts (PDX) and established cell lines. Using genetic tools validated for target specificity through phenotypic rescue experiments, we went on to investigate oncogenic dependency on β-catenin in an extensive collection of human HCC cells lines. Our results demonstrate that dependency on β-catenin generally tracks with its activation status. HCC cell lines that harbored activating mutations in CTNNB1 or displayed elevated levels of non-phosphorylated (active) β-catenin were significantly more sensitive to β-catenin siRNA treatment than cell lines with wild-type CTNNB1 and lower active β-catenin. Finally, significant therapeutic benefit of β-catenin knock-down was demonstrated in established HCC tumor xenografts using doxycycline-inducible shRNA system. β-catenin downregulation and tumor growth inhibition was associated with reduction in AXIN2, direct transcriptional target of β-catenin, and decreased cancer cell proliferation as measured by Ki67 staining. Taken together, our data highlight fundamental importance of aberrant β-catenin signaling in the maintenance of oncogenic phenotype in HCC.

## INTRODUCTION

Wnt/β-catenin signaling is an important developmental pathway that is often hijacked by cancer cells to sustain their unrestricted proliferation, promote survival and facilitate metastatic spread. Wnt family of secreted proteins is comprised of 19 members that are ubiquitously expressed and bind to their cognate receptors Frizzled/Lipoprotein Receptor-related Proteins (LRPs) to initiate a series of events leading to stabilization and nuclear entry of intracellular signal transducer β-catenin. Once in the nucleus, β-catenin associates with TCF/LEF family of transcription factors resulting in the expression of a multitude of downstream target genes, including well known oncogenes c-myc, cyclin D1, MET as well as promoters of tumor growth including VEGF, survivin, matrix metalloproteinases (MMPs) and many others (for review of Wnt/β-catenin role in cancer, see [1, 2]).

Under normal physiological conditions, and in the absence of the Wnt signal, β-catenin protein is targeted for ubiquitin-mediated proteasomal degradation by the components of the “destruction box” complex AXIN, adenomatous polyposis coli (APC), glycogen synthase kinase 3b (GSK3b) and casein kinase 1 alpha (CK1a). However, many human malignancies display constitutive activation of Wnt/β-catenin signaling pathway through genetic and epigenetic mechanisms leading to stabilization of β-catenin protein. Frequent somatic alterations in *CTNNB1*, gene that encodes β-catenin, or components of its regulatory network, such as *APC* and *AXIN*, have been detected in human cancers. For example, APC is functionally inactivated in the overwhelming majority of colorectal tumors, as well as in a significant fraction of gastric and uterine cancers [3–5]. Somatic activating mutations in *CTNNB1* have been detected in a number of tumors, but the highest frequency is observed in hepatocellular carcinoma (HCC) [6]. These mutations frequently disrupt normal protein turnover of β-catenin and result in its aberrant accumulation in cancer cells. In addition to canonical Wnt signaling, other important drivers of liver cancer, such as FGF19, depend on β-catenin for their oncogenic activity [7]. HCC is the fifth most common tumor type and the third leading cause of cancer-related deaths world-wide. The incidence of HCC is particularly high in Asia Pacific region due to endemic hepatitis B and C viral infections and is also on the rise in the United States with an estimated 83,000 new cases anticipated by 2030 [8]. HCC is a highly complex, heterogeneous disease due to both viral and non-viral etiology and underlying liver fibrosis and cirrhosis [9]. Importantly, HCC patients still face a shortage of therapeutic options. Surgical resection is often not feasible due to advanced metastatic disease. Multi-kinase inhibitors sorafenib and regorafenib are currently the only approved systemic drugs and are marginally effective in extending survival [10, 11]. Multi-factorial nature of HCC coupled with incomplete understanding of critical drivers of this disease continue to hamper development of new therapeutic modalities for HCC patients [12].

To further enable the development of effective targeted therapies for HCC, a number of groups have conducted detailed molecular characterization of liver cancer based on both gene sequencing and gene expression analysis. These studies revealed a major role for the Wnt/β-catenin pathway in HCC pathogenesis. For example, study by Guichard et al. revealed that more than 30% of HCC harbor somatic activating mutations in *CTNNB1* [13]. Importantly, subsequent genomic profiling of hepatocellular adenomas and HCC demonstrated that somatic activating mutations in *CTNNB1* occur early in HCC pathogenesis and are considered to be a prerequisite for benign hepatic adenoma transformation into frank HCC [14]. Gene expression-based survey of more than 600 patient samples revealed three major molecular classes of HCC termed S1, S2 and S3, one of which (subclass S1) is characterized by strong activation of Wnt pathway gene expression signature and the other (subclass S3) by high frequency of activating *CTNNB1*/ β-catenin mutations [15]. These findings suggest that the vast majority of human HCC display some form of de-regulated Wnt/β-catenin signaling. Furthermore, these data show that Wnt pathway activation may occur both in the presence or absence of genetic alterations in β-catenin.

Studies in animal models of liver cancer have confirmed the central role of Wnt/β-catenin signaling in the onset and maintenance of HCC. Forced overexpression of activated β-catenin in mouse liver progenitor cells results in the development of liver tumors and lung metastases [16]. Similarly, liver-targeted disruption of APC leads to constitutive β-catenin signaling and the onset of HCC in 67% of mice [17]. Finally, similar to the complex nature of human HCC, β-catenin strongly cooperates with other known oncogenes such as MET [18] and AKT [19], in driving HCC tumorigenesis in animal models.

In this study, we aimed to systematically examine activation status of β-catenin at both gene and protein level in a large cohort of human HCC patient samples, as well as in patient-derived xenografts and in established HCC cell lines. Using highly validated genetic tools, we defined the contribution of β-catenin to HCC cell viability *in vitro* and to HCC tumor maintenance *in vivo* in diverse cellular models. Our data provide evidence of wide-spread β-catenin activation in HCC and suggest a correlation between activation status and oncogenic dependency on β-catenin in HCC.

## RESULTS

### β-catenin is frequently altered in HCC patient samples

**Figure 1:**
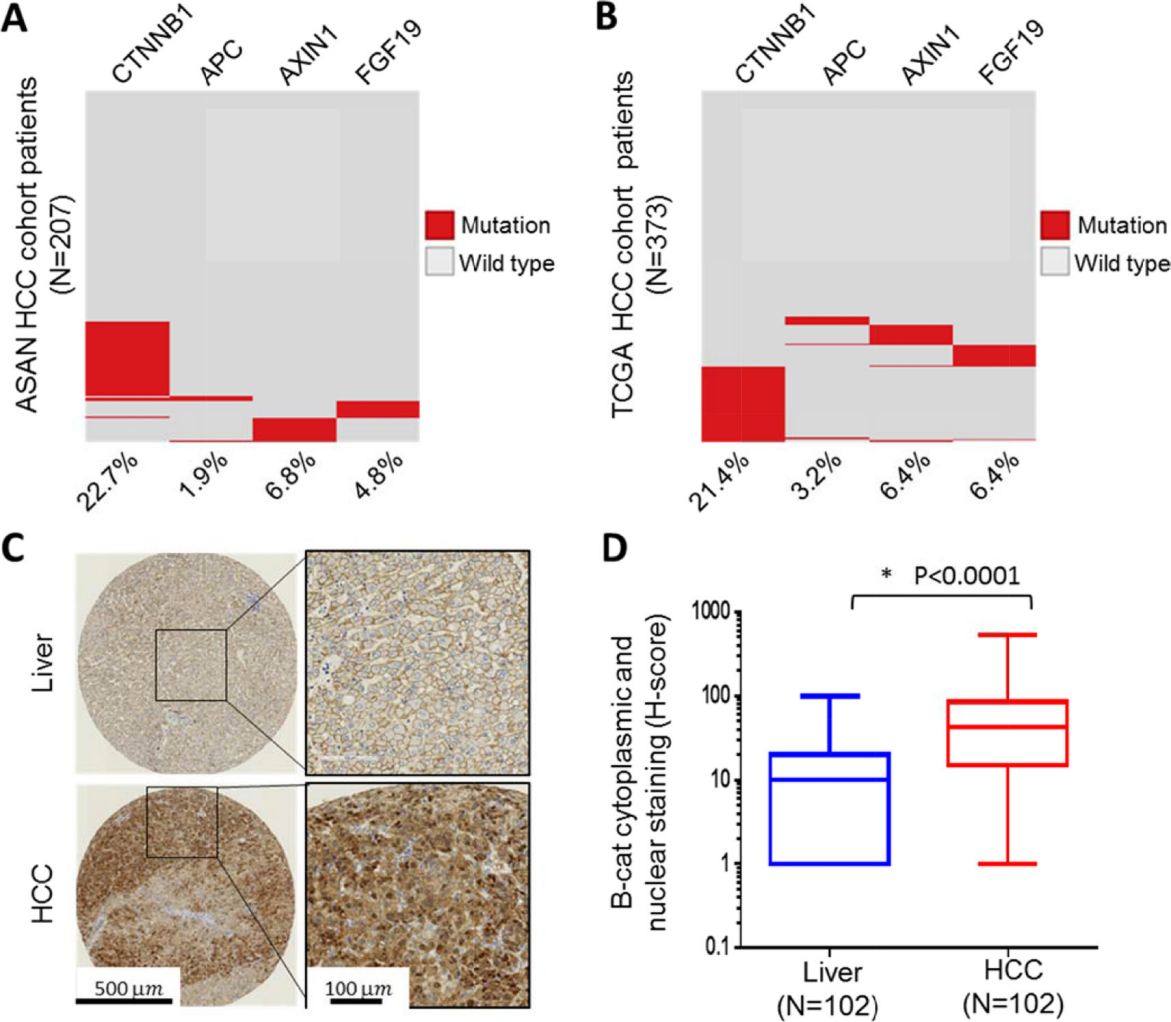
β-catenin is frequently altered in HCC patient samples (B) Frequency of genetic alteration in four genes, *CTNNB1, APC, AXIN1 and FGF19,* were determined in 207 HCC patients from ASAN collection (**A**) and 373 HCC patients from TCGA collection (**B**). Red indicates presence of genetic alteration and gray indicates wild-type. (**C**) Representative immunohistochemical analysis of normal liver and HCC. (**D**) Boxed plot of β-catenin cytoplasmic and nuclear staining (H-score) levels in human normal liver and HCC.

Dysregulation of Wnt/β-catenin signaling has been suggested to play a key role in the pathogenesis of HCC. To evaluate the extent of genomic alterations that might influence β-catenin stability and activity in HCC, we interrogated two large HCC data sets, The Cancer Genome Atlas [3] and HCC cohort from Asan Medical Center [20, 21], for somatic mutations or copy number changes in *CTNNB1*/β-catenin itself, *APC, AXIN and FGF19.* The latter was included since it is part of the 11q13.3 amplicon in HCC and is thought to mediate its oncogenic effects through stabilization of β-catenin [7]. Somatic mutations in β-catenin exceeded 20% in frequency in both data sets (Figure 1A, 1B). Interestingly, non-overlapping genomic changes in *APC, AXIN1* and *FGF1*9, were also identified, albeit with lower frequency. Taken together, these results suggest that genetic alterations in *CTNNB1*/β-catenin itself or its regulatory network constituents occur in > 30% of HCC.

In addition to direct genetic “hits” on *CTNNB1*/β-catenin or components of its regulatory network, it is now well accepted that β-catenin protein stabilization can occur through multiple other mechanisms, including epigenetic changes. As previously mentioned, β-catenin protein is kept at low basal levels in the cell under normal physiological conditions. An increase in cytoplasmic and nuclear β-catenin typically signals increased Wnt/β-catenin pathway activity. To study β-catenin protein abundance in HCC, we acquired 102 paraffin-embedded HCC and adjacent normal liver tissue samples from commercial sources and analyzed them using β-catenin immunohistochemistry. Our data show that in the majority of HCC samples (91 out of 102) β-catenin was elevated in both cytoplasm and nucleus, while in the normal liver, β-catenin staining was localized to cell membrane due to a well-known association with E-cadherin (Figure 1C and Supplementary Table 1). Overall, β-catenin cytoplasmic and nuclear levels were significantly elevated in HCC versus normal liver (Figure 1D). We further investigated whether nuclear β-catenin, which can be considered the most transcriptionally-active fraction, may predict clinical outcome in HCC. Kaplan-Meier analysis revealed a trend toward worse survival for HCC patients with exclusively nuclear β-catenin (“β-catenin activation”) versus those with no detectable nuclear staining (“No β-catenin activation”), although it did not reach statistical significance (log-rank test *p* = 0.08) (Supplementary Figure 1). Cumulatively, our results show wide-spread upregulation of β-catenin protein and an increase in its cytoplasmic and nuclear localization in the overwhelming majority of HCC, likely to due to both genetic alterations and through other mechanisms.

### Majority of HCC cell lines and patient-derived xenografts (PDX) display activation of β-catenin

Our data have demonstrated that *CTNNB1*/β-catenin is often mutated and its protein levels are significantly upregulated in substantial fraction of HCC patient samples. To select appropriate cellular models for functional analysis of β-catenin, we assembled a panel of 25 HCC and cholangicarcinoma (CC) cell lines and probed their cell lysates using antibodies recognizing total as well as non-phosphorylated form of β-catenin. Lack of GSK-3b-mediated phosphorylation on Ser33, Ser37 and Thr41 of β-catenin typically signals resistance to ubiquitin-mediated proteolysis and is thought to mark an “active” β-catenin fraction capable of entering the nucleus and activating downstream gene expression program [22]. As shown in Supplementary Figure 2A and 2B, total β-catenin levels were similar among different HCC cell lines, with the exception of SKHep1 and SNU398 which appeared to have slightly reduced total β-catenin. In contrast, the amount of non-phosphorylated, active β-catenin varied significantly among cell lines, with some displaying significant amount while others having intermediate or low levels. Overall, > 50% of HCC and CC cell lines examined displayed varying degrees of β-catenin activation.

Next we selected a subset of cell lines for further functional studies. Three CC cell lines (OZ, HUCCT1and HUH28) were excluded since cholangiocarcinoma was not in the scope of this investigation. Certain HCC cell lines (SNU423, SNU182, SNU475 and C3A, a derivative of HepG2) that proved difficult to grow and manipulate in culture (data not shown) were excluded as well. We decided to focus on 18 HCC cell lines with known genetic alterations in β-catenin as well as on those with “high”, “intermediate” and “low” levels of active, non-phosphorylated β-catenin (Supplementary Figure 2C). For 18 HCC cell lines chosen, we attempted to map potential causes of β-catenin activation based on publically available genomic profiling data. For example, search of the Cancer Cell Line Encyclopedia [23] revealed that HUH6 and SNU398 harbor somatic activating mutations in *CTNNB1*/β-catenin, while HepG2 carries a deletion in exons 3–4 of *CTNNB1*/β-catenin gene resulting in the deletion of amino acids 25–140. These somatic alterations interfere with β-catenin phosphorylation and protein turnover and lead to its constitutive activation. As a consequence, the antibody specific for non-phosphorylated, active β-catenin fails to recognize these mutant proteins. In contrast, Hep3B, JHH5, JHH6 and SNU449 are known to harbor mutations in AXIN1, a critical component of β-catenin degradation machinery, which leads to stabilization and accumulation of β-catenin protein. Finally, Huh7, JHH7 and SNU387, contain 11q13.3 amplification, which includes *FGF19*, a known positive mediator of β-catenin activity [7]. Three out of four HCC cell lines with 11q13.3 amplification displayed high or intermediate levels of “active”, non-phosphorylated β-catenin. For the remaining HCC cell lines with high or intermediate activated β-catenin, we were not able to identify a clear genetic cause based on publically available information. This is consistent with published reports suggesting that multiple genetic and epigenetic factors may converge on β-catenin to increase its protein stability and transcriptional activity [24].

To confirm that increased abundance of non-phosphorylated β-catenin detected in HCC cell lines accurately reflects real disease setting, we interrogated a number of HCC PDX [25] using the same set of β-catenin-specific antibodies. Similar to HCC cell lines, the majority of HCC PDX were positive for non-phosphorylated β-catenin (Supplementary Figure 3A, 3B). Out of 15 HCC PDX analyzed, three were mutant for β-catenin, thus closely matching the frequency of β-catenin somatic alterations found in HCC patient samples (∼20%). Copy number loss in *AXIN1* and amplification in *FGF19* were also detected in HCC PDX (Supplementary Figure 3C). However, genetic alterations in Wnt/β-catenin pathway once again could not account for all instances of β-catenin activation in either HCC cell lines or in PDX, which is consistent with the data obtained in HCC patient samples.

### *In vitro* dependency on β-catenin tracks with its activation status

**Figure 2:**
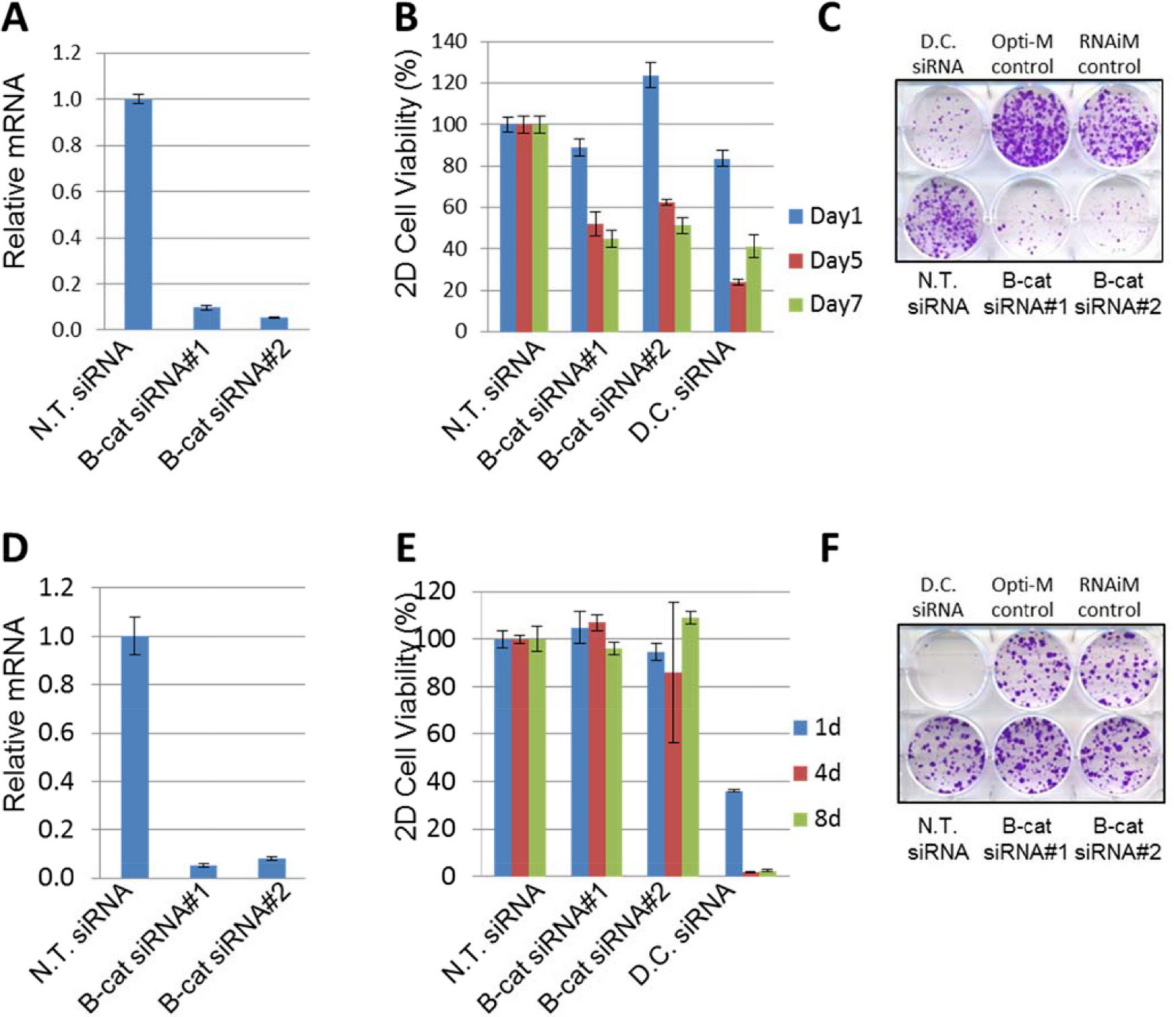
Silencing of β-catenin in HUH6, but not in SNU387 cells, leads to cell growth inhibition (**A**) Taqman analysis of β-catenin target knock-down by two independent siRNAs compared to N.T. siRNA in HUH6 cells. (**B**) β-catenin knock-down by two independent siRNAs inhibits viability of HUH6 cells. (**C**) Representative 2D colony formation assay in HUH6 cells. (**D**) β-catenin knockdown by two independent siRNAs compared to the N.T. siRNA in SNU387 cells. (**E**) β-catenin knockdown by two independent siRNAs does not inhibit the viability of SNU387 cells. (**F**) Representative 2D colony formation assay in SNU387 cells. N.T. siRNA stands for non-targeting siRNA. D.C. siRNA stands for Death Control siRNA.

Next we asked if oncogenic dependency on β-catenin can be linked to its activation status. To investigate this, we initially selected two HCC cell lines: HUH6, which harbors G34V somatic activating mutation in *CTNBB1*/β-catenin, and SNU387, which is *CTNNB1*/β-catenin wild-type and has low levels of active β-catenin protein. Both cell lines were transfected with two independent siRNAs specific for β-catenin and target knock-down was analyzed using qRT-PCR. Both siRNAs were highly effective in reducing β-catenin levels in HUH6 and SNU387 cells (Figure 2A and 2D). The impact of β-catenin depletion was studied in cell viability/proliferation and colony formation assays. Knock-down of β-catenin in HUH6 cells resulted in significant inhibition of cell proliferation (Figure 2B), as well as suppression of colony formation (Figure 2C). In contrast, no measurable effect on cell viability was observed in SNU387 cells upon β-catenin knock-down (Figure 2E and 2F). Non-targeting (N.T.) siRNA was included as a negative control and did not alter proliferation in either cell line, while Death Control (D.C.) siRNA targeting a number of human genes essential for cell survival compromised proliferation and survival in both cell lines, as expected. Taken together, these results suggest that HCC cells with activated β-catenin could be more susceptible to β-catenin knock-down than HCC cell lines in which β-catenin is under normal physiological control.

**Figure 3:**
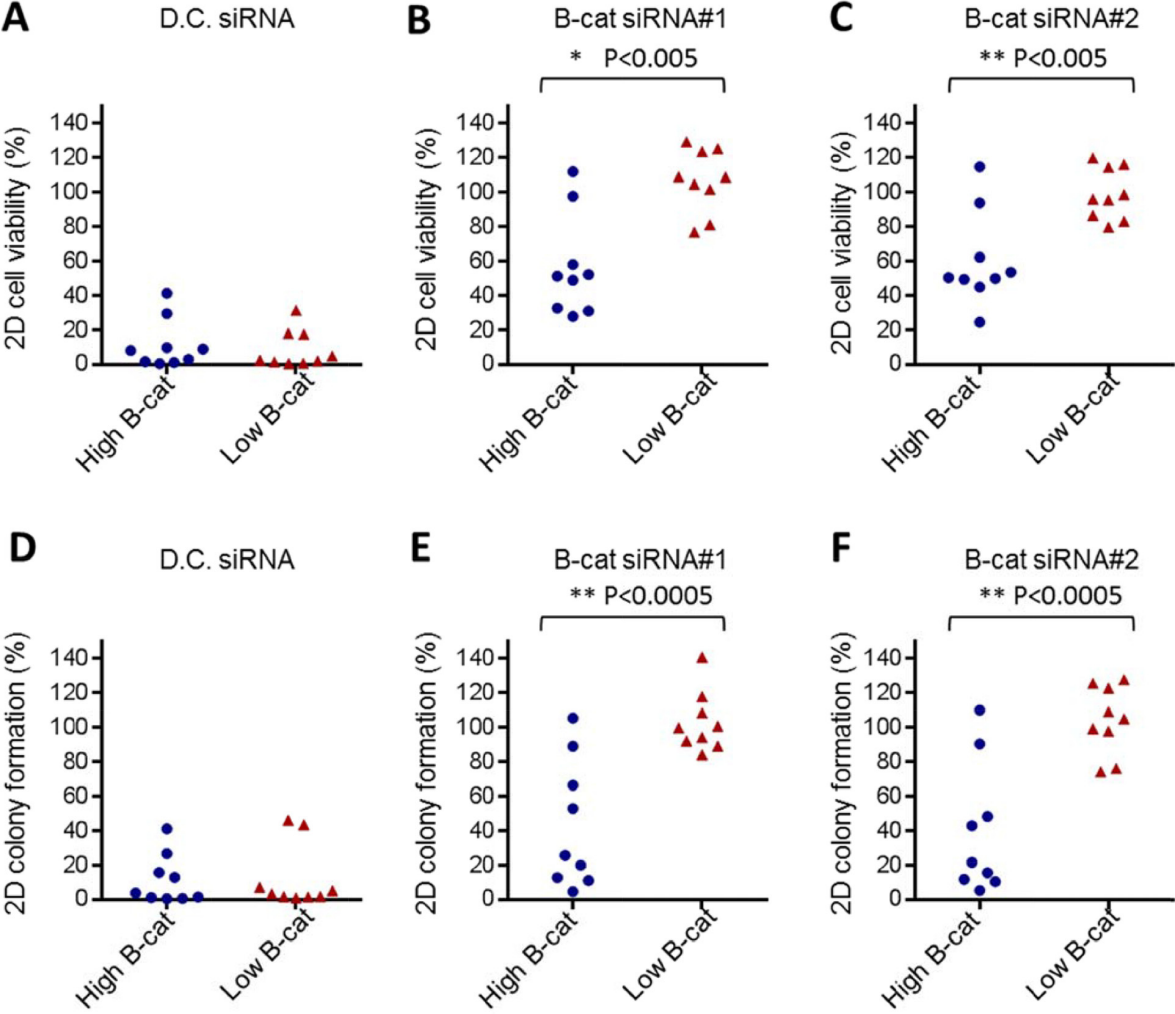
*In vitro* dependency on β-catenin tracks with its activation status Cumulative cell viability data obtained in 10 “high” active β-catenin HCC cell lines and 9 “low” or “intermediate” active β-catenin HCC cell lines using Death Control siRNA (**A**) or two independent β-catenin siRNAs, #1 (**B**) and #2 (**C**). Cumulative 2D colony formation data obtained in 10 “high” active β-catenin HCC cell lines and 9 “low” or “intermediate” active β-catenin HCC cell lines treated with Death Control siRNA (**D**) or two independent β-catenin siRNAs, #1 (**E**) and #2 (**F**). D.C. siRNA stands for Death Control siRNA.

To determine if our observations could be extended to a larger panel of HCC cell lines, we screened an additional 16 HCC cell lines by using similar siRNA-based approach. In each instance, β-catenin knock-down was confirmed by qRT-PCR and cell proliferation and clonogenic survival was monitored over time. In all cell lines tested, two β-catenin siRNAs used in this study were able to reduce β-catenin levels by 80–90%. However, phenotypic consequences of β-catenin knock-down varied significantly among HCC cell lines (Supplementary Figures 4–19). To better interpret the data, we established a threshold of ≥ 40% reduction in cell proliferation or colony formation (when compared to non-targeting siRNA) as a criteria to classify HCC cell line as “responder” to β-catenin knock-down. Among 18 HCC cell lines tested (including previously analyzed HUH6 and SNU387), seven were classified as “responders” and eleven were designated as “non-responders” (Supplementary Table 2). Strikingly, all HCC cell lines with low to intermediate levels of activated β-catenin were completely refractory to phenotypic effects of β-catenin siRNA. In contrast, the majority of HCC cell lines with high activated β-catenin were deemed responsive to β-catenin knock-down. All HCC cell lines tested were equally affected by the “death control” siRNA (Figure 3). These data demonstrate that sensitivity to β-catenin inhibition may be associated with its activation status in HCC cell lines.

### Phenotypic effects of β-catenin siRNA are on-target

Although we utilized two independent β-catenin siRNAs in our study, this still does not rule out potential off-target effects of RNAi. To confirm that genetic tools used for screening of HCC cell lines are on-target, we embarked on phenotypic rescue experiments. To accomplish this, we introduced silent mutations into cDNA construct encoding β-catenin to make the resulting mRNA resistant to siRNA#1 of β-catenin. We hypothesized that if growth-suppressing effects of siRNA#1 in “responder” HCC cell lines are in fact due to β-catenin depletion and not because of dysregulation of unrelated targets, then the expression of siRNA-resistant cDNA in these cells would maintain β-catenin levels and downstream signaling and protect them from anti-proliferative effects of β-catenin knock-down. Alternatively, if anti-proliferative activity of β-catenin siRNA#1 is the result of unintended target knock-down, then the siRNA#1-resistant cDNA would not be able to restore cell proliferation.

**Figure 4:**
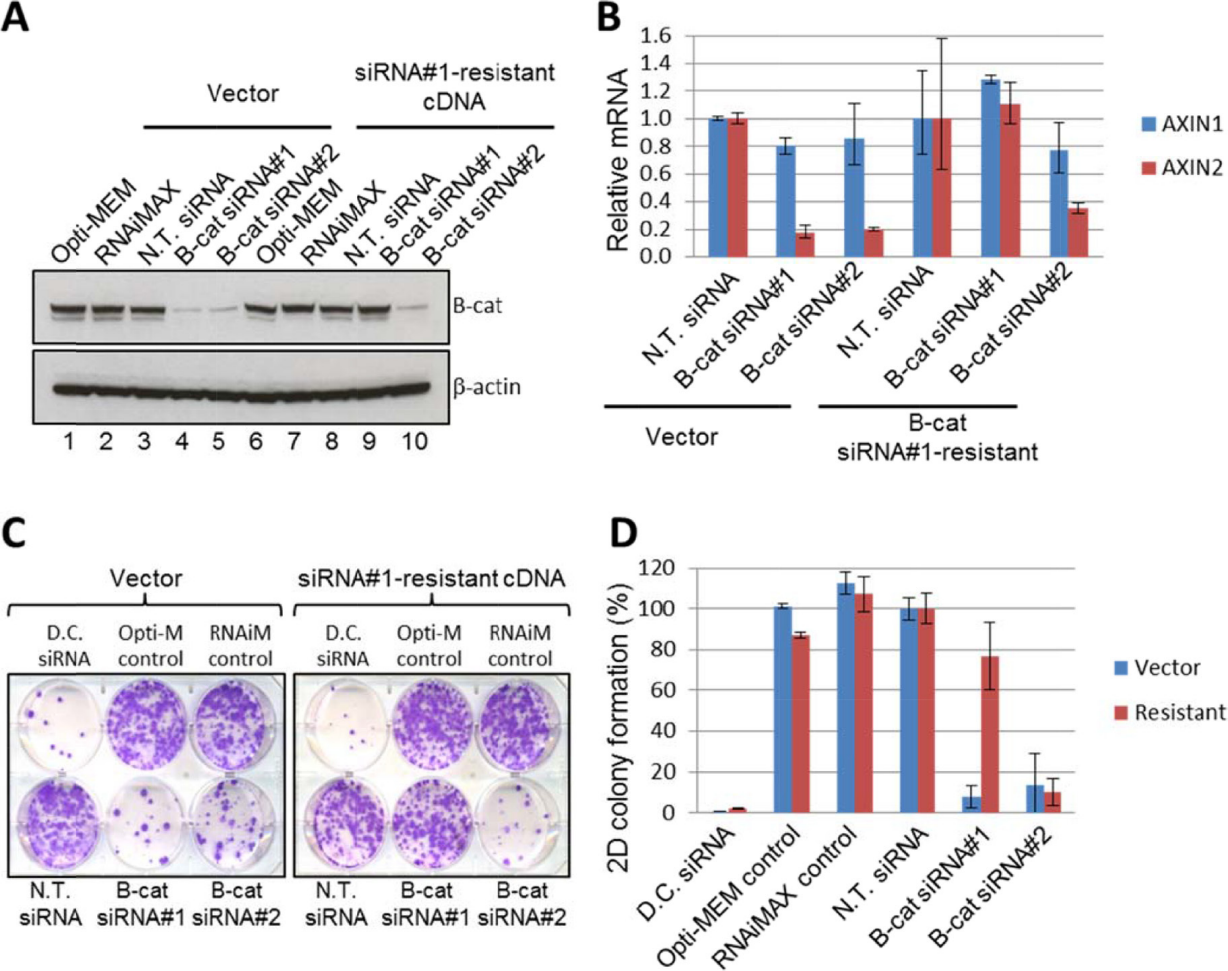
Phenotypic effects of β-catenin siRNA are on-target in JHH5 cells (**A**) Exogenous expression of siRNA#1-resistant β-catenin cDNA restored β-catenin protein levels upon endogenous β-catenin knockdown by siRNA #1, but not siRNA #2. Empty vector control did not restore β-catenin protein levels upon endogenous β-catenin knockdown by siRNA#1 or siRNA#2. (**B**) Exogenous expression of siRNA#1-resistant β-catenin cDNA restored mRNA expression of downstream target of β-catenin, *AXIN2*, upon endogenous β-catenin knockdown by siRNA#1, but not siRNA#2. The empty vector control did not recover *AXIN2* mRNA upon endogenous β-catenin knockdown by siRNA#1 and siRNA#2. *AXIN1* gene is not subject to regulation by β-catenin and was used as an additional control. (**C–D**) Expression of siRNA#1-resistant β-catenin restored the viability of cells after endogenous β-catenin knockdown, whereas the empty vector control did not. Representative 2D colony formation plates (C) and quantification of colony formation (D) are shown.

“Responder” JHH5 cells were stably transduced with either empty vector or siRNA#1-resistant β-catenin cDNA and subsequently transfected with β-catenin siRNA#1 and #2. As depicted in Figure 4A, β-catenin protein levels were substantially reduced by both siRNA#1 and #2 in vector-expressing JHH5 cells. In contrast, exogenous β-catenin protein was left virtually intact in JHH5 cells expressing siRNA#1-resistant cDNA when they were exposed to siRNA#1, while the same cells treated with siRNA#2 displayed significant β-catenin knock-down. Similar effects were observed when the levels of direct transcriptional target of β-catenin, *AXIN2*, were investigated by qRT-PCR (Figure 4B). Collectively, these data indicate that siRNA#1-resistant cDNA construct is fully functional in JHH5 cells and is capable of maintaining β-catenin levels and activity in the presence of siRNA#1, but not siRNA#2.

Next, we investigated the response of vector- and siRNA#1-resistant JHH5 cells to the treatment with β-catenin siRNA#1 and #2 in the colony formation assay. Long-term proliferation and colony-forming ability of vector-expressing JHH5 cells was significantly inhibited by both β-catenin siRNAs, once again confirming JHH5 as a “responder” cell line. However, in siRNA#1-resistant JHH5 cells, only siRNA#2 was effective in suppressing cell proliferation, while the growth of cells transfected with siRNA#1 was unaffected (Figure 4C, colony quantification is shown in Figure 4D). Taken together with β-catenin knock-down data and downstream target modulation results, these observations indicate that phenotypic effects of β-catenin siRNA#1 are in fact on target. Similar phenotypic rescue was observed in an additional “responder” line Hep3B (Supplementary Figure 20), thus further solidifying our conclusions.

### Inducible knock-down of β-catenin suppresses growth of HCC tumor xenografts

Having established oncogenic dependency on β-catenin in a number of HCC cell lines *in vitro*, we aimed to investigate the contribution of β-catenin signaling to the maintenance of HCC tumors *in vivo*. To this end, we converted sequences of siRNA#1 and #2 to doxycycline-inducible shRNAs. Stable shRNA cell lines were established in “responder” Hep3B cell line via lentiviral transduction and β-catenin knock-down was confirmed by immunoblot after 3 days of incubation with doxycycline. Induction of shRNA expression resulted in significant downregulation of β-catenin mRNA (Supplementary Figure 21A) and protein (Supplementary Figure 21B). In addition, downstream target of β-catenin *AXIN2* was substantially reduced in doxycycline-treated Hep3B cells (Supplementary Figure 21C). Since sequence of siRNA#1 has already been shown by us to act on target, we chose corresponding inducible shRNA#1 for further experiments *in vivo*.

**Figure 5:**
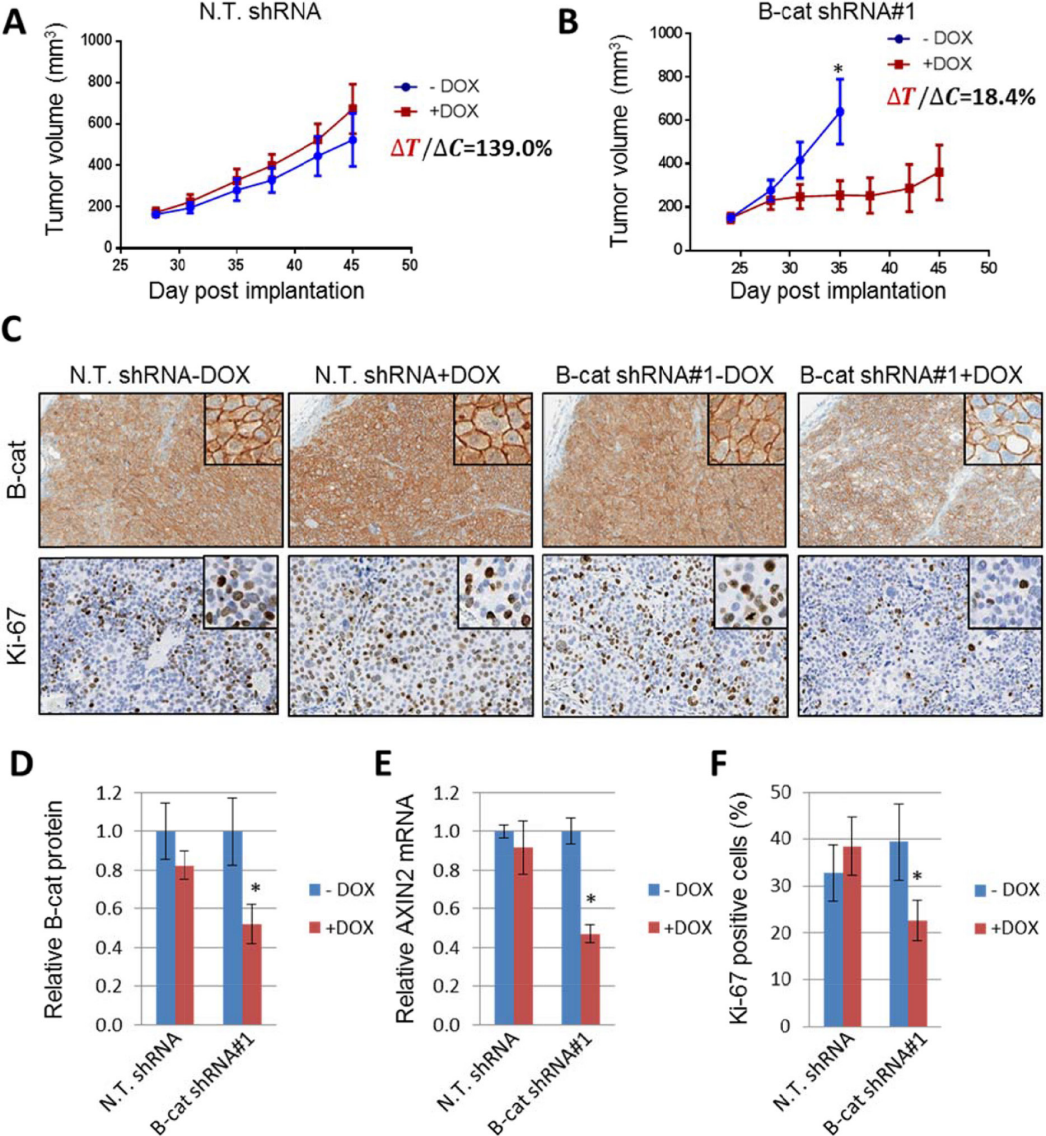
Inducible knock-down of β-catenin suppresses growth of Hep3B HCC tumor xenografts Tumor growth of Hep3B xenografts expressing non-targeting shRNA (**A**) or β-catenin shRNA#1 (**B**). Tumor volume was measured on indicated days as described in Materials and Methods (*n* = 8 per group). Error bars represent SD of the mean. ^*^p < 0.05. (**C**) IHC for β-catenin and proliferation marker Ki-67 in Hep3B tumors expressing non-targeting shRNA or β-catenin shRNA#1, with or without doxycycline treatment. (**D**) Quantification of β-catenin immunoblot analysis of Hep3B tumors expressing non-targeting shRNA or β-catenin shRNA#1, with and without doxycycline treatment. (**E**) Taqman analysis of *AXIN2* levels in Hep3B tumors upon induction of β-catenin knock-down. (**F**) Quantification of IHC staining for Ki-67 displayed as a percentage of cells positive for Ki-67 staining. Error bars represent SD of the mean. ^*^p < 0.05.

Hep3B cells expressing either β-catenin shRNA#1 or non-targeting control were implanted s.c. into immunocompromised mice and tumors were allowed to establish. Once tumors reached 150–200 mm^3^, β-catenin knock-down was triggered by feeding the mice doxycycline-containing food. Tumors expressing non-targeting shRNA continued to grow unabated in the presence or absence of doxycycline (Figure 5A). In sharp contrast, β-catenin shRNA-expressing tumors that were exposed to doxycycline grew much slower than those receiving standard chow (Figure 5B).

At the end of the study, the tumors were analyzed for β-catenin protein levels using immunohistochemistry (Figure 5C) and immunoblot (Figure 5D). In both instances, we were able to detect significant target knock-down in β-catenin shRNA-expressing tumors that had been treated with doxycycline, but not in any other samples. Interestingly, β-catenin membrane staining was largely preserved in tumor tissue samples, while cytoplasmic and nuclear β-catenin was significantly reduced in dox-treated β-catenin shRNA tumors. Additional analysis revealed downregulation in *AXIN2* mRNA in β-catenin shRNA samples exposed to doxycycline, indicating suppression of downstream β-catenin signaling (Figure 5E). Finally, Ki67 staining, a marker of cell proliferation, was reduced in dox-treated β-catenin shRNA tumors (Figure 5C and 5F). Similar results were obtained in HepG2 tumor xenografts, thus ruling out model-specific effects of β-catenin shRNA (Supplementary Figure 22). Collectively, these data establish an important role for β-catenin in the maintenance of HCC tumors *in vivo*.

## DISCUSSION

HCC is a complex human malignancy characterized by deregulation of multiple signaling pathways, among which Wnt/β-catenin occupies the place of central importance. This is due to high frequency of somatic activating mutations in *CTNNB1*/β-catenin itself or components of its regulatory network. In this study we systematically investigated activation status of β-catenin in HCC patient samples, primary tumor xenografts and a large panel of HCC cell lines. We confirm and extend previous observations on the high incidence of *CTNNB1*/β-catenin activating mutations in HCC. In addition, the frequency of Β-catenin genetic alterations in human PDX and established cell lines in our study was similar to what we observed in HCC patient samples (∼20%), suggesting translational relevance of these disease models. Our results also highlight the fact that genetic alterations are not the only cause of β-catenin activation in HCC as accumulation of β-catenin protein was also observed in the absence of detectable somatic mutations in *CTNNB1* gene.

Nuclear β-catenin has been previously identified as a powerful predictor of overall survival and clinical outcomes in a number of human cancers, including colorectal [26–28], non-small cell lung cancer [29], and other malignancies. In our study, exclusively nuclear β-catenin was detected in 9 HCC patients out of 102 analyzed. This may have contributed to a trend rather than statistically significant association with poor survival in HCC. Nonetheless, our results are in line with the earlier meta-analysis of 22 studies containing 2334 HCC cases that identified cytoplasmic and/or nuclear accumulation of β-catenin as an independent prognostic factor in HCC that is significantly associated with poor prognosis [30]. Our data now extend these findings to an additional independent cohort of 102 HCC patients.

Cognizant of multiple genetic and epigenetic events that may lead to β-catenin activation, we decided to focus on non-phosphorylated (active) form of β-catenin and use it as a marker of pathway activation. In our studies, we utilized an antibody that specifically recognizes non-phosphorylated β-catenin to screen a large panel of HCC cell lines and PDX models. Since established cell lines are more amenable to manipulation in culture, we deployed siRNAs to establish oncogenic dependency on β-catenin in these models. Importantly, in each cell line screened we documented both β-catenin target knock-down and phenotypic effects of siRNA in two different assays (cell viability and clonogenic survival). Since siRNA activity is sometimes associated with off-target effects that confound cell growth data interpretation [31], we embarked on a thorough validation of our genetic tools using phenotypic rescue experiments. We utilized the most rigorous approach possible - overexpression of siRNA-resistant cDNA - to restore target expression, PD marker and cell viability in siRNA-treated cells. Our results unequivocally demonstrate specificity of siRNA#1 used to knock-down β-catenin in our study. Since this siRNA is commercially available, other researchers can now utilize this sequence in their studies with high degree of confidence.

Our data suggest a link between the presence of activating mutations in CTNNB1 or increased “active” β-catenin levels and sensitivity to β-catenin ablation. To our knowledge, this is the first study that systematically examined both activation status and oncogenic dependency on β-catenin in such an extensive panel of human HCC cell lines. Survival of all HCC cell lines harboring somatic alterations in *CTNNB1*/β-catenin was significantly affected by β-catenin siRNA in our study. Our results also demonstrate that dependency on β-catenin is not restricted to mutant β-catenin HCC cells, but extends to many contexts where “active” β-catenin protein is elevated. While not all “high” cell lines active β-catenin were sensitive to β-catenin knock-down, the majority displayed reduced survival and proliferation when treated with β-catenin siRNA. The rest of the HCC cell lines with elevated active β-catenin levels likely depend on other oncogenic drivers. For example, SNU449 cells, in addition to *AXIN1*, harbor genetic alterations in tumor suppressor *PTEN* leading to reduced PTEN protein expression, increased levels of phospho-AKT (Ser473) and significant sensitivity to inhibitors of PI3K/mTOR signaling [32]. Similarly, another *AXIN1*-mutant HCC cell line, JHH6, has been reported to contain deletions in exon 2 of *NFE2LD* (Nrf2) and display strong oncogenic dependence on Nrf2 [33].

We extended our *in vitro* findings to HCC tumor xenografts *in vivo*, once again utilizing genetically validated inducible shRNA to knock-down β-catenin. The use of inducible shRNA system in these studies provided crucial evidence for the role of β-catenin in HCC tumor maintenance rather than in xenograft implantation. This implies that targeting of β-catenin in human HCC may have therapeutic benefit. Taken together with previously published data, our results establish an important role for β-catenin in human HCC. They also suggest that, in addition to somatic activating mutations in *CTNNB1,* non-phosphorylated β-catenin can be used as a patient selection marker. This approach may significantly broaden the reach of β-catenin-directed therapies in HCC.

The development of potent and specific β-catenin therapeutics continues to represent a significant challenge. Activated β-catenin is a transcriptional modulator that exerts its effects in the nucleus by altering the expression of genes associated with cell proliferation and survival. Attempts to directly target β-catenin with low molecular weight inhibitors have not succeeded thus far. Stapled peptides for β-catenin have been reported [34], but their activity in relevant tumor models *in vivo* has not been demonstrated. Therapeutic siRNA represents an appealing approach to directly inhibit β-catenin [35, 36]; however, convincing evidence of RNAi delivery to human tumors in a clinical setting is still lacking. Given the unequivocally important contribution of β-catenin to pathogenesis of HCC and other human malignancies, novel strategies for direct targeting of this oncogene need to be developed.

## MATERIALS AND METHODS

### Assessment of genetic alterations in key genes in Wnt/β-catenin pathway

Frequencies of genetic alterations, including non-synonymous mutations, small indels and copy number amplification, for genes in the Wnt pathway (*CTNNB1, APC, AXIN1*), as well as *FGF19*, were determined based on whole-exome next-generation sequencing data and comparative genomic hybridization (CGH) data from two HCC patient cohorts: TCGA [6] and ASAN [20, 21]. TCGA cohort contains 373 patients whereas ASAN dataset includes 207 patients. For *CTNNB1* gene, only activating mutations or in-frame deletions in exon 2/3 region were counted. For *FGF19*, only copy number amplification (defined as copy number > = 5) was considered.

### Immunohistochemistry (IHC)

IHC for β-catenin was carried out using β-catenin (D10A8) XP^®^ antibody (rabbit mAb, catalog #8480, Cell Signaling; 1:100, AR in EDTA) on 4 μm sections of human Hep3B and HepG2 liver xenografts and human HCC TMAs. HCC TMAs (sets 1 & 2, Supplementary Table 1), comprised of 153 dots each, were obtained from Pantomics and represent 51 HCC cases per TMA. TMA IHC staining was carried out at CrownBio. Briefly, TMA sections were obtained using IHC procedures on Bond RX. Immunostained slides were scanned with Nanozoomer –HP2.0 Digital Slide System Hamamatsu Image system on Bond RX automatic IHC&ISH system (Leica). The intensity of membrane, cytoplasmic or nuclear β-catenin staining was scored at four levels, 0 (negative), 1 (weak staining), 2 (medium staining), 3 (strong staining). The percentages of tumor cells at different intensity levels were determined. Total H Score = (% at 0)×0 + (% at 1)×1 + (% at 2)×2 + (% at 3)×3.

For IHC of Ki-67, unstained sections of formalin-fixed paraffin-embedded sections of Hep3B tumor samples were subjected to bright field IHC using BondMax automated immunostainer (Leica) as described previously [37]. A rabbit anti-mouse Ki-67 monoclonal antibody clone SP16 (Abcam; catalog # ab16667) was used as the primary antibody and a rabbit IgG (R&D Systems; catalog #AB-105-C) was used as an isotype control. Nuclear immunostaining was visualized using the DAB-based Bond Polymer Refine detection kit (Leica); hematoxylin was used as a counterstain. Immunostained slides were scanned using Aperio Scanscope XT slide scanner (Leica). Multiple non-overlapping tumor regions within each tumor were analyzed for Ki-67-immunopositive cells by using automated cytonuclear algorithms in HALO image analysis software (Indica Labs).

### Survival analysis

IHC was performed to detect β-catenin protein in the nucleus, cytoplasm and cellular membrane in 102 HCC patients as described above. Any patient with detectable nuclear β-catenin staining was considered as having “Bcat activation” and otherwise as “No Bcat activation”. Based on IHC results, 9 patients were assigned to “Bcat activation” group and 93 were assigned to “No Bcat activation” group. Log-rank test was performed to compare the survival distribution of the two groups and Kaplan-Meier curves were plotted.

### Cell culture

HCC cell lines were obtained from the DSMZ, ATCC or JCRB. Short Tandem Repeat (STR) DNA profiling was performed to verify cell identity. Cells were grown in DMEM (Invitrogen), supplemented with 10% FBS (Invitrogen), and cultured at 37°C in a humidified incubator with 5% CO_2_.

### Lentivirus preparation

Lentiviral constructs (shRNA or cDNA) were packaged in HEK293T cells using the Trans-Lentiviral packaging kit (Open Biosystems). Virus-containing media were collected and filtered. Target cells were transduced at a multiplicity of infection of 1 in the presence of 8 μg/ml polybrene. After a 24 h infection, the culture media was supplemented with puromycin at a final concentration of 1 μg/ml. The cells were selected for 5 days.

### siRNA transfections and lentivirus-mediated shRNA knockdown

siRNAs used in this study are listed in Supplementary Table 3. Lyophilized siRNA were reconstituted in nuclease-free water. Lipid-mediated transfection was performed in a 6-well tissue culture plate using RNAiMAX (Thermo Fisher Scientific). Briefly, 9 μl of RNAiMAX was mixed with 291 μl OptiMEM^®^ (Thermo Fisher Scientific). This mixture was combined with 300 μl of siRNA mixture prepared in OptiMEM^®^. The RNAiMAX*/*siRNA mixture was incubated for 10 min, and the entire 600 μl was added to a single well of a 6-well tissue culture plate. Cells (6 × 10^5^ in 2400 μl of growth media) were added to the transfection mixture. At 24 h post-transfection, the media were replaced with fresh culture medium. After an additional 24 h, the cells were trypsinized and suspended at a dilution of 5 × 10^4^ cells*/*ml and 100 μl of cell suspension was dispensed into duplicate 96-well plates.

shRNAs used in this study are listed in Supplementary Table 3. HepG2 and Hep3B cells with β-catenin shRNAs and non-targeting control shRNA were established using the pLKOpuro_U6-TO_TetR lentiviral system as described above. Stable cell lines were either left untreated (−DOX) or treated with doxycycline (+DOX) to induce expression of shRNA and cell viability was monitored as described herein.

### Cell viability/ proliferation assay

Cell viability/proliferative expansion was assessed by the addition CellTiter-Glo^®^ reagent (Promega) to cell cultures for 10 minutes. Endpoint readings were collected using SpectraMax Pro V5 plate reader (Molecular Devices). The intensity of the luminescence signal was directly proportional to the number of live cells.

### Two-dimensional (2D) colony formation assay

Approximately 4000 cells that were previously transfected with siRNAs were seeded into each well of a 6-well plate. After incubation at 37°C for 12∼14 days, the cells were washed with PBS twice, fixed with methanol and then stained with 0.1% crystal violet.

The plates were scanned for quantification. Full-well brightfield images were acquired on a Nexcelom (formerly Cyntellect) Celigo automated microscope. Images were analyzed in Definiens (Definiens AG, Munich, Germany, Developer version 2.1) using a custom-developed ruleset: Images were scaled down to 25%, initial colony objects were identified with the auto threshold algorithm. Artifacts and non-colony objects were subsequently filtered out and the number and area of colony objects was exported.

### siRNA phenotypic rescue experiments

For rescue experiments, silent mutations were introduced into cDNA encoding β-catenin (siRNA#1 resistant) in plenti9_IRES-BlastR_DEST (CR10383, RA10563027: from CTGTTGGATTGATTCGAAATC changed to CCGTAGGCCTCATCAGGAACC). JHH5 and Hep3B cells stably expressing siRNA#1-resistant β-catenin cDNA or empty vector control were established using the pLEN.T.I9-IRES_BLASTR lentiviral expression system. Lentiviral constructs were packaged in HEK293T cells using the Trans-Lentiviral packaging kit (Open Biosystems). Virus-containing media were collected and filtered. JHH5 and Hep3B cells were infected with the virus at multiplicity of infection of 1, followed by blasticidin selection (6 g/ml). Stable cell lines were then transfected with N.T. siRNA and β-catenin siRNAs, and the cells were monitored for viability as described above.

### RNA isolation and real-time quantitative polymerase chain reaction (RT-qPCR)

RNA was isolated using the 96RNeasy kit (Qiagen), following the vacuum*/*spin protocol without the optional DNase treatment. RNA was eluted into nuclease-free water and quantified by ultraviolet absorbance using a NanoDrop 8000 (Thermo Scientific). cDNA synthesis was performed using the High Capacity RNA to cDNA Kit (Applied Biosystems) according to the manufacturer’s recommendations. The cDNA was diluted 1:4 with nuclease-free water and 2.5 μl was added to a 9 μl Taqman reaction mixture prepared using the TaqMan Gene Expression Master Mix (Applied Biosystems). Taqman assays were performed on a ViiATM7 (Applied Biosystems) or a QuantStudioTM 12K Flex Real Time PCR System (Applied Biosystems) using the following conditions: 50°C for 2 min; 95°C for 10 min; 40 cycles of 95°C for 15 s and 60°C for 1 min. Gene expression was normalized to the endogenous control GAPDH for mRNA expression. Taqman primers and probes are listed in Supplementary Table 4.

### Immunoblotting

Total cell lysates were prepared in RIPA Buffer (Pierce) supplemented with HALT Protease and Phosphatase Inhibitor (Pierce). Lysates were pelleted at 12 000 × *g* for 20 min and the supernatant was collected. Protein lysates were quantified using a Bicinchoninic Acid Assay (Pierce). Protein lysates were mixed with NuPAGE LDS Sample Buffer (Life Technologies), loaded onto a 4–12% Bis-Tris gradient gel (Life Technologies), and subjected to electrophoresis in MES buffer (Life Technologies). The proteins were transferred to a nitrocellulose membrane using an iBlot gel transfer device (Life Technologies). The nitrocellulose membrane was blocked with a 5% non-fat milk solution prepared in TBS-Tween (Cell Signaling). Primary antibodies to total β-catenin (1:1000, #9562, Cell Signaling), active non-phoshpo-β-catenin (1:1000, #4270, Cell Signaling), β-Actin (1:5000, sc-69879, Santa Cruz) were incubated overnight at 4°C with rocking in 2% non-fat milk*/*TBS-Tween solution. Membranes were extensively washed and incubated with secondary antibody (1:5000, Pierce). Membranes were developed with Super SignalWest Pico Chemiluminescent Substrate (Pierce) and exposed to film. Quantification of the Western blot analysis was performed using ImageJ computer program.

For immunoblot analysis of Hep3B and HepG2 tumor lysates, tissue extracts were separated by an automated capillary-based electrophoresis system (WES, ProteinSimple). All procedures were performed according to the manufacturer’s recommendations using the supplied reagents. Briefly, after determination of protein concentration of each lysate, 1 μg of total protein (4 μL) was mixed with 1 μL of 5X fluorescent master mix and heated at 95°C for 5 min. The samples, blocking reagent, wash buffer, primary antibodies, secondary antibodies, and chemiluminescent substrate were dispensed into designated wells in the manufacturer provided microplate. Primary antibodies used are as follows: total β-catenin (1:100, #8480, Cell Signaling), pan-actin (1:100, #8456s, Cell Signaling). Following plate loading, the separation and immuno-detection was performed automatically using default settings. The resulting data were analyzed using Compass software (ProteinSimple). β-catenin signal was normalized by pan-actin signal.

### Animal handling

All *in vivo* experiments were performed according to the institutional guidelines and approved by the Institutional Animal Care and Use Committee. 5 × 10^6^ Hep3B or HepG2 cells stably expressing doxycycline-inducible β-catenin shRNA#1 or Control (non-targeting shRNA) were suspended in 50% matrigel and implanted subcutaneously (s.c.) into a flank of 9∼11 week old female Severe Combined Immuno Deficiency (SCID) mice (Charles River Laboratories). The tumors were allowed to establish until average tumor volume reached about 150 mm^3^. Subsequently mice were randomized into two groups (*n* = 8/group), and the +DOX group mice were switched to 400 ppm doxycycline diet (Harlan), while animals in the -DOX group were maintained on standard mouse diet. Tumor growth was monitored over time using caliper measurements. Subcutaneous tumor volume was calculated using the formula: length × width^2^/2.

## SUPPLEMENTARY MATERIALS FIGURES AND TABLES





## References

[1] Clevers H, Nusse R (2012). Wnt/β-catenin signaling and disease. Cell.

[2] Zhan T, Rindtorff N, Boutros M (2017). Wnt signaling in cancer. Oncogene.

[3] Muzny DM, Bainbridge MN, Chang K, Dinh HH, Drummond JA, Fowler G, Kovar CL, Lewis LR, Morgan MB, Newsham IF, Reid JG, Santibanez J, Shinbrot E, Cancer Genome Atlas Network (2012). Comprehensive molecular characterization of human colon and rectal cancer. Nature.

[4] Bass AJ, Thorsson V, Shmulevich I, Reynolds SM, Miller M, Bernard B, Hinoue T, Laird PW, Curtis C, Shen H, Weisenberger DJ, Schultz N, Shen R, Cancer Genome Atlas Research Network (2014). Comprehensive molecular characterization of gastric adenocarcinoma. Nature.

[5] Kandoth C, Schultz N, Cherniack AD, Akbani R, Liu Y, Shen H, Robertson AG, Pashtan I, Shen R, Benz CC, Yau C, Laird PW, Ding L, Cancer Genome Atlas Research Network (2013). Integrated genomic characterization of endometrial carcinoma. Nature.

[6] (2017). Comprehensive and Integrative Genomic Characterization of Hepatocellular Carcinoma. Cell.

[7] Sawey ET, Chanrion M, Cai C, Wu G, Zhang J, Zender L, Zhao A, Busuttil RW, Yee H, Stein L, French DM, Finn RS, Lowe SW (2011). Identification of a therapeutic strategy targeting amplified FGF19 in liver cancer by Oncogenomic screening. Cancer Cell.

[8] Rahib L, Smith BD, Aizenberg R, Rosenzweig AB, Fleshman JM, Matrisian LM (2014). Projecting cancer incidence and deaths to 2030: the unexpected burden of thyroid, liver, and pancreas cancers in the United States. Cancer Res.

[9] Dhanasekaran R, Bandoh S, Roberts LR (2016). Molecular pathogenesis of hepatocellular carcinoma and impact of therapeutic advances. F1000. Res.

[10] Llovet JM, Ricci S, Mazzaferro V, Hilgard P, Gane E, Blanc JF, de Oliveira AC, Santoro A, Raoul JL, Forner A, Schwartz M, Porta C, Zeuzem S, SHARP Investigators Study Group (2008). Sorafenib in advanced hepatocellular carcinoma. N Engl J Med.

[11] Thillai K, Srikandarajah K, Ross P (2017). Regorafenib as treatment for patients with advanced hepatocellular cancer. Future oncology.

[12] Llovet JM, Hernandez-Gea V (2014). Hepatocellular carcinoma: reasons for phase III failure and novel perspectives on trial design. Clin Cancer Res.

[13] Guichard C, Amaddeo G, Imbeaud S, Ladeiro Y, Pelletier L, Maad IB, Calderaro J, Bioulac-Sage P, Letexier M, Degos F, Clément B, Balabaud C, Chevet E (2012). Integrated analysis of somatic mutations and focal copy-number changes identifies key genes and pathways in hepatocellular carcinoma. Nat Genet.

[14] Pilati C, Letouzé E, Nault JC, Imbeaud S, Boulai A, Calderaro J, Poussin K, Franconi A, Couchy G, Morcrette G, Mallet M, Taouji S, Balabaud C (2014). Genomic profiling of hepatocellular adenomas reveals recurrent FRK-activating mutations and the mechanisms of malignant transformation. Cancer Cell.

[15] Hoshida Y, Nijman SM, Kobayashi M, Chan JA, Brunet JP, Chiang DY, Villanueva A, Newell P, Ikeda K, Hashimoto M, Watanabe G, Gabriel S, Friedman SL (2009). Integrative transcriptome analysis reveals common molecular subclasses of human hepatocellular carcinoma. Cancer Res.

[16] Mokkapati S, Niopek K, Huang L, Cunniff KJ, Ruteshouser EC, deCaestecker M, Finegold MJ, Huff V (2014). β-catenin activation in a novel liver progenitor cell type is sufficient to cause hepatocellular carcinoma and hepatoblastoma. Cancer Res.

[17] Colnot S, Decaens T, Niwa-Kawakita M, Godard C, Hamard G, Kahn A, Giovannini M, Perret C (2004). Liver-targeted disruption of Apc in mice activates beta-catenin signaling and leads to hepatocellular carcinomas. Proc Natl Acad Sci USA.

[18] Tward AD, Jones KD, Yant S, Cheung ST, Fan ST, Chen X, Kay MA, Wang R, Bishop JM (2007). Distinct pathways of genomic progression to benign and malignant tumors of the liver. Proc Natl Acad Sci USA.

[19] Stauffer JK, Scarzello AJ, Andersen JB, De Kluyver RL, Back TC, Weiss JM, Thorgeirsson SS, Wiltrout RH (2011). Coactivation of AKT and β-catenin in mice rapidly induces formation of lipogenic liver tumors. Cancer Res.

[20] Wagenaar TR, Zabludoff S, Ahn SM, Allerson C, Arlt H, Baffa R, Cao H, Davis S, Garcia-Echeverria C, Gaur R, Huang SM, Jiang L, Kim D Anti-miR-21 Suppresses Hepatocellular Carcinoma Growth via Broad Transcriptional Network Deregulation.

[21] Ahn SM, Jang SJ, Shim JH, Kim D, Hong SM, Sung CO, Baek D, Haq F, Ansari AA, Lee SY, Chun SM, Choi S, Choi HJ (2014). Genomic portrait of resectable hepatocellular carcinomas: implications of RB1 and FGF19 aberrations for patient stratification. Hepatology.

[22] Aberle H, Bauer A, Stappert J, Kispert A, Kemler R (1997). beta-catenin is a target for the ubiquitin-proteasome pathway. EMBO J.

[23] Barretina J, Caponigro G, Stransky N, Venkatesan K, Margolin AA, Kim S, Wilson CJ, Lehár J, Kryukov GV, Sonkin D, Reddy A, Liu M, Murray L (2012). The Cancer Cell Line Encyclopedia enables predictive modelling of anticancer drug sensitivity. Nature.

[24] Gao C, Xiao G, Hu J (2014). Regulation of Wnt/β-catenin signaling by posttranslational modifications. Cell Biosci.

[25] Gu Q, Zhang B, Sun H, Xu Q, Tan Y, Wang G, Luo Q, Xu W, Yang S, Li J, Fu J, Chen L, Yuan S (2015). Genomic characterization of a large panel of patient-derived hepatocellular carcinoma xenograft tumor models for preclinical development. Oncotarget.

[26] Wong SC, Lo ES, Chan AK, Lee KC, Hsiao WL (2003). Nuclear beta catenin as a potential prognostic and diagnostic marker in patients with colorectal cancer from Hong Kong. Molecular Pathology.

[27] Nazemalhosseini Mojarad E, Kashfi SM, Mirtalebi H, Almasi S, Chaleshi V, Kishani Farahani R, Tarban P, Molaei M, Zali MR, J K Kuppen P (2015). Prognostic Significance of Nuclear β-Catenin Expression in Patients with Colorectal Cancer from Iran. Iran Red Crescent Med J.

[28] Elzagheid A, Buhmeida A, Korkeila E, Collan Y, Syrjänen K, Pyrhönen S (2008). Up-regulation of alpha-catenin is associated with increased lymph node involvement in colorectal cancer. World J Gastroenterol.

[29] Li XQ, Yang XL, Zhang G, Wu SP, Deng XB, Xiao SJ, Liu QZ, Yao KT, Xiao GH (2013). Nuclear β-catenin accumulation is associated with increased expression of Nanog protein and predicts poor prognosis of non-small cell lung cancer. J Transl Med.

[30] Chen J, Liu J, Jin R, Shen J, Liang Y, Ma R, Lin H, Liang X, Yu H, Cai X (2014). Cytoplasmic and/or nuclear expression of β-catenin correlate with poor prognosis and unfavorable clinicopathological factors in hepatocellular carcinoma: a meta-analysis. PLoS One.

[31] Jackson AL, Linsley PS (2010). Recognizing and avoiding siRNA off-target effects for target identification and therapeutic application. Nat Rev Drug Discov.

[32] Simioni C, Cani A, Martelli AM, Zauli G, Alameen AA, Ultimo S, Tabellini G, McCubrey JA, Capitani S, Neri LM (2015). The novel dual PI3K/mTOR inhibitor NVP-BGT226 displays cytotoxic activity in both normoxic and hypoxic hepatocarcinoma cells. Oncotarget.

[33] Goldstein LD, Lee J, Gnad F, Klijn C, Schaub A, Reeder J, Daemen A, Bakalarski CE, Holcomb T, Shames DS, Hartmaier RJ, Chmielecki J, Seshagiri S (2016). Recurrent Loss of NFE2L2 Exon 2 Is a Mechanism for Nrf2 Pathway Activation in Human Cancers. Cell Reports.

[34] Grossmann TN, Yeh JT, Bowman BR, Chu Q, Moellering RE, Verdine GL (2012). Inhibition of oncogenic Wnt signaling through direct targeting of β-catenin. Proc Natl Acad Sci USA.

[35] Dudek H, Wong DH, Arvan R, Shah A, Wortham K, Ying B (2014). Knockdown of beta-catenin with dicer-substrate siRNAs reduces liver tumor burden in vivo. Molecular Therapy.

[36] Chang W, Pei Y, Guidry EN, Zewge D, Parish CA, Sherer EC, DiMuzio J, Zhang H, South VJ, Strapps WR, Sepp-Lorenzino L, Colletti SL, Stanton MG (2016). Systematic chemical modifications of single stranded siRNAs significantly improved CTNNB1 mRNA silencing. Bioorg Med Chem Lett.

[37] Marshall J, Sun Y, Bangari DS, Budman E, Park H, Nietupski JB (2016). CNS-accessible Inhibitor of Glucosylceramide Synthase for Substrate Reduction Therapy of Neuronopathic Gaucher Disease. Molecular Therapy.

