# Supplementary material for: Oncogenic dependency on β-catenin in liver cancer cell lines correlates with pathway activation
Source: Oncotarget. 2017 Sep 28;8(70):114526–39. doi: 10.18632/oncotarget.21298 (PMC5777711; doi:10.18632/oncotarget.21298)
Supplement: Supplementary file 1 [file oncotarget-08-114526-s001.pdf]

Oncogenic dependency on β-catenin in liver cancer cell lines correlates with pathway activation

SUPPLEMENTARY MATERIALS

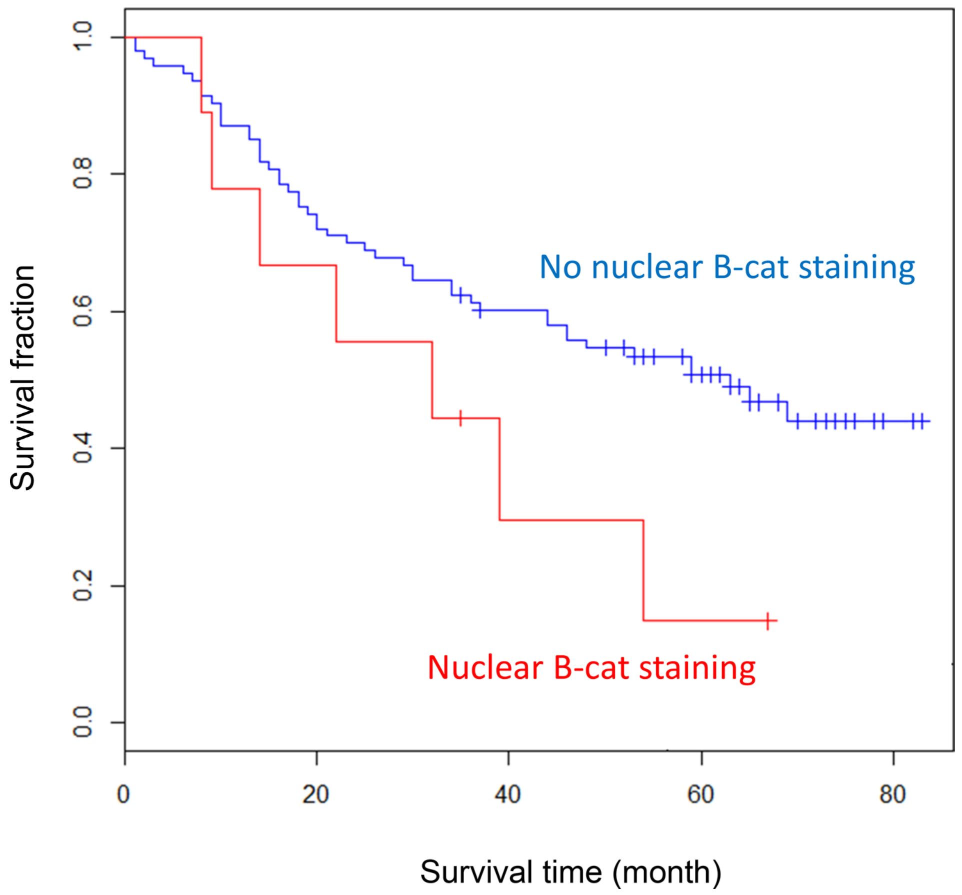

Supplementary Figure 1: Survival analysis of HCC patients with different β-catenin activation status. Kaplan-Meier curves for 9 HCC patients with exclusively nuclear B-catenin ("Bcat activation") versus 93 HCC patients without exclusively nuclear B-catenin staining ("No Bcat activation") (log-rank test $p = 0.08$).

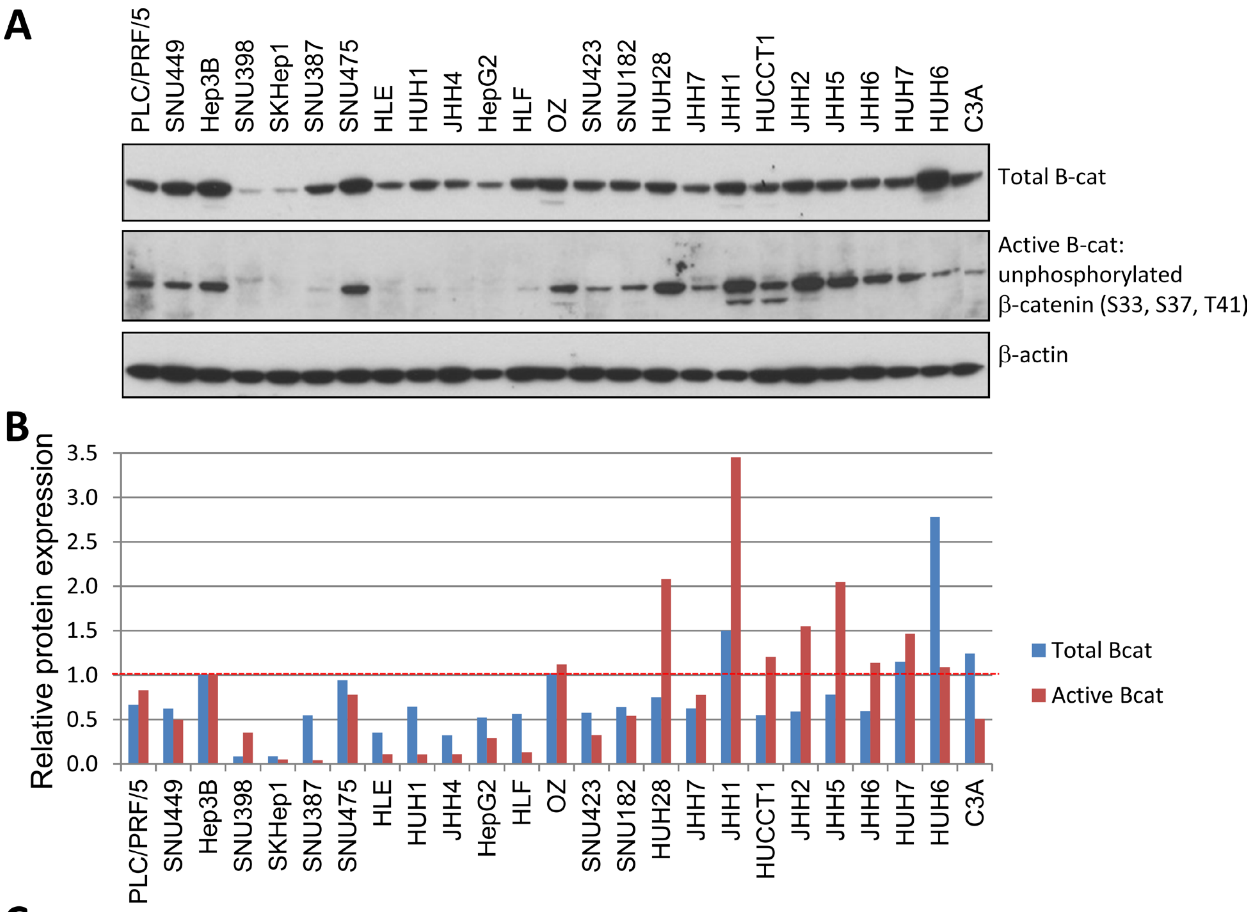

C

B-cat status	HCC cell lines	Remarks
Mutant B-cat (3)	HepG2	Heterozygous deletion of exons 3-4 of *CTNNB1* (aa 25-140)
	HUH6	*CTNNB1* mutation (G34V)
	SNU398	*CTNNB1* mutation (S37C)
High level of active B-cat (6)	Hep3B	*AXIN1* mutation
	JHH1	
	JHH2	
	JHH5	*AXIN1* mutation
	JHH6	*AXIN1* mutation
	Huh7	*FGF19* amplification (11q 13.3)
Intermediate level of active B-cat (3)	JHH7	*FGF19* amplification (11q 13.3)
	PLC/PRF/5	
	SNU449	*AXIN1* mutation
Low level of active B-cat (6)	HLE	
	HLF	
	Huh1	
	JHH4	
	SKHep1	
	SNU387	*FGF19* amplification (11q 13.3)

Supplementary Figure 2: Majority of HCC cell lines display activation of β-catenin. (**A**) Protein lysates prepared from a panel of HCC cell lines were analyzed by Western blot analysis to detect total and non-phosphorylated (active) β-catenin. β-actin was used as loading control. (**B**) Immunoblot intensity of total and active β-catenin, as well as β-actin, was quantified by ImageJ software. Total and active β-catenin were normalized to β-actin and expressed relative to Hep3B cells. (**C**) 19 HCC cell lines were stratified into high, intermediate and low levels of active β-catenin based on immunoblot data. Mutational status of *CTNNB1* (β-catenin) and FGF19 are noted.

A

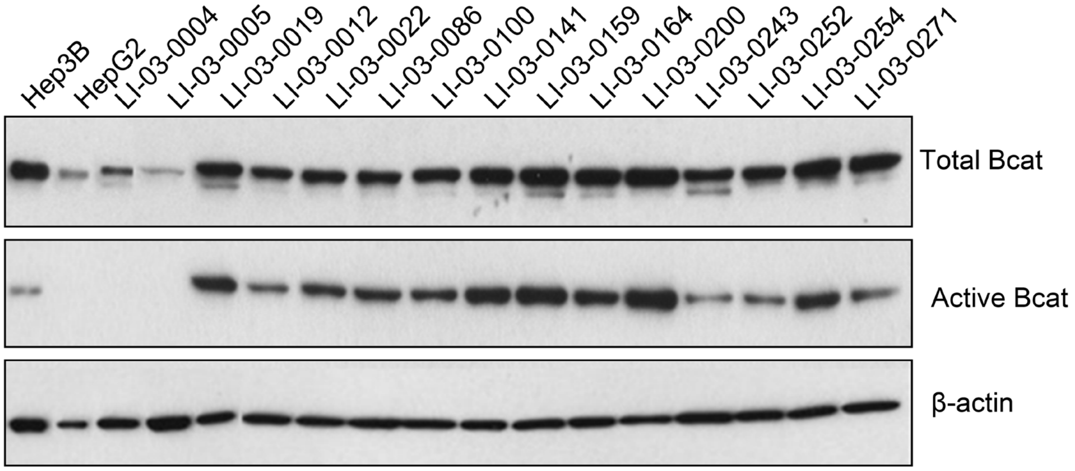

B

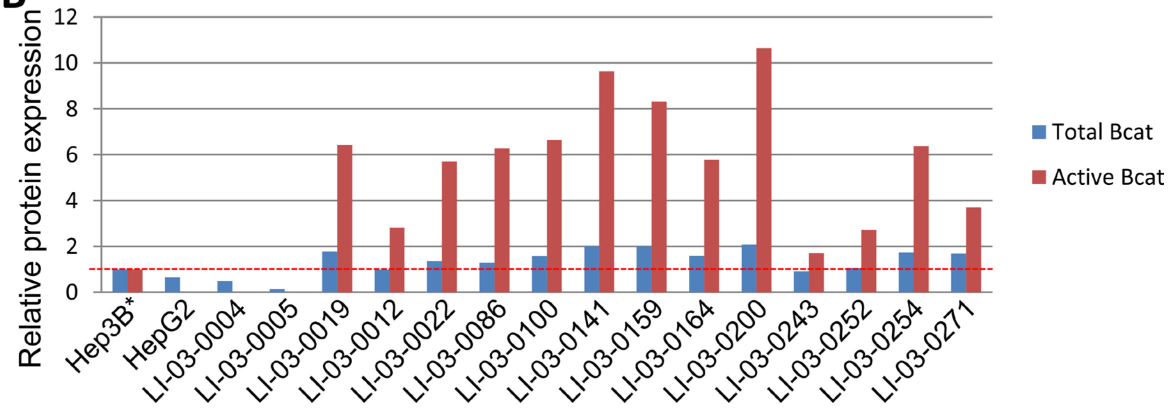

C

Sample	B-catenin		APC		AXIN1		FGF19	
	mutation	copy number	mutation	copy number	mutation	copy number	mutation	copy number
LI-03-0004	p.S37C	1.9622		2.7227		2.3816		2.0946
LI-03-0005		1.4186		2.5968		2.1143		1.1779
LI-03-0012		1.4923		1.8562		2.3728		1.7216
LI-03-0019		2.9595		2.2477		1.2601		2.1556
LI-03-0022	p.S45F	2.4582		1.8066		2.8438		2.4907
LI-03-0086		2.0052		2.624		1.585		2.1928
LI-03-0100		2.1695		2.1031		0.5989		1.4028
LI-03-0141		3.139		2.505		1.9336		5.5049
LI-03-0159		2.2243		1.406647		0.71		1.6879
LI-03-0164		1.6965		2.508237		1.004		1.8997
LI-03-0200	p.T41A	2.9611		2.3012		1.1286		1.6602
LI-03-0243		2.4261		1.6391		1.5477		2.1204
LI-03-0252		1.6756		1.6074		1.2821		1.2744
LI-03-0254		1.3825		2.435914		1.6652		1.5158
LI-03-0271		2.729		2.4418		0.9152		2.0286

Supplementary Figure 3: Majority of patient-derived xenografts (PDX) display activation of β-catenin. (**A**) Protein lysates prepared from a panel of HCC PDX or Hep3B and HepG2 cell lines were analyzed by Western blot analysis to detect total and non-phosphorylated (active) β-catenin. β-actin was used as loading control. (**B**) Immunoblot intensity of total and active β-catenin, as well as β-actin, was quantified by ImageJ software. Total and active β-catenin were normalized to β-actin and expressed relative to Hep3B cells. (**C**) Summary of genetic alterations in *CTNNB1* (β-catenin), *APC, AXIN1* and *FGF19* in HCC PDX.

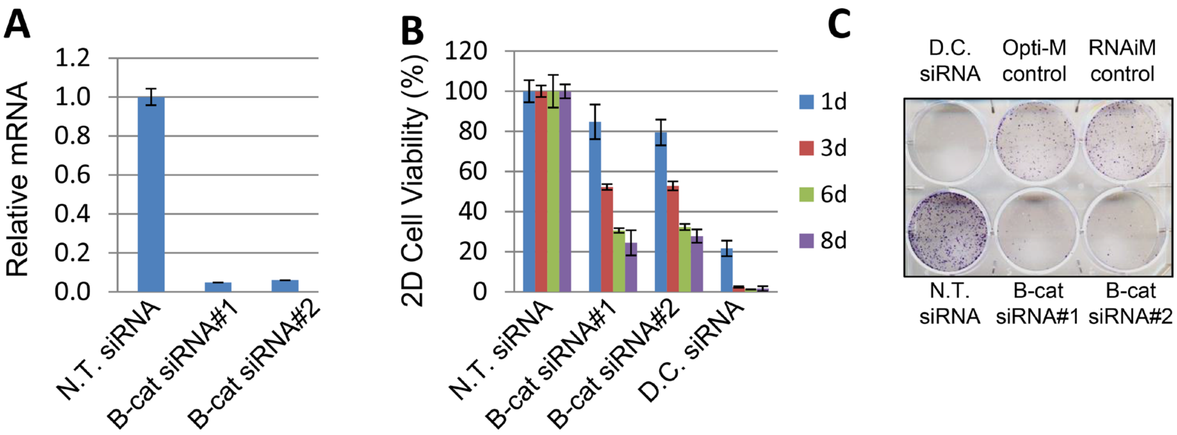

Supplementary Figure 4: Silencing of β-catenin in HepG2 leads to cell growth inhibition. (**A**) β-catenin knockdown by two independent siRNAs compared to the N.T. siRNA in HepG2 cells. (**B**) β-catenin knockdown by two independent siRNAs inhibits the viability of HepG2 cells. (**C**) Representative 2D colony formation assay of HepG2 cells. N.T. siRNA stands for non-target siRNA. D.C. siRNA stands for death control siRNA.

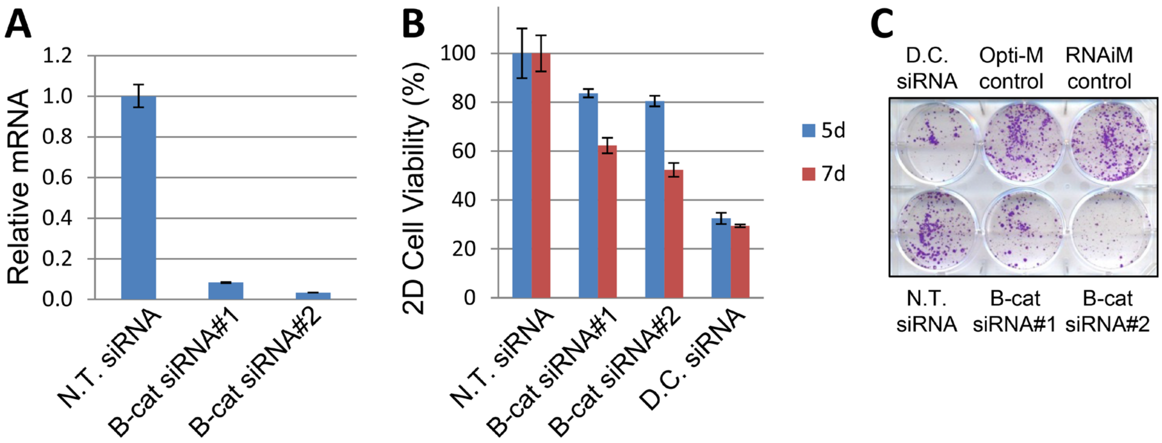

Supplementary Figure 5: Silencing of β-catenin in SNU398 leads to cell growth inhibition. (**A**) β-catenin knockdown by two independent siRNAs compared to the N.T. siRNA in SNU398 cells. (**B**) β-catenin knockdown by two independent siRNAs inhibits the viability of SNU398 cells. (**C**) Representative 2D colony formation assay of SNU398 cells.

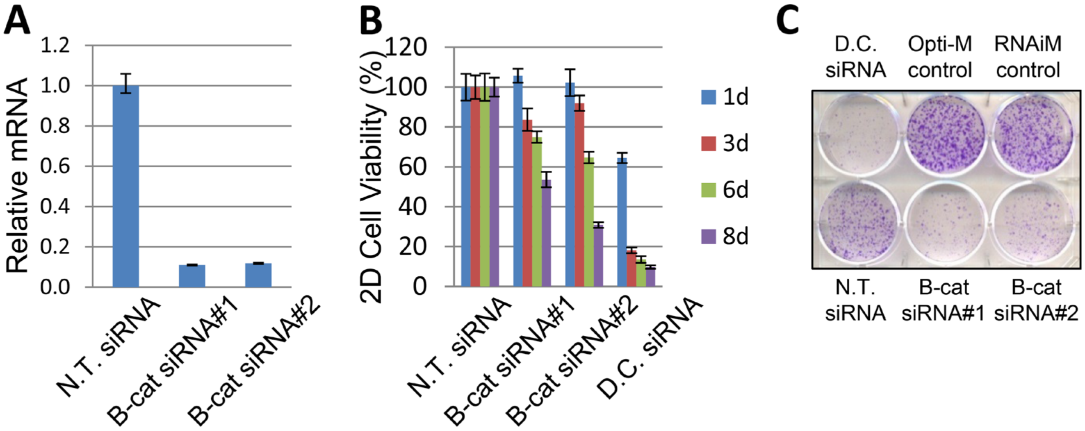

Supplementary Figure 6: Silencing of β-catenin in Hep3B leads to cell growth inhibition. (**A**) β-catenin knockdown by two independent siRNAs compared to the N.T. siRNA in Hep3B cells. (**B**) β-catenin knockdown by two independent siRNAs inhibits the viability of Hep3B cells. (**C**) Representative 2D colony formation assay of Hep3B cells.

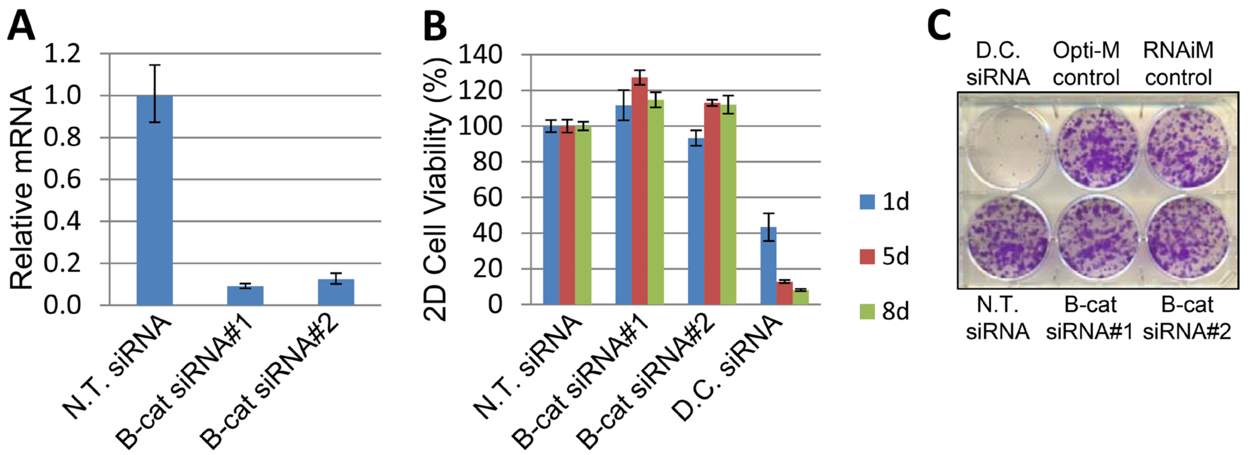

Supplementary Figure 7: Silencing of β-catenin in JHH1 does not lead to cell growth inhibition. (**A**) β-catenin knockdown by two independent siRNAs compared to the N.T. siRNA in JHH1 cells. (**B**) β-catenin knockdown by two independent siRNAs does not inhibit the viability of JHH1 cells. (**C**) Representative 2D colony formation assay of JHH1 cells.

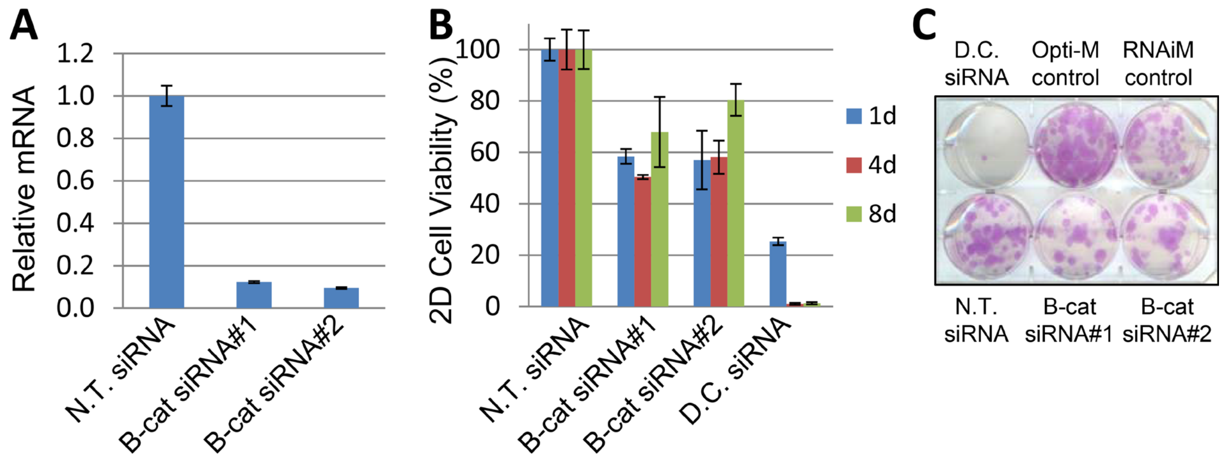

Supplementary Figure 8: Silencing of β-catenin in JHH2 leads to cell growth inhibition. (**A**) β-catenin knockdown by two independent siRNAs compared to the N.T. siRNA in JHH1 cells. (**B**) β-catenin knockdown by two independent siRNAs inhibits the viability of JHH2 cells. (**C**) Representative 2D colony formation assay of JHH1 cells.

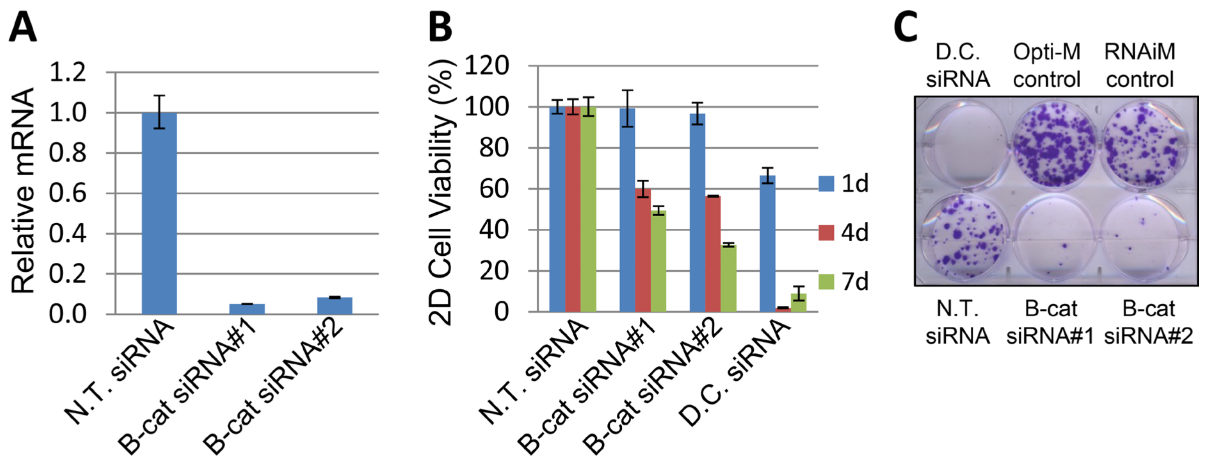

Supplementary Figure 9: Silencing of β-catenin in JHH5 leads to cell growth inhibition. (**A**) β-catenin knockdown by two independent siRNAs compared to the N.T. siRNA in JHH5 cells. (**B**) β-catenin knockdown by two independent siRNAs inhibits the viability of JHH5 cells. (**C**) Representative 2D colony formation assay of JHH5 cells.

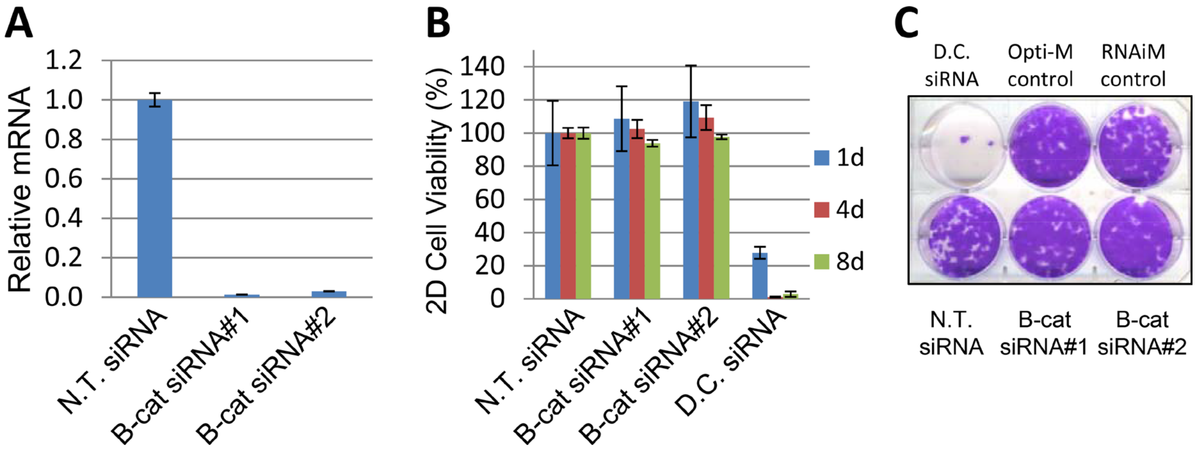

Supplementary Figure 10: Silencing of β-catenin in JHH6 does not lead to cell growth inhibition. (**A**) β-catenin knockdown by two independent siRNAs compared to the N.T. siRNA in JHH6 cells. (**B**) β-catenin knockdown by two independent siRNAs does not inhibit the viability of JHH6 cells. (**C**) Representative 2D colony formation assay of JHH6 cells.

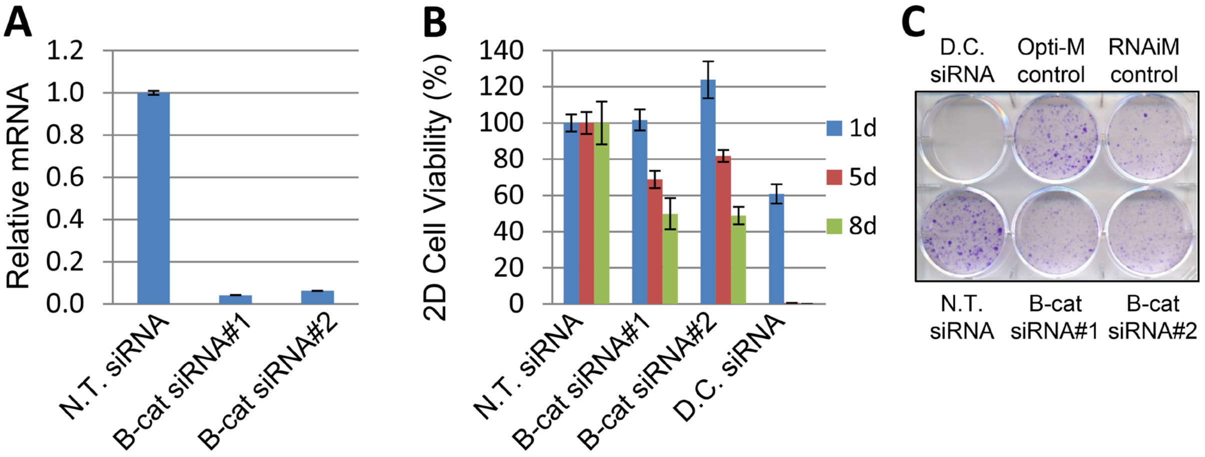

Supplementary Figure 11: Silencing of β-catenin in HUH7 leads to cell growth inhibition. (**A**) β-catenin knockdown by two independent siRNAs compared to the N.T. siRNA in HUH7 cells. (**B**) β-catenin knockdown by two independent siRNAs inhibits the viability of HUH7 cells. (**C**) Representative 2D colony formation assay of HUH7 cells.

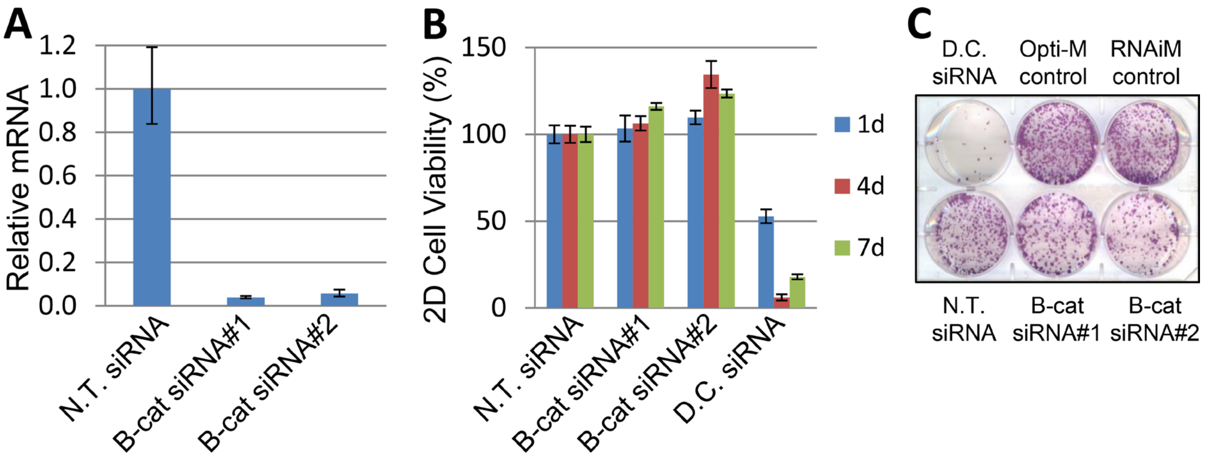

Supplementary Figure 12: Silencing of β-catenin in JHH7 does not lead to cell growth inhibition. (**A**) β-catenin knockdown by two independent siRNAs compared to the N.T. siRNA in JHH7 cells. (**B**) β-catenin knockdown by two independent siRNAs does not inhibit the viability of JHH7 cells. (**C**) Representative 2D colony formation assay of JHH7 cells.

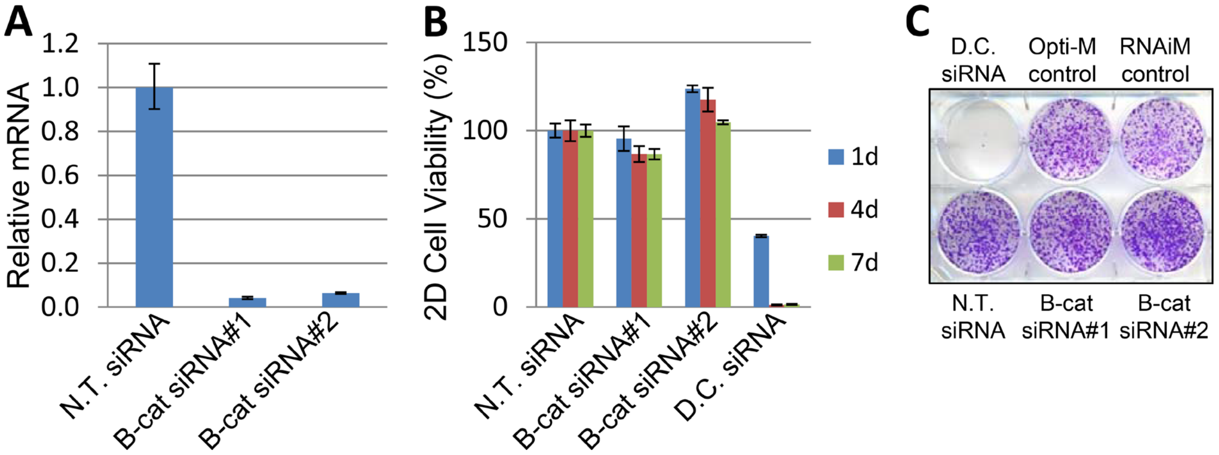

Supplementary Figure 13: Silencing of β-catenin in PLC/PRF/5 does not lead to cell growth inhibition. (**A**) β-catenin knockdown by two independent siRNAs compared to the N.T. siRNA in PLC/PRF/5 cells. (**B**) β-catenin knockdown by two independent siRNAs does not inhibit the viability of PLC/PRF/5 cells. (**C**) Representative 2D colony formation assay of PLC/PRF/5 cells.

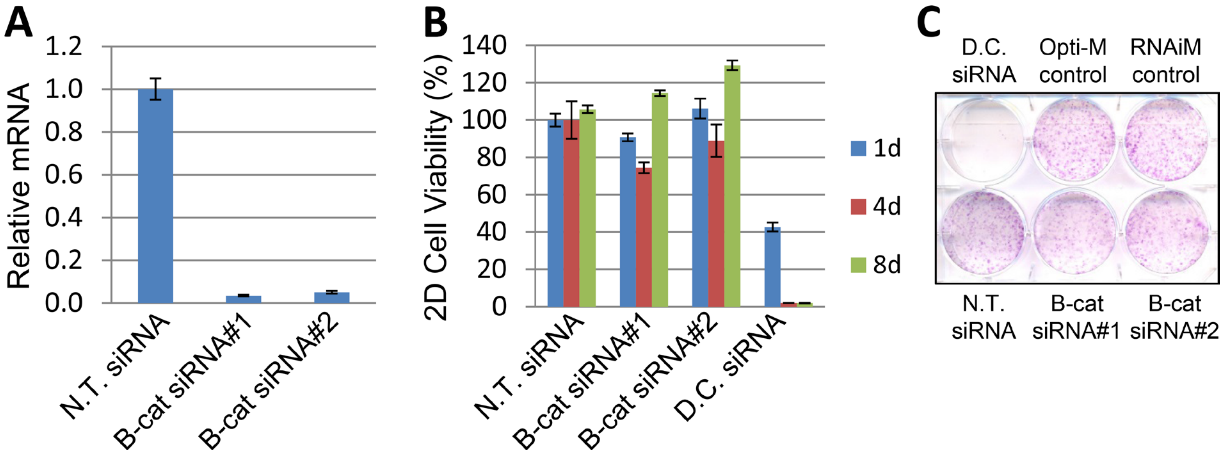

Supplementary Figure 14: Silencing of β-catenin in SNU449 does not lead to cell growth inhibition. (**A**) β-catenin knockdown by two independent siRNAs compared to the N.T. siRNA in SNU449 cells. (**B**) β-catenin knockdown by two independent siRNAs does not inhibit the viability of SNU449 cells. (**C**) Representative 2D colony formation assay of SNU449 cells.

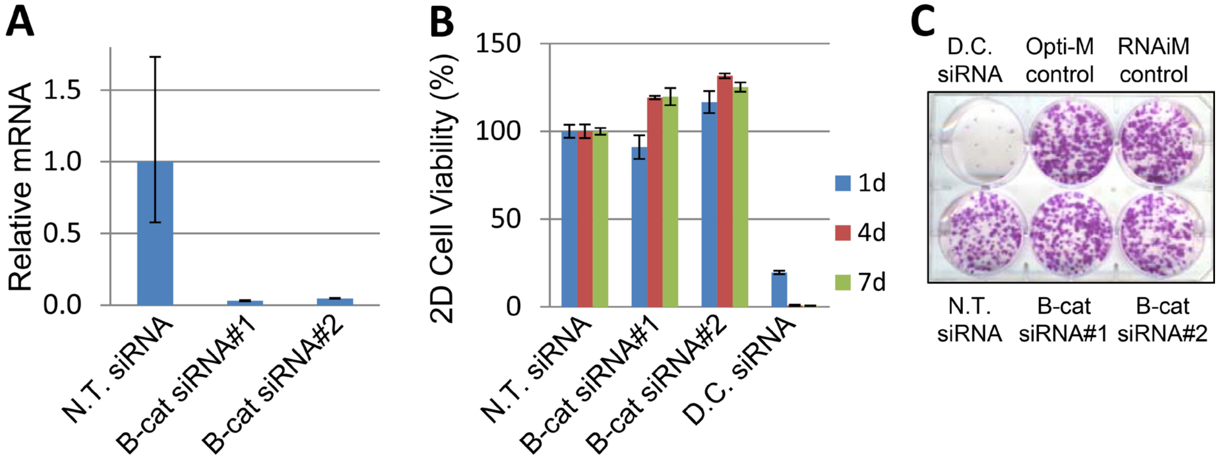

Supplementary Figure 15: Silencing of β-catenin in HLE does not lead to cell growth inhibition. (**A**) β-catenin knockdown by two independent siRNAs compared to the N.T. siRNA in HLE cells. (**B**) β-catenin knockdown by two independent siRNAs does not inhibit the viability of HLE cells. (**C**) Representative 2D colony formation assay of HLE cells.

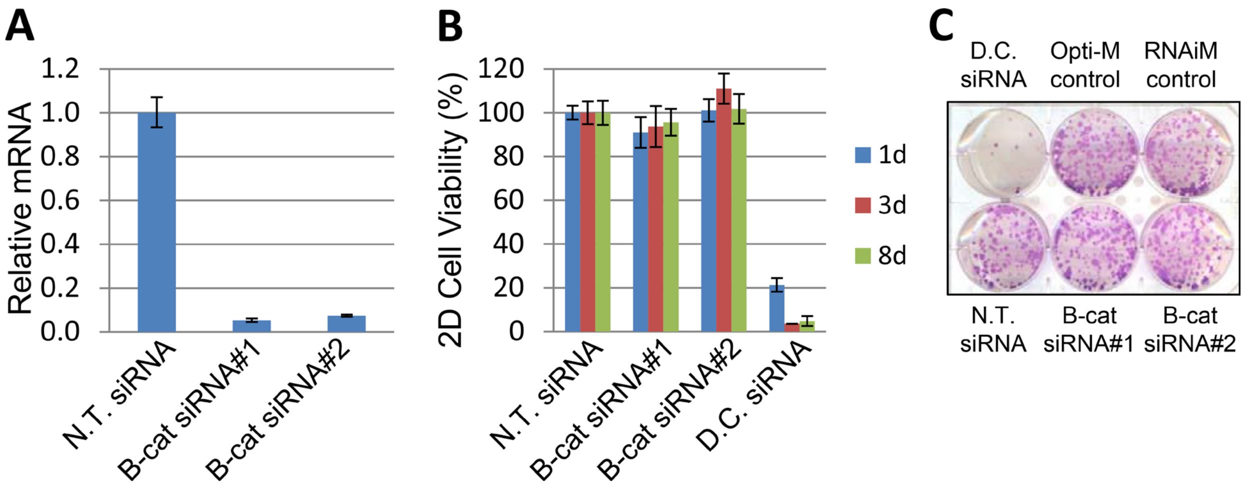

Supplementary Figure 16: Silencing of β-catenin in HLF does not lead to cell growth inhibition. (**A**) β-catenin knockdown by two independent siRNAs compared to the N.T. siRNA in HLF cells. (**B**) β-catenin knockdown by two independent siRNAs does not inhibit the viability of HLF cells. (**C**) Representative 2D colony formation assay of HLF cells.

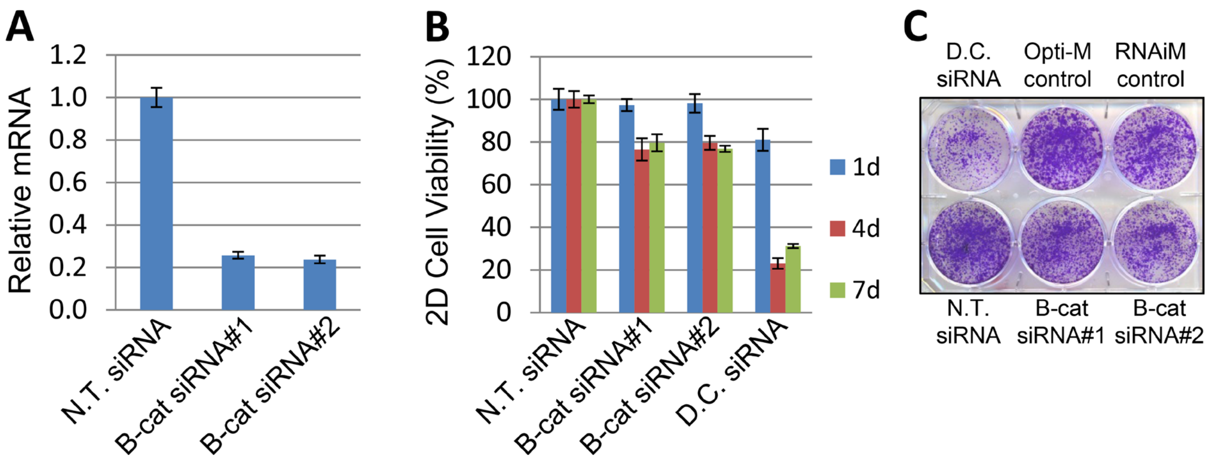

Supplementary Figure 17: Silencing of β-catenin in HUH1 does not lead to cell growth inhibition. (**A**) β-catenin knockdown by two independent siRNAs compared to the N.T. siRNA in HUH1 cells. (**B**) β-catenin knockdown by two independent siRNAs does not inhibit the viability of HUH1 cells. (**C**) Representative 2D colony formation assay of HUH1 cells.

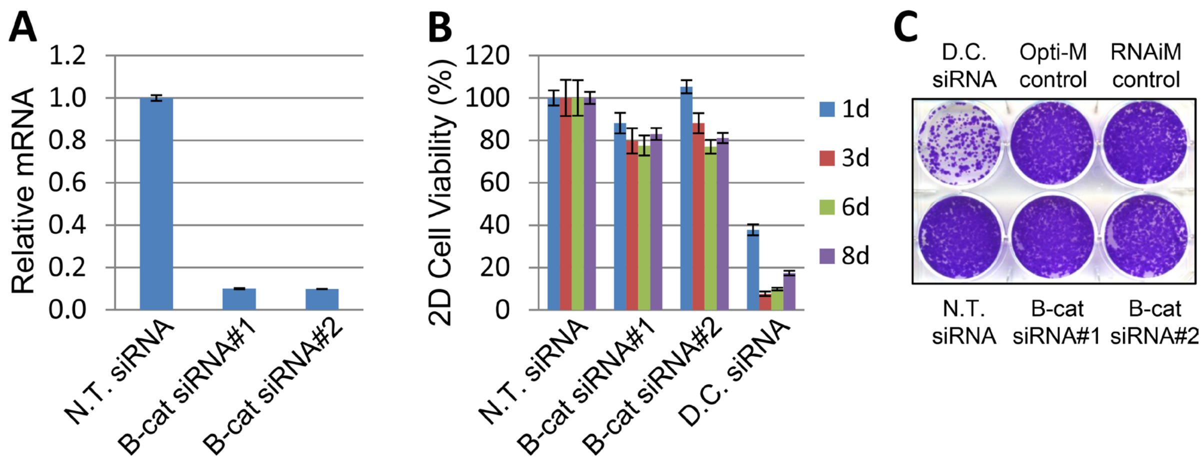

Supplementary Figure 18: Silencing of β-catenin in JHH4 does not lead to cell growth inhibition. (**A**) β-catenin knockdown by two independent siRNAs compared to the N.T. siRNA in JHH4 cells. (**B**) β-catenin knockdown by two independent siRNAs does not inhibit the viability of JHH4 cells. (**C**) Representative 2D colony formation assay of JHH4 cells.

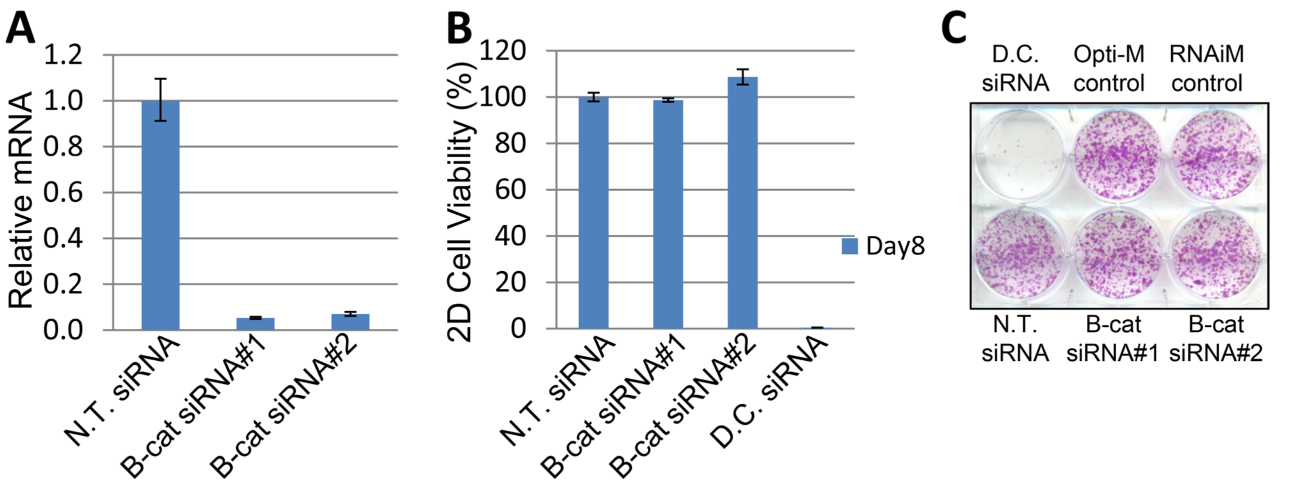

Supplementary Figure 19: Silencing of β-catenin in SKHEP1 does not lead to cell growth inhibition. (**A**) β-catenin knockdown by two independent siRNAs compared to the N.T. siRNA in SKHEP1 cells. (**B**) β-catenin knockdown by two independent siRNAs did not inhibit the viability of SKHEP1 cells. (**C**) Representative 2D colony formation assay of SKHEP1 cells.

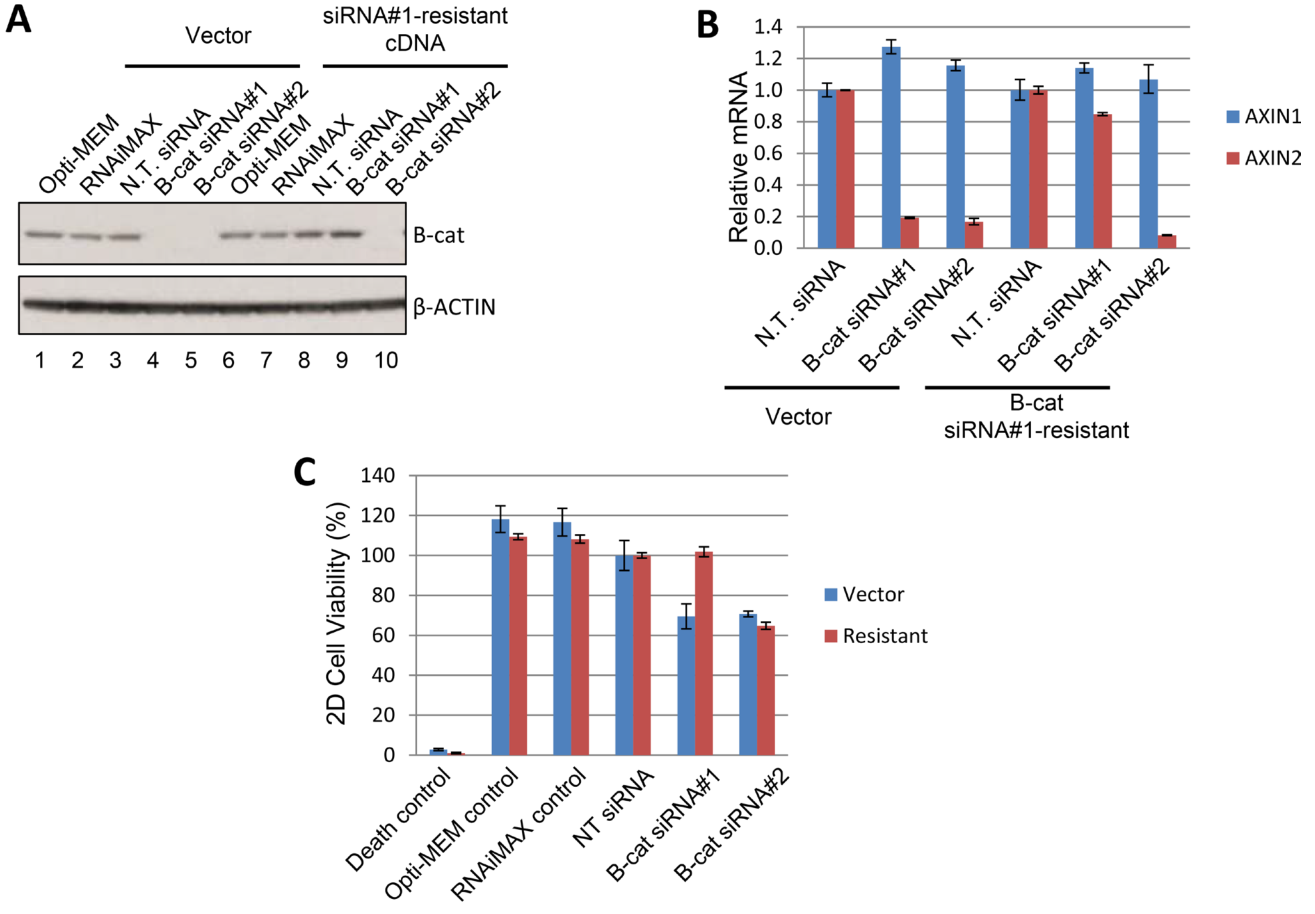

Supplementary Figure 20: Phenotypic effects of β-catenin siRNA are on-target in Hep3B cells. (**A**) Exogenous expression of siRNA#1-resistant β-catenin cDNA restored the β-catenin protein levels upon endogenous β-catenin knockdown by siRNA#1, but not siRNA#2, in Hep3B cells. The empty vector control did not restore β-catenin protein levels upon endogenous β-catenin knockdown by siRNA#1 and siRNA#2. (**B**) Exogenous expression of siRNA#1-resistant β-catenin cDNA restored the β-catenin PD marker *AXIN2* levels upon endogenous β-catenin knockdown by siRNA#1, but not siRNA#2. The empty vector control did not restore the β-catenin PD marker upon endogenous β-catenin knockdown by siRNA#1 and siRNA#2. AXIN1 gene as a non-specific control. (**C**) Expression of siRNA#1-resistant β-catenin restored the viability of cells after endogenous β-catenin knockdown, whereas the empty vector control did not.

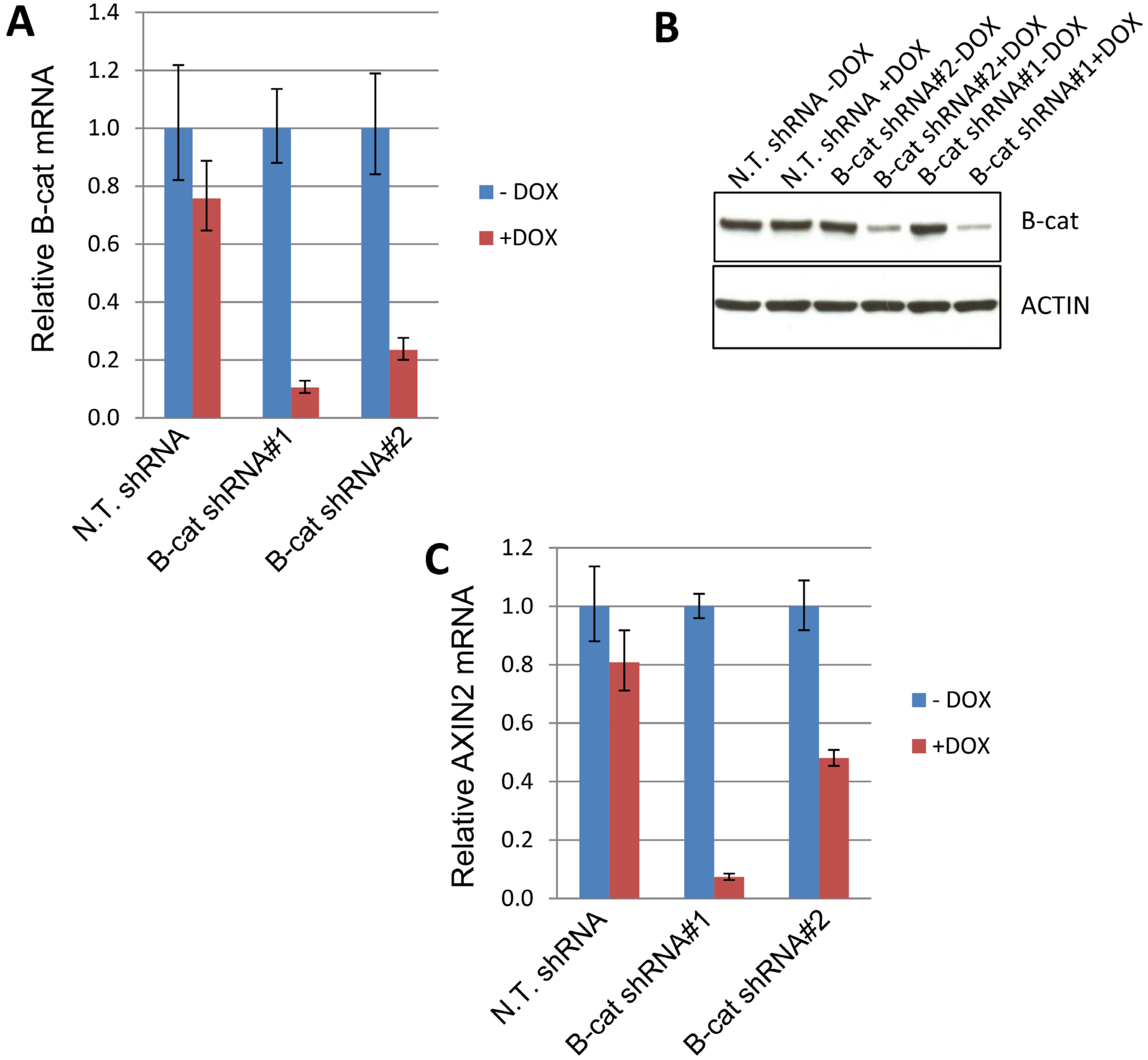

Supplementary Figure 21: Knock-down of β-catenin using inducible shRNAs. (**A–B**). Induction of shRNAs resulted in significant downregulation of β-catenin mRNA (A) and protein (B). Downstream target of β-catenin axin2 was substantially reduced in doxycycline-treated Hep3B cells (**C**).

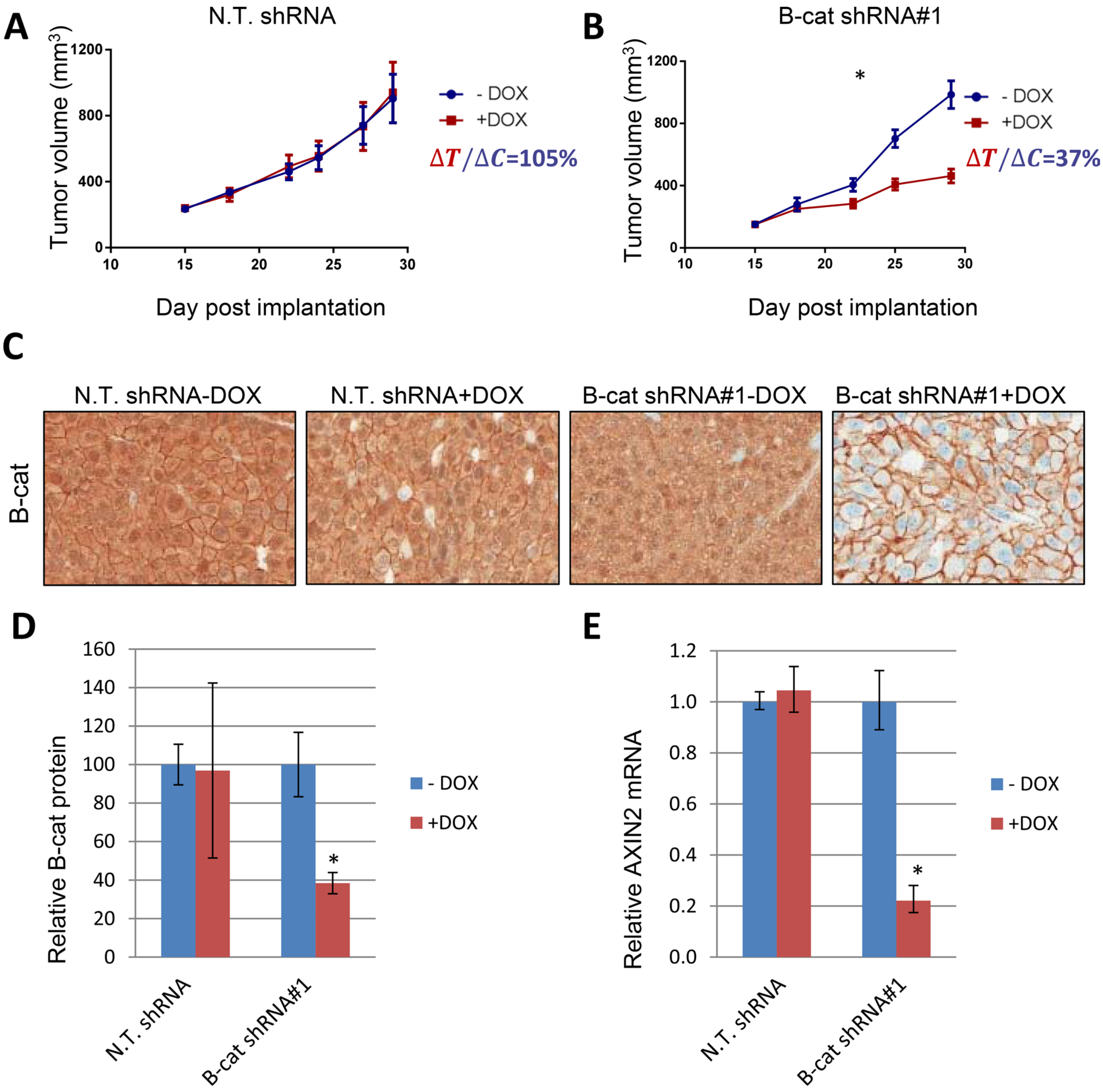

Supplementary Figure 22: Inducible knock-down of β-catenin suppresses growth of HepG2 HCC tumor xenografts. (**A–B**) Growth of non-targeting shRNA (A) or β-catenin shRNA#1 (B) HepG2 tumor xenografts in mice on doxycycline-containing (+DOX) or regular (-DOX) chow. Tumor volume was measured on the indicated days ($n = 8$ per group). Error bars represent SD of the mean. $^{*}p < 0.05$. (**C**) IHC for β-catenin in HepG2 tumors stably expressing non-targeting shRNA or β-catenin shRNA#1, in the presence or absence of doxycycline treatment. (**D**) Inducible expression of β-catenin shRNA#1, but not non-targeting control shRNA, resulted in reduced β-catenin protein levels in HepG2 tumor xenografts as measured by immunoblot analysis. (**E**) Inducible expression of β-catenin shRNA#1, but not non-targeting control shRNA, led to downregulation of β-catenin downstream target *AXIN2* in HepG2 tumors. $^{*}p < 0.05$.

Supplementary Table 1: Summary of B-catenin IHC staining in TMA1 and TMA2. See Supplementary_Table_1

Supplementary Table 2: Summary of β-catenin siRNA screen in HCC cell lines

B-cat status	HCC cell lines	Responder (R) or Non-responder (N)	Remarks
Mutant B-cat (3)	HepG2	R	Heterozygous deletion of exons 3-4 of CTNNB1 (aa 25-140)
	HUH6	R	CTNNB1 mutation (G34V)
	SNU398	R	CTNNB1 mutation (S37C)
High level of active B-cat (6)	Hep3B	R	AXIN1 mutation
	JHH1	N	
	JHH2	R	
	JHH5	R	AXIN1 mutation
	JHH6	N	AXIN1 mutation
	Huh7	R	FGF19 amplification (11q 13.3)
Intermediate level of active B-cat (3)	JHH7	N	FGF19 amplification (11q 13.3)
	PLC/PRF/5	N	
	SNU449	N	AXIN1 mutation
Low level of active B-cat (6)	HLE	N	
	HLF	N	
	Huh1	N	
	JHH4	N	
	SKHep1	N	
	SNU387	N	FGF19 amplification (11q 13.3)

Supplementary Table 3: siRNA and shRNA used in this study

siRNA reagents

siRNA	Vendor	Catalogue #	siRNA ID #	Sense (5′–3′)	Anti-Sense (5′–3′)
NT siRNA	Thermo Fisher Scientific	4390843	Negative Control No. 1 siRNA		
Death control	Qiagen	1027298	AllStars Hs Cell Death Control siRNA		
B-cat siRNA#1	Thermo Fisher Scientific	4390824	s438	CUGUUGGAUUGAU UCGAAAtt	UUUCGAAUCA AUCCAACAGta
B-cat siRNA#2	Thermo Fisher Scientific	4390824	s437	GGAUGUUCACAACCGAAUUtt	AAUUCGGUUG UGAACAUCCcg

shRNA reagents

shRNA	Clone ID	Target Sequence	shRNA ID#	Oligonucleotide Sequence
NT shRNA	CLN1640276	N/A	pLKOpuro_U6-TO_TetR_null2	ACCGTACGTTACGCGTAATGT TTCAAGAGAACGTTACGCGTA ACGTACGGT
B-cat shRNA#1	RA10657693	CTGTTGGATTG ATTCGAAATC	pLKOpuro_U6-TO_TetR_s438	CTGTTGGATTGATTCGAAATT CTCGAGGATTTCGAATCAATCC AACAGTTTTT
B-cat shRNA#2	RA10657686	GGATGTTCACA ACCGAATTGT	pLKOpuro_U6-TO_TetR_s437	GGATGTTCACAACCGAATTTTCT CGAGACAATTCGGTTGTGAACA TCCTTTTT

Supplementary Table 4: Taqman primers and probes used in this study

Gene	Vendor	Probe Dye	Catalogue #	Assay ID
GAPDH	Thermo Fisher Scientific	VIC	4326317E	Hs99999905_m1
CTNNB1	Thermo Fisher Scientific	FAM	4331182	HS00355049_m1
AXIN1	Thermo Fisher Scientific	FAM	4331182	Hs00394718_m1
AXIN2	Thermo Fisher Scientific	FAM	4331182	Hs00610344_m1
