# Supplementary material for: Oncogenic dependency on β-catenin in liver cancer cell lines correlates with pathway activation
Source: Oncotarget. 2017 Sep 28;8(70):114526–39. doi: 10.18632/oncotarget.21298 (PMC5777711; doi:10.18632/oncotarget.21298)
Supplement: Supplementary file 2 [file oncotarget-08-114526-s002.docx]

**Supplementary Table 1: Summary of B-catenin IHC staining in TMA1 and TMA2**

**TMA1**

|  |  | |  | | |  | |  |  | | | | | | | | | | | | |  | | |  |
| --- | --- | --- | --- | --- | --- | --- | --- | --- | --- | --- | --- | --- | --- | --- | --- | --- | --- | --- | --- | --- | --- | --- | --- | --- | --- |
| **Position** | **Clinical information** | | | | | | **MEMBRANE STAINING** | | | | | | | **CYTOPLASMIC STAINING** | | | | **NUCLEAR STAINING** | | | | **CYTOPLASMIC & NUCLEAR STAINING** | |  |  |
|  | **Patient** | **Pathological classification** | | **Survival info** | **Survival Period (months)** | | **Pattern (eg, whole membrane or polarized to apical membrane etc)** | | | **1+** | **2+** | **3+** | **H-score** | **1+** | **2+** | **3+** | **H-score** | **1+** | **2+** | **3+** | **H-score** | **H-score** | **Average H-score** |  |  |
| A1 | 1 | Hepatocellular carcinoma | | death | **20** | | polarized to apical membrane | | | 10 | 20 | 0 | 50 | 10 | 0 | 0 | 10 | 0 | 0 | 0 | 0 | 10 | 20 |  |  |
| B1 | 1 | Hepatocellular carcinoma | | death | **20** | | whole membrane | | | 40 | 20 | 0 | 80 | 30 | 0 | 0 | 30 | 0 | 0 | 0 | 0 | 30 |  |  |  |
| C1 | 1 | adjacent normal liver | | death | **20** | | whole membrane | | | 100 | 0 | 0 | 100 | 10 | 0 | 0 | 10 | 0 | 0 | 0 | 0 | 10 |  |  |  |
| D1 | 2 | hepatocellular carcinoma | | death | **59** | | whole membrane | | | 90 | 10 | 0 | 110 | 0 | 0 | 0 | 0 | 0 | 0 | 0 | 0 | 0 | 0 |  |  |
| E1 | 2 | hepatocellular carcinoma | | death | **59** | | whole membrane | | | 90 | 10 | 0 | 110 | 0 | 0 | 0 | 0 | 0 | 0 | 0 | 0 | 0 |  |  |  |
| F1 | 2 | adjacent normal liver | | death | **59** | | whole membrane | | | 30 | 50 | 20 | 190 | 60 | 0 | 0 | 60 | 0 | 0 | 0 | 0 | 60 |  |  |  |
| G1 | 3 | hepatocellular carcinoma | | death | **21** | |  | | | 0 | 0 | 0 | 0 | 30 | 0 | 0 | 30 | 0 | 0 | 0 | 0 | 30 | 15 |  |  |
| H1 | 3 | hepatocellular carcinoma | | death | **21** | | polarized to apical membrane | | | 10 | 0 | 0 | 10 | 0 | 0 | 0 | 0 | 0 | 0 | 0 | 0 | 0 |  |  |  |
| I1 | 3 | adjacent normal liver | | death | **21** | | whole membrane | | | 30 | 70 | 0 | 170 | 30 | 0 | 0 | 30 | 0 | 0 | 0 | 0 | 30 |  |  |  |
| A2 | 4 | Hepatocellular carcinoma | | survival | **60** | | polarized to apical membrane | | | 20 | 10 | 0 | 40 | 0 | 0 | 0 | 0 | 0 | 0 | 0 | 0 | 0 | 0 |  |  |
| B2 | 4 | Hepatocellular carcinoma | | survival | **60** | | whole membrane | | | 20 | 20 | 10 | 90 | 0 | 0 | 0 | 0 | 0 | 0 | 0 | 0 | 0 |  |  |  |
| C2 | 4 | adjacent normal liver | | survival | **60** | | whole membrane | | | 80 | 20 | 0 | 120 | 10 | 0 | 0 | 10 | 0 | 0 | 0 | 0 | 10 |  |  |  |
| D2 | 5 | hepatocellular carcinoma | | death | **14** | | whole membrane | | | 35 | 5 | 0 | 45 | 5 | 0 | 0 | 5 | 0 | 0 | 0 | 0 | 5 | 82.5 |  |  |
| E2 | 5 | hepatocellular carcinoma | | death | **14** | | whole membrane | | | 100 | 0 | 0 | 100 | 20 | 5 | 0 | 30 | 40 | 30 | 10 | 130 | 160 |  |  |  |
| F2 | 5 | adjacent normal liver | | death | **14** | | whole membrane | | | 45 | 50 | 5 | 160 | 90 | 0 | 0 | 90 | 0 | 0 | 0 | 0 | 90 |  |  |  |
| G2 | 6 | hepatocellular carcinoma | | death | **20** | | polarized to apical membrane | | | 30 | 30 | 0 | 90 | 0 | 0 | 0 | 0 | 0 | 0 | 0 | 0 | 0 | 5 |  |  |
| H2 | 6 | hepatocellular carcinoma | | death | **20** | | polarized to apical membrane | | | 80 | 20 | 0 | 120 | 10 | 0 | 0 | 10 | 0 | 0 | 0 | 0 | 10 |  |  |  |
| I2 | 6 | adjacent normal liver | | death | **20** | | whole membrane | | | 10 | 0 | 0 | 10 | 0 | 0 | 0 | 0 | 0 | 0 | 0 | 0 | 0 |  |  |  |
| A3 | 7 | Hepatocellular carcinoma | | death | **23** | | polarized to apical membrane | | | 55 | 40 | 5 | 150 | 40 | 0 | 0 | 40 | 0 | 0 | 0 | 0 | 40 | 35 |  |  |
| B3 | 7 | Hepatocellular carcinoma | | death | **23** | | whole membrane | | | 20 | 40 | 40 | 220 | 10 | 10 | 0 | 30 | 0 | 0 | 0 | 0 | 30 |  |  |  |
| C3 | 7 | adjacent normal liver | | death | **23** | | whole membrane | | | 85 | 15 | 0 | 115 | 20 | 0 | 0 | 20 | 0 | 0 | 0 | 0 | 20 |  |  |  |
| D3 | 8 | hepatocellular carcinoma | | survival | **75** | | whole membrane | | | 30 | 1 | 0 | 32 | 5 | 1 | 0 | 7 | 0 | 0 | 0 | 0 | 7 | 18.5 |  |  |
| E3 | 8 | hepatocellular carcinoma | | survival | **75** | | whole membrane | | | 90 | 10 | 0 | 110 | 30 | 0 | 0 | 30 | 0 | 0 | 0 | 0 | 30 |  |  |  |
| F3 | 8 | adjacent normal liver | | survival | **75** | | whole membrane | | | 40 | 40 | 20 | 180 | 100 | 0 | 0 | 100 | 0 | 0 | 0 | 0 | 100 |  |  |  |
| G3 | 9 | hepatocellular carcinoma | | survival | **68** | | whole membrane | | | 0 | 60 | 40 | 240 | 30 | 10 | 0 | 50 | 0 | 0 | 0 | 0 | 50 | 40 |  |  |
| H3 | 9 | hepatocellular carcinoma | | survival | **68** | | whole membrane | | | 20 | 60 | 20 | 200 | 30 | 0 | 0 | 30 | 0 | 0 | 0 | 0 | 30 |  |  |  |
| I3 | 9 | adjacent normal liver | | survival | **68** | | whole membrane | | | 10 | 90 | 0 | 190 | 10 | 0 | 0 | 10 | 0 | 0 | 0 | 0 | 10 |  |  |  |
| A4 | 10 | Hepatocellular carcinoma | | survival | **53** | | polarized to apical membrane | | | 40 | 30 | 30 | 190 | 80 | 20 | 0 | 120 | 0 | 0 | 0 | 0 | 120 | 85 |  |  |
| B4 | 10 | Hepatocellular carcinoma | | survival | **53** | | whole membrane | | | 40 | 30 | 30 | 190 | 30 | 10 | 0 | 50 | 0 | 0 | 0 | 0 | 50 |  |  |  |
| C4 | 10 | adjacent normal liver | | survival | **53** | | whole membrane | | | 70 | 30 | 0 | 130 | 10 | 0 | 0 | 10 | 0 | 0 | 0 | 0 | 10 |  |  |  |
| D4 | 11 | hepatocellular carcinoma | | survival | **72** | | whole membrane | | | 30 | 30 | 0 | 90 | 30 | 0 | 0 | 30 | 0 | 0 | 0 | 0 | 30 | 40 |  |  |
| E4 | 11 | hepatocellular carcinoma | | survival | **72** | | whole membrane | | | 20 | 80 | 0 | 180 | 50 | 0 | 0 | 50 | 0 | 0 | 0 | 0 | 50 |  |  |  |
| F4 | 11 | adjacent normal liver | | survival | **72** | | whole membrane | | | 80 | 20 | 0 | 120 | 50 | 0 | 0 | 50 | 0 | 0 | 0 | 0 | 50 |  |  |  |
| G4 | 12 | hepatocellular carcinoma | | death | **18** | | whole membrane | | | 15 | 80 | 5 | 190 | 0 | 20 | 0 | 40 | 0 | 0 | 0 | 0 | 40 | 85 |  |  |
| H4 | 12 | hepatocellular carcinoma | | death | **18** | | whole membrane | | | 60 | 40 | 0 | 140 | 70 | 30 | 0 | 130 | 0 | 0 | 0 | 0 | 130 |  |  |  |
| I4 | 12 | adjacent normal liver | | death | **18** | | whole membrane | | | 70 | 30 | 0 | 130 | 0 | 0 | 0 | 0 | 0 | 0 | 0 | 0 | 0 |  |  |  |
| A5 | 13 | Hepatocellular carcinoma | | survival | **52** | | whole membrane | | | 0 | 90 | 10 | 210 | 50 | 10 | 0 | 70 | 0 | 0 | 0 | 0 | 70 | 80 |  |  |
| B5 | 13 | Hepatocellular carcinoma | | survival | **52** | | whole membrane | | | 0 | 60 | 40 | 240 | 70 | 10 | 0 | 90 | 0 | 0 | 0 | 0 | 90 |  |  |  |
| C5 | 13 | adjacent normal liver | | survival | **52** | | whole membrane | | | 70 | 20 | 10 | 140 | 0 | 10 | 0 | 20 | 0 | 0 | 0 | 0 | 20 |  |  |  |
| D5 | 14 | hepatocellular carcinoma | | death | **34** | | whole membrane | | | 20 | 75 | 5 | 185 | 30 | 10 | 0 | 50 | 0 | 0 | 0 | 0 | 50 | 55 |  |  |
| E5 | 14 | hepatocellular carcinoma | | death | **34** | | whole membrane | | | 35 | 60 | 5 | 170 | 60 | 0 | 0 | 60 | 0 | 0 | 0 | 0 | 60 |  |  |  |
| F5 | 14 | adjacent normal liver | | death | **34** | | whole membrane | | | 70 | 0 | 0 | 70 | 30 | 0 | 0 | 30 | 0 | 0 | 0 | 0 | 30 |  |  |  |
| G5 | 15 | hepatocellular carcinoma | | death | **32** | | whole membrane | | | 0 | 40 | 60 | 260 | 80 | 20 | 0 | 120 | 0 | 0 | 0 | 0 | 120 | 135 |  |  |
| H5 | 15 | hepatocellular carcinoma | | death | **32** | | whole membrane | | | 60 | 40 | 0 | 140 | 70 | 10 | 0 | 90 | 40 | 10 | 0 | 60 | 150 |  |  |  |
| I5 | 15 | adjacent normal liver | | death | **32** | | whole membrane | | | 10 | 90 | 0 | 190 | 0 | 0 | 0 | 0 | 0 | 0 | 0 | 0 | 0 |  |  |  |
| A6 | 16 | hepatocellular carcinoma | | death | **7** | | polarized to apical membrane | | | 60 | 20 | 0 | 100 | 80 | 0 | 0 | 80 | 0 | 0 | 0 | 0 | 80 | 65 |  |  |
| B6 | 16 | hepatocellular carcinoma | | death | **7** | | polarized /whole membrane | | | 30 | 30 | 0 | 90 | 50 | 0 | 0 | 50 | 0 | 0 | 0 | 0 | 50 |  |  |  |
| C6 | 16 | adjacent normal liver | | death | **7** | | whole membrane | | | 90 | 10 | 0 | 110 | 20 | 0 | 0 | 20 | 0 | 0 | 0 | 0 | 20 |  |  |  |
| D6 | 17 | hepatocellular carcinoma | | survival | **82** | | polarized to apical membrane | | | 30 | 0 | 0 | 30 | 20 | 0 | 0 | 20 | 0 | 0 | 0 | 0 | 20 | 25 |  |  |
| E6 | 17 | hepatocellular carcinoma | | survival | **82** | | polarized /whole membrane | | | 40 | 20 | 0 | 80 | 30 | 0 | 0 | 30 | 0 | 0 | 0 | 0 | 30 |  |  |  |
| F6 | 17 | adjacent normal liver | | survival | **82** | | whole membrane | | | 100 | 0 | 0 | 100 | 10 | 0 | 0 | 10 | 0 | 0 | 0 | 0 | 10 |  |  |  |
| G6 | 18 | hepatocellular carcinoma | | death | **48** | | whole membrane | | | 65 | 30 | 5 | 140 | 50 | 0 | 0 | 50 | 0 | 0 | 0 | 0 | 50 | 40 |  |  |
| H6 | 18 | hepatocellular carcinoma | | death | **48** | | whole membrane | | | 65 | 30 | 5 | 140 | 30 | 0 | 0 | 30 | 0 | 0 | 0 | 0 | 30 |  |  |  |
| I6 | 18 | adjacent normal liver | | death | **48** | | whole membrane | | | 60 | 40 | 0 | 140 | 30 | 0 | 0 | 30 | 0 | 0 | 0 | 0 | 30 |  |  |  |
| A7 | 19 | hepatocellular carcinoma | | survival | **79** | | whole membrane | | | 10 | 80 | 10 | 200 | 90 | 10 | 0 | 110 | 0 | 0 | 0 | 0 | 110 | 85 |  |  |
| B7 | 19 | hepatocellular carcinoma | | survival | **79** | | whole membrane | | | 60 | 40 | 0 | 140 | 60 | 0 | 0 | 60 | 0 | 0 | 0 | 0 | 60 |  |  |  |
| C7 | 19 | adjacent normal liver | | survival | **79** | | whole membrane | | | 40 | 50 | 10 | 170 | 20 | 0 | 0 | 20 | 0 | 0 | 0 | 0 | 20 |  |  |  |
| D7 | 20 | hepatocellular carcinoma | | survival | **79** | | whole membrane | | | 20 | 75 | 5 | 185 | 70 | 10 | 0 | 90 | 0 | 0 | 0 | 0 | 90 | 95 |  |  |
| E7 | 20 | hepatocellular carcinoma | | survival | **79** | | whole membrane | | | 60 | 40 | 0 | 140 | 100 | 0 | 0 | 100 | 0 | 0 | 0 | 0 | 100 |  |  |  |
| F7 | 20 | adjacent normal liver | | survival | **79** | | whole membrane | | | 15 | 80 | 5 | 190 | 30 | 0 | 0 | 30 | 0 | 0 | 0 | 0 | 30 |  |  |  |
| G7 | 21 | hepatocellular carcinoma | | death | **13** | | whole membrane | | | 70 | 20 | 10 | 140 | 90 | 0 | 0 | 90 | 0 | 0 | 0 | 0 | 90 | 65 |  |  |
| H7 | 21 | hepatocellular carcinoma | | death | **13** | | whole membrane | | | 70 | 20 | 0 | 110 | 40 | 0 | 0 | 40 | 0 | 0 | 0 | 0 | 40 |  |  |  |
| I7 | 21 | adjacent normal liver | | death | **13** | | whole membrane | | | 30 | 70 | 0 | 170 | 20 | 0 | 0 | 20 | 0 | 0 | 0 | 0 | 20 |  |  |  |
| A8 | 22 | hepatocellular carcinoma | | survival | **76** | | whole membrane | | | 10 | 70 | 20 | 210 | 100 | 0 | 0 | 100 | 0 | 0 | 0 | 0 | 100 | 95 |  |  |
| B8 | 22 | hepatocellular carcinoma | | survival | **76** | | whole membrane | | | 10 | 60 | 30 | 220 | 70 | 10 | 0 | 90 | 0 | 0 | 0 | 0 | 90 |  |  |  |
| C8 | 22 | adjacent normal liver | | survival | **76** | | whole membrane | | | 50 | 40 | 10 | 160 | 0 | 0 | 0 | 0 | 0 | 0 | 0 | 0 | 0 |  |  |  |
| D8 | 23 | hepatocellular carcinoma | | death | **44** | | whole membrane | | | 100 | 0 | 0 | 100 | 20 | 0 | 0 | 20 | 0 | 0 | 0 | 0 | 20 | 15 |  |  |
| E8 | 23 | hepatocellular carcinoma | | death | **44** | | whole membrane | | | 70 | 30 | 0 | 130 | 10 | 0 | 0 | 10 | 0 | 0 | 0 | 0 | 10 |  |  |  |
| F8 | 23 | adjacent normal liver | | death | **44** | | whole membrane | | | 20 | 60 | 20 | 200 | 40 | 0 | 0 | 40 | 0 | 0 | 0 | 0 | 40 |  |  |  |
| G8 | 24 | hepatocellular carcinoma | | survival | **67** | | whole membrane | | | 60 | 40 | 0 | 140 | 50 | 30 | 0 | 110 | 5 | 1 | 0 | 7 | 117 | 208.5 |  |  |
| H8 | 24 | hepatocellular carcinoma | | survival | **67** | | whole membrane | | | 100 | 0 | 0 | 100 | 30 | 70 | 0 | 170 | 30 | 20 | 20 | 130 | 300 |  |  |  |
| I8 | 24 | adjacent normal liver | | survival | **67** | | whole membrane | | | 70 | 30 | 0 | 130 | 0 | 0 | 0 | 0 | 0 | 0 | 0 | 0 | 0 |  |  |  |
| A9 | 25 | hepatocellular carcinoma | | death | **44** | | whole membrane | | | 50 | 40 | 10 | 160 | 50 | 0 | 0 | 50 | 0 | 0 | 0 | 0 | 50 | 75 |  |  |
| B9 | 25 | hepatocellular carcinoma | | death | **44** | | whole membrane | | | 30 | 60 | 10 | 180 | 100 | 0 | 0 | 100 | 0 | 0 | 0 | 0 | 100 |  |  |  |
| C9 | 25 | adjacent normal liver | | death | **44** | | whole membrane | | | 100 | 0 | 0 | 100 | 0 | 0 | 0 | 0 | 0 | 0 | 0 | 0 | 0 |  |  |  |
| D9 | 26 | hepatocellular carcinoma | | survival | **78** | | whole membrane | | | 20 | 60 | 20 | 200 | 40 | 5 | 0 | 50 | 0 | 0 | 0 | 0 | 50 | 75 |  |  |
| E9 | 26 | hepatocellular carcinoma | | survival | **78** | | whole membrane | | | 0 | 5 | 5 | 25 | 80 | 10 | 0 | 100 | 0 | 0 | 0 | 0 | 100 |  |  |  |
| F9 | 26 | adjacent normal liver | | survival | **78** | | polarized to apical membrane | | | 10 | 0 | 0 | 10 | 0 | 0 | 0 | 0 | 0 | 0 | 0 | 0 | 0 |  |  |  |
| G9 | 27 | hepatocellular carcinoma | | survival | **66** | | whole membrane | | | 15 | 80 | 5 | 190 | 90 | 0 | 0 | 90 | 0 | 0 | 0 | 0 | 90 | 85 |  |  |
| H9 | 27 | hepatocellular carcinoma | | survival | **66** | | whole membrane | | | 0 | 70 | 30 | 230 | 80 | 0 | 0 | 80 | 0 | 0 | 0 | 0 | 80 |  |  |  |
| I9 | 27 | adjacent normal liver | | survival | **66** | | whole membrane | | | 20 | 80 | 0 | 180 | 0 | 0 | 0 | 0 | 0 | 0 | 0 | 0 | 0 |  |  |  |
| A10 | 28 | hepatocellular carcinoma | | death | **14** | | whole membrane | | | 60 | 10 | 0 | 80 | 30 | 0 | 0 | 30 | 0 | 0 | 0 | 0 | 30 | 30 |  |  |
| B10 | 28 | hepatocellular carcinoma | | death | **14** | | polarized to apical membrane | | | 60 | 20 | 0 | 100 | 30 | 0 | 0 | 30 | 0 | 0 | 0 | 0 | 30 |  |  |  |
| C10 | 28 | adjacent normal liver | | death | **14** | | whole membrane | | | 60 | 30 | 10 | 150 | 0 | 0 | 0 | 0 | 0 | 0 | 0 | 0 | 0 |  |  |  |
| D10 | 29 | hepatocellular carcinoma | | survival | **78** | | whole membrane | | | 59 | 40 | 1 | 142 | 10 | 0 | 0 | 10 | 0 | 0 | 0 | 0 | 10 | 15 |  |  |
| E10 | 29 | hepatocellular carcinoma | | survival | **78** | | whole membrane | | | 70 | 30 | 0 | 130 | 20 | 0 | 0 | 20 | 0 | 0 | 0 | 0 | 20 |  |  |  |
| F10 | 29 | adjacent normal liver | | survival | **78** | | whole membrane | | | 0 | 80 | 20 | 220 | 0 | 10 | 0 | 20 | 0 | 0 | 0 | 0 | 20 |  |  |  |
| G10 | 30 | hepatocellular carcinoma | | death | **9** | | whole membrane | | | 20 | 80 | 0 | 180 | 0 | 0 | 0 | 0 | 0 | 0 | 0 | 0 | 0 | 0 |  |  |
| H10 | 30 | hepatocellular carcinoma | | death | **9** | | whole membrane | | | 20 | 80 | 0 | 180 | 0 | 0 | 0 | 0 | 0 | 0 | 0 | 0 | 0 |  |  |  |
| I10 | 30 | adjacent normal liver | | death | **9** | | whole membrane | | | 0 | 100 | 0 | 200 | 10 | 0 | 0 | 10 | 0 | 0 | 0 | 0 | 10 |  |  |  |
| A11 | 31 | hepatocellular carcinoma | | survival | **35** | | whole membrane | | | 10 | 90 | 0 | 190 | 0 | 40 | 60 | 260 | 0 | 10 | 90 | 290 | 550 | 525 |  |  |
| B11 | 31 | hepatocellular carcinoma | | survival | **35** | | whole membrane | | | 0 | 100 | 0 | 200 | 0 | 70 | 30 | 230 | 0 | 30 | 70 | 270 | 500 |  |  |  |
| C11 | 31 | adjacent normal liver | | survival | **35** | | whole membrane | | | 100 | 0 | 0 | 100 | 20 | 0 | 0 | 20 | 0 | 0 | 0 | 0 | 20 |  |  |  |
| D11 | 32 | hepatocellular carcinoma | | death | **34** | | whole membrane | | | 70 | 25 | 5 | 135 | 10 | 0 | 0 | 10 | 0 | 0 | 0 | 0 | 10 | 35 |  |  |
| E11 | 32 | hepatocellular carcinoma | | death | **34** | | whole membrane | | | 80 | 20 | 0 | 120 | 60 | 0 | 0 | 60 | 0 | 0 | 0 | 0 | 60 |  |  |  |
| F11 | 32 | adjacent normal liver | | death | **34** | | whole membrane | | | 10 | 70 | 20 | 210 | 70 | 10 | 0 | 90 | 0 | 0 | 0 | 0 | 90 |  |  |  |
| G11 | 33 | hepatocellular carcinoma | | death | **37** | | whole membrane | | | 70 | 30 | 0 | 130 | 90 | 0 | 0 | 90 | 0 | 0 | 0 | 0 | 90 | 60 |  |  |
| H11 | 33 | hepatocellular carcinoma | | death | **37** | | whole membrane | | | 70 | 30 | 0 | 130 | 30 | 0 | 0 | 30 | 0 | 0 | 0 | 0 | 30 |  |  |  |
| I11 | 33 | adjacent normal liver | | death | **37** | | whole membrane | | | 15 | 80 | 5 | 190 | 10 | 0 | 0 | 10 | 0 | 0 | 0 | 0 | 10 |  |  |  |
| A12 | 34 | hepatocellular carcinoma | | survival | **75** | | polarized to apical membrane | | | 10 | 50 | 40 | 230 | 40 | 40 | 20 | 180 | 0 | 0 | 0 | 0 | 180 | 140 |  |  |
| B12 | 34 | hepatocellular carcinoma | | survival | **75** | | whole membrane | | | 20 | 50 | 30 | 210 | 100 | 0 | 0 | 100 | 0 | 0 | 0 | 0 | 100 |  |  |  |
| C12 | 34 | adjacent normal liver | | survival | **75** | | whole membrane | | | 80 | 20 | 0 | 120 | 20 | 0 | 0 | 20 | 0 | 0 | 0 | 0 | 20 |  |  |  |
| D12 | 35 | hepatocellular carcinoma | | death | **30** | | whole membrane | | | 20 | 60 | 20 | 200 | 90 | 0 | 0 | 90 | 0 | 0 | 0 | 0 | 90 | 85 |  |  |
| E12 | 35 | hepatocellular carcinoma | | death | **30** | | whole membrane | | | 50 | 50 | 0 | 150 | 80 | 0 | 0 | 80 | 0 | 0 | 0 | 0 | 80 |  |  |  |
| F12 | 35 | adjacent normal liver | | death | **30** | | whole membrane | | | 0 | 70 | 30 | 230 | 10 | 0 | 0 | 10 | 0 | 0 | 0 | 0 | 10 |  |  |  |
| G12 | 36 | hepatocellular carcinoma | | death | **25** | | whole membrane | | | 20 | 80 | 0 | 180 | 40 | 0 | 0 | 40 | 0 | 0 | 0 | 0 | 40 | 30 |  |  |
| H12 | 36 | hepatocellular carcinoma | | death | **25** | | whole membrane | | | 10 | 70 | 20 | 210 | 20 | 0 | 0 | 20 | 0 | 0 | 0 | 0 | 20 |  |  |  |
| I12 | 36 | adjacent normal liver | | death | **25** | | whole membrane | | | 10 | 80 | 10 | 200 | 10 | 0 | 0 | 10 | 0 | 0 | 0 | 0 | 10 |  |  |  |
| A13 | 37 | hepatocellular carcinoma | | survival | **75** | | whole membrane | | | 70 | 15 | 15 | 145 | 30 | 0 | 0 | 30 | 0 | 0 | 0 | 0 | 30 | 45 |  |  |
| B13 | 37 | hepatocellular carcinoma | | survival | **75** | | whole membrane | | | 50 | 20 | 10 | 120 | 60 | 0 | 0 | 60 | 0 | 0 | 0 | 0 | 60 |  |  |  |
| C13 | 37 | adjacent normal liver | | survival | **75** | | whole membrane | | | 90 | 10 | 0 | 110 | 0 | 0 | 0 | 0 | 0 | 0 | 0 | 0 | 0 |  |  |  |
| D13 | 38 | hepatocellular carcinoma | | death | **69** | | whole membrane | | | 40 | 30 | 30 | 190 | 80 | 10 | 0 | 100 | 0 | 0 | 0 | 0 | 100 | 100 |  |  |
| E13 | 38 | hepatocellular carcinoma | | death | **69** | | whole membrane | | | 50 | 45 | 5 | 155 | 100 | 0 | 0 | 100 | 0 | 0 | 0 | 0 | 100 |  |  |  |
| F13 | 38 | adjacent normal liver | | death | **69** | | whole membrane | | | 20 | 80 | 0 | 180 | 10 | 0 | 0 | 10 | 0 | 0 | 0 | 0 | 10 |  |  |  |
| G13 | 39 | hepatocellular carcinoma | | death | **1** | | polarized /whole membrane | | | 70 | 25 | 5 | 135 | 40 | 0 | 0 | 40 | 0 | 0 | 0 | 0 | 40 | 35 |  |  |
| H13 | 39 | hepatocellular carcinoma | | death | **1** | | polarized /whole membrane | | | 35 | 60 | 5 | 170 | 30 | 0 | 0 | 30 | 0 | 0 | 0 | 0 | 30 |  |  |  |
| I13 | 39 | adjacent normal liver | | death | **1** | | whole membrane | | | 30 | 50 | 20 | 190 | 10 | 10 | 0 | 30 | 0 | 0 | 0 | 0 | 30 |  |  |  |
| A14 | 40 | hepatocellular carcinoma | | death | **16** | | whole membrane | | | 30 | 70 | 0 | 170 | 10 | 0 | 0 | 10 | 0 | 0 | 0 | 0 | 10 | 15 |  |  |
| B14 | 40 | hepatocellular carcinoma | | death | **16** | | whole membrane | | | 90 | 10 | 0 | 110 | 20 | 0 | 0 | 20 | 0 | 0 | 0 | 0 | 20 |  |  |  |
| C14 | 40 | adjacent normal liver | | death | **16** | | whole membrane | | | 30 | 50 | 20 | 190 | 0 | 0 | 0 | 0 | 0 | 0 | 0 | 0 | 0 |  |  |  |
| D14 | 41 | hepatocellular carcinoma | | death | **8** | | whole membrane | | | 70 | 20 | 0 | 110 | 40 | 0 | 0 | 40 | 0 | 0 | 0 | 0 | 40 | 60 |  |  |
| E14 | 41 | hepatocellular carcinoma | | death | **8** | | whole membrane | | | 70 | 30 | 0 | 130 | 80 | 0 | 0 | 80 | 0 | 0 | 0 | 0 | 80 |  |  |  |
| F14 | 41 | adjacent normal liver | | death | **8** | | whole membrane | | | 100 | 0 | 0 | 100 | 0 | 0 | 0 | 0 | 0 | 0 | 0 | 0 | 0 |  |  |  |
| G14 | 42 | hepatocellular carcinoma | | death | **17** | | whole membrane | | | 100 | 0 | 0 | 100 | 0 | 0 | 0 | 0 | 0 | 0 | 0 | 0 | 0 | 0 |  |  |
| H14 | 42 | hepatocellular carcinoma | | death | **17** | | whole membrane | | | 100 | 0 | 0 | 100 | 0 | 0 | 0 | 0 | 0 | 0 | 0 | 0 | 0 |  |  |  |
| I14 | 42 | adjacent normal liver | | death | **17** | | whole membrane | | | 0 | 90 | 10 | 210 | 10 | 0 | 0 | 10 | 0 | 0 | 0 | 0 | 10 |  |  |  |
| A15 | 43 | hepatocellular carcinoma | | death | **30** | | whole membrane | | | 50 | 40 | 10 | 160 | 10 | 0 | 0 | 10 | 0 | 0 | 0 | 0 | 10 | 15 |  |  |
| B15 | 43 | hepatocellular carcinoma | | death | **30** | | whole membrane | | | 70 | 30 | 0 | 130 | 20 | 0 | 0 | 20 | 0 | 0 | 0 | 0 | 20 |  |  |  |
| C15 | 43 | adjacent normal liver | | death | **30** | | whole membrane | | | 80 | 20 | 0 | 120 | 20 | 0 | 0 | 20 | 0 | 0 | 0 | 0 | 20 |  |  |  |
| D15 | 44 | hepatocellular carcinoma | | death | **10** | | whole membrane | | | 50 | 40 | 10 | 160 | 40 | 0 | 0 | 40 | 0 | 0 | 0 | 0 | 40 | 30 |  |  |
| E15 | 44 | hepatocellular carcinoma | | death | **10** | | whole membrane | | | 80 | 10 | 1 | 103 | 20 | 0 | 0 | 20 | 0 | 0 | 0 | 0 | 20 |  |  |  |
| F15 | 44 | adjacent normal liver | | death | **10** | | whole membrane | | | 80 | 20 | 0 | 120 | 0 | 0 | 0 | 0 | 0 | 0 | 0 | 0 | 0 |  |  |  |
| G15 | 45 | hepatocellular carcinoma | | death | **6** | | whole membrane | | | 25 | 70 | 5 | 180 | 10 | 0 | 0 | 10 | 0 | 0 | 0 | 0 | 10 | 5 |  |  |
| H15 | 45 | hepatocellular carcinoma | | death | **6** | | whole membrane | | | 70 | 30 | 0 | 130 | 0 | 0 | 0 | 0 | 0 | 0 | 0 | 0 | 0 |  |  |  |
| I15 | 45 | adjacent normal liver | | death | **6** | | polarized /whole membrane | | | 40 | 40 | 20 | 180 | 30 | 0 | 0 | 30 | 0 | 0 | 0 | 0 | 30 |  |  |  |
| A16 | 46 | hepatocellular carcinoma | | survival | **74** | | polarized to apical membrane | | | 50 | 30 | 0 | 110 | 50 | 0 | 0 | 50 | 0 | 0 | 0 | 0 | 50 | 35 |  |  |
| B16 | 46 | hepatocellular carcinoma | | survival | **74** | | whole membrane | | | 80 | 20 | 0 | 120 | 20 | 0 | 0 | 20 | 0 | 0 | 0 | 0 | 20 |  |  |  |
| C16 | 46 | adjacent normal liver | | survival | **74** | | whole membrane | | | 100 | 0 | 0 | 100 | 0 | 0 | 0 | 0 | 0 | 0 | 0 | 0 | 0 |  |  |  |
| D16 | 47 | hepatocellular carcinoma | | death | **22** | | whole membrane | | | 70 | 10 | 5 | 105 | 80 | 10 | 0 | 100 | 30 | 20 | 5 | 85 | 185 | 202.5 |  |  |
| E16 | 47 | hepatocellular carcinoma | | death | **22** | | whole membrane | | | 100 | 0 | 0 | 100 | 80 | 20 | 0 | 120 | 10 | 30 | 10 | 100 | 220 |  |  |  |
| F16 | 47 | adjacent normal liver | | death | **22** | | whole membrane | | | 10 | 60 | 30 | 220 | 0 | 10 | 0 | 20 | 0 | 0 | 0 | 0 | 20 |  |  |  |
| G16 | 48 | hepatocellular carcinoma | | death | **15** | | whole membrane | | | 40 | 60 | 0 | 160 | 80 | 0 | 0 | 80 | 0 | 0 | 0 | 0 | 80 | 55 |  |  |
| H16 | 48 | hepatocellular carcinoma | | death | **15** | | whole membrane | | | 0 | 90 | 10 | 210 | 30 | 0 | 0 | 30 | 0 | 0 | 0 | 0 | 30 |  |  |  |
| I16 | 48 | adjacent normal liver | | death | **15** | | whole membrane | | | 70 | 30 | 0 | 130 | 10 | 0 | 0 | 10 | 0 | 0 | 0 | 0 | 10 |  |  |  |
| A17 | 49 | hepatocellular carcinoma | | survival | **74** | | whole membrane | | | 70 | 30 | 0 | 130 | 80 | 0 | 0 | 80 | 0 | 0 | 0 | 0 | 80 | 50 |  |  |
| B17 | 49 | hepatocellular carcinoma | | survival | **74** | | whole membrane | | | 70 | 30 | 0 | 130 | 20 | 0 | 0 | 20 | 0 | 0 | 0 | 0 | 20 |  |  |  |
| C17 | 49 | adjacent normal liver | | survival | **74** | | whole membrane | | | 80 | 20 | 0 | 120 | 10 | 0 | 0 | 10 | 0 | 0 | 0 | 0 | 10 |  |  |  |
| D17 | 50 | hepatocellular carcinoma | | survival | **73** | | polarized to apical membrane | | | 60 | 20 | 5 | 115 | 60 | 0 | 0 | 60 | 0 | 0 | 0 | 0 | 60 | 75 |  |  |
| E17 | 50 | hepatocellular carcinoma | | survival | **73** | | whole membrane | | | 80 | 10 | 0 | 100 | 50 | 20 | 0 | 90 | 0 | 0 | 0 | 0 | 90 |  |  |  |
| F17 | 50 | adjacent normal liver | | survival | **73** | | whole membrane | | | 100 | 0 | 0 | 100 | 0 | 0 | 0 | 0 | 0 | 0 | 0 | 0 | 0 |  |  |  |
| G17 | 51 | hepatocellular carcinoma | | survival | **65** | | whole membrane | | | 80 | 20 | 0 | 120 | 100 | 0 | 0 | 100 | 0 | 0 | 0 | 0 | 100 | 90 |  |  |
| H17 | 51 | hepatocellular carcinoma | | survival | **65** | | polarized /whole membrane | | | 20 | 50 | 20 | 180 | 80 | 0 | 0 | 80 | 0 | 0 | 0 | 0 | 80 |  |  |  |
| I17 | 51 | adjacent normal liver | | survival | **65** | | whole membrane | | | 30 | 0 | 0 | 30 | 0 | 0 | 0 | 0 | 0 | 0 | 0 | 0 | 0 |  |  |  |
|  |  |  | |  |  | |  | | |  |  |  |  |  |  |  |  |  |  |  |  |  |  |  |  |

**TMA2**

|  |  | |  | |  | |  | |  | | |  | | |  | |  | | |  |  | | |  | |  | |  | |  | |  | |  |  | |  |  |
| --- | --- | --- | --- | --- | --- | --- | --- | --- | --- | --- | --- | --- | --- | --- | --- | --- | --- | --- | --- | --- | --- | --- | --- | --- | --- | --- | --- | --- | --- | --- | --- | --- | --- | --- | --- | --- | --- | --- |
|  | **Clinical information** | | | | | | | **MEMBRANE STAINING** | | | | | | | | **CYTOPLASMIC STAINING** | | | | | | | **NUCLEAR STAINING** | | | | | | | | **CYTOPLASMIC & NUCLEAR STAINING** | | | | |  |  |  |
|  | **Patient** | **Pathological classification** | | **Survival info** | | **Survival Period (months)** | | **Pattern (eg, whole membrane or polarized to apical membrane etc)** | | **1+** | **2+** | | **3+** | **H-score** | | **1+** | | **2+** | **3+** | | | **H-score** | **1+** | | **2+** | | **3+** | | **H-score** | | **H-score** | | **Average H-score** | | |  |  |  |
| A1 | 52 | Hepatocellular carcinoma | | survival | | 52 | | whole membrane | | 50 | 30 | | 20 | 170 | | 0 | | 0 | 0 | | | 0 | 0 | | 0 | | 0 | | 0 | | 0 | | 35 | | |  |  |  |
| B1 | 52 | Hepatocellular carcinoma | | survival | | 52 | | whole membrane | | 60 | 30 | | 10 | 150 | | 70 | | 0 | 0 | | | 70 | 0 | | 0 | | 0 | | 0 | | 70 | |  |  |  |  |  |  |
| C1 | 52 | adjacent normal liver | | survival | | 52 | | whole membrane | | 100 | 0 | | 0 | 100 | | 0 | | 0 | 0 | | | 0 | 0 | | 0 | | 0 | | 0 | | 0 | |  | | |  |  |  |
| D1 | 53 | hepatocellular carcinoma | | survival | | 59 | | polarized to apical membrane | | 50 | 40 | | 10 | 160 | | 80 | | 0 | 0 | | | 80 | 0 | | 0 | | 0 | | 0 | | 80 | | 80 | | |  |  |  |
| E1 | 53 | hepatocellular carcinoma | | survival | | 59 | | whole membrane | | 50 | 40 | | 10 | 160 | | 80 | | 0 | 0 | | | 80 | 0 | | 0 | | 0 | | 0 | | 80 | |  |  |  |  |  |  |
| F1 | 53 | adjacent normal liver | | survival | | 59 | | whole membrane | | 90 | 0 | | 0 | 90 | | 0 | | 0 | 0 | | | 0 | 0 | | 0 | | 0 | | 0 | | 0 | |  | | |  |  |  |
| G1 | 54 | hepatocellular carcinoma | | survival | | 62 | | whole membrane | | 0 | 0 | | 100 | 300 | | 0 | | 0 | 100 | | | 300 | 0 | | 0 | | 0 | | 0 | | 300 | | 295 | | |  |  |  |
| H1 | 54 | hepatocellular carcinoma | | survival | | 62 | | whole membrane | | 0 | 10 | | 90 | 290 | | 0 | | 10 | 90 | | | 290 | 0 | | 0 | | 0 | | 0 | | 290 | |  |  |  |  |  |  |
| I1 | 54 | adjacent normal liver | | survival | | 62 | | whole membrane | | 60 | 40 | | 0 | 140 | | 10 | | 0 | 0 | | | 10 | 0 | | 0 | | 0 | | 0 | | 10 | |  | | |  |  |  |
| A2 | 55 | Hepatocellular carcinoma | | survival | | 37 | | polarized to apical membrane | | 30 | 50 | | 20 | 190 | | 50 | | 20 | 0 | | | 90 | 0 | | 0 | | 0 | | 0 | | 90 | | 85 | | |  |  |  |
| B2 | 55 | Hepatocellular carcinoma | | survival | | 37 | | polarized to apical membrane | | 40 | 50 | | 10 | 170 | | 60 | | 10 | 0 | | | 80 | 0 | | 0 | | 0 | | 0 | | 80 | |  |  |  |  |  |  |
| C2 | 55 | adjacent normal liver | | survival | | 37 | | whole membrane | | 80 | 0 | | 0 | 80 | | 0 | | 0 | 0 | | | 0 | 0 | | 0 | | 0 | | 0 | | 0 | |  | | |  |  |  |
| D2 | 56 | hepatocellular carcinoma | | death | | 53 | | whole membrane | | 20 | 20 | | 10 | 90 | | 10 | | 0 | 0 | | | 10 | 0 | | 0 | | 0 | | 0 | | 10 | | 10 | | |  |  |  |
| E2 | 56 | hepatocellular carcinoma | | death | | 53 | | whole membrane | | 50 | 20 | | 0 | 90 | | 10 | | 0 | 0 | | | 10 | 0 | | 0 | | 0 | | 0 | | 10 | |  |  |  |  |  |  |
| F2 | 56 | adjacent normal liver | | death | | 53 | | whole membrane | | 80 | 20 | | 0 | 120 | | 30 | | 0 | 0 | | | 30 | 0 | | 0 | | 0 | | 0 | | 30 | |  | | |  |  |  |
| G2 | 57 | hepatocellular carcinoma | | survival | | 61 | | whole membrane | | 30 | 60 | | 10 | 180 | | 40 | | 0 | 0 | | | 40 | 0 | | 0 | | 0 | | 0 | | 40 | | 45 | | |  |  |  |
| H2 | 57 | hepatocellular carcinoma | | survival | | 61 | | whole membrane | | 20 | 70 | | 10 | 190 | | 50 | | 0 | 0 | | | 50 | 0 | | 0 | | 0 | | 0 | | 50 | |  |  |  |  |  |  |
| I2 | 57 | adjacent normal liver | | survival | | 61 | | whole membrane | | 30 | 50 | | 20 | 190 | | 10 | | 10 | 0 | | | 30 | 0 | | 0 | | 0 | | 0 | | 30 | |  | | |  |  |  |
| A3 | 58 | hepatocellular carcinoma | | survival | | 83 | | whole membrane | | 70 | 30 | | 0 | 130 | | 30 | | 0 | 0 | | | 30 | 0 | | 0 | | 0 | | 0 | | 30 | | 45 | | |  |  |  |
| B3 | 58 | hepatocellular carcinoma | | survival | | 83 | | whole membrane | | 60 | 40 | | 0 | 140 | | 60 | | 0 | 0 | | | 60 | 0 | | 0 | | 0 | | 0 | | 60 | |  |  |  |  |  |  |
| C3 | 58 | adjacent normal liver | | survival | | 83 | | whole membrane | | 40 | 50 | | 10 | 170 | | 10 | | 0 | 0 | | | 10 | 0 | | 0 | | 0 | | 0 | | 10 | |  | | |  |  |  |
| D3 | 59 | hepatocellular carcinoma | | survival | | 58 | | whole membrane | | 50 | 20 | | 30 | 180 | | 30 | | 10 | 0 | | | 50 | 0 | | 0 | | 0 | | 0 | | 50 | | 65 | | |  |  |  |
| E3 | 59 | hepatocellular carcinoma | | survival | | 58 | | whole membrane | | 20 | 60 | | 20 | 200 | | 60 | | 10 | 0 | | | 80 | 0 | | 0 | | 0 | | 0 | | 80 | |  |  |  |  |  |  |
| F3 | 59 | adjacent normal liver | | survival | | 58 | | whole membrane | | 100 | 0 | | 0 | 100 | | 0 | | 0 | 0 | | | 0 | 0 | | 0 | | 0 | | 0 | | 0 | |  | | |  |  |  |
| G3 | 60 | hepatocellular carcinoma | | death | | 14 | | whole membrane | | 80 | 20 | | 0 | 120 | | 10 | | 0 | 0 | | | 10 | 0 | | 0 | | 0 | | 0 | | 10 | | 5 | | |  |  |  |
| H3 | 60 | hepatocellular carcinoma | | death | | 14 | | whole membrane | | 50 | 40 | | 0 | 130 | | 0 | | 0 | 0 | | | 0 | 0 | | 0 | | 0 | | 0 | | 0 | |  |  |  |  |  |  |
| I3 | 60 | adjacent normal liver | | death | | 14 | | whole membrane | | 60 | 40 | | 0 | 140 | | 10 | | 0 | 0 | | | 10 | 0 | | 0 | | 0 | | 0 | | 10 | |  | | |  |  |  |
| A4 | 61 | hepatocellular carcinoma | | death | | 26 | | polarized to apical membrane | | 40 | 40 | | 0 | 120 | | 40 | | 20 | 0 | | | 80 | 0 | | 0 | | 0 | | 0 | | 80 | | 65 | | |  |  |  |
| B4 | 61 | hepatocellular carcinoma | | death | | 26 | | polarized to apical membrane | | 30 | 30 | | 0 | 90 | | 50 | | 0 | 0 | | | 50 | 0 | | 0 | | 0 | | 0 | | 50 | |  |  |  |  |  |  |
| C4 | 61 | adjacent normal liver | | death | | 26 | | whole membrane | | 80 | 20 | | 0 | 120 | | 0 | | 0 | 0 | | | 0 | 0 | | 0 | | 0 | | 0 | | 0 | |  | | |  |  |  |
| D4 | 62 | hepatocellular carcinoma | | survival | | 58 | | whole membrane | | 70 | 30 | | 0 | 130 | | 20 | | 0 | 0 | | | 20 | 0 | | 0 | | 0 | | 0 | | 20 | | 10 | | |  |  |  |
| E4 | 62 | hepatocellular carcinoma | | survival | | 58 | | whole membrane | | 80 | 20 | | 0 | 120 | | 0 | | 0 | 0 | | | 0 | 0 | | 0 | | 0 | | 0 | | 0 | |  |  |  |  |  |  |
| F4 | 62 | adjacent normal liver | | survival | | 58 | | whole membrane | | 50 | 30 | | 20 | 170 | | 10 | | 0 | 0 | | | 10 | 0 | | 0 | | 0 | | 0 | | 10 | |  | | |  |  |  |
| G4 | 63 | hepatocellular carcinoma | | death | | 8 | | whole membrane | | 20 | 0 | | 0 | 20 | | 10 | | 0 | 0 | | | 10 | 0 | | 0 | | 0 | | 0 | | 10 | | 10 | | |  |  |  |
| H4 | 63 | hepatocellular carcinoma | | death | | 8 | | whole membrane | | 20 | 0 | | 0 | 20 | | 10 | | 0 | 0 | | | 10 | 0 | | 0 | | 0 | | 0 | | 10 | |  |  |  |  |  |  |
| I4 | 63 | adjacent normal liver | | death | | 8 | | whole membrane | | 40 | 60 | | 0 | 160 | | 10 | | 0 | 0 | | | 10 | 0 | | 0 | | 0 | | 0 | | 10 | |  | | |  |  |  |
| A5 | 64 | hepatocellular carcinoma | | survival | | 55 | | polarized to apical membrane | | 60 | 20 | | 0 | 100 | | 0 | | 0 | 0 | | | 0 | 0 | | 0 | | 0 | | 0 | | 0 | | 0 | | |  |  |  |
| B5 | 64 | hepatocellular carcinoma | | survival | | 55 | | polarized to apical membrane | | 50 | 30 | | 0 | 110 | | 0 | | 0 | 0 | | | 0 | 0 | | 0 | | 0 | | 0 | | 0 | |  |  |  |  |  |  |
| C5 | 64 | adjacent normal liver | | survival | | 55 | | whole membrane | | 100 | 0 | | 0 | 100 | | 0 | | 0 | 0 | | | 0 | 0 | | 0 | | 0 | | 0 | | 0 | |  | | |  |  |  |
| D5 | 65 | hepatocellular carcinoma | | death | | 19 | | whole membrane | | 40 | 50 | | 10 | 170 | | 30 | | 0 | 0 | | | 30 | 0 | | 0 | | 0 | | 0 | | 30 | | 65 | | |  |  |  |
| E5 | 65 | hepatocellular carcinoma | | death | | 19 | | whole membrane | | 30 | 60 | | 10 | 180 | | 80 | | 10 | 0 | | | 100 | 0 | | 0 | | 0 | | 0 | | 100 | |  |  |  |  |  |  |
| F5 | 65 | adjacent normal liver | | death | | 19 | | whole membrane | | 50 | 40 | | 10 | 160 | | 10 | | 0 | 0 | | | 10 | 0 | | 0 | | 0 | | 0 | | 10 | |  | | |  |  |  |
| G5 | 66 | hepatocellular carcinoma | | death | | 3 | | whole membrane | | 30 | 70 | | 0 | 170 | | 40 | | 0 | 0 | | | 40 | 0 | | 0 | | 0 | | 0 | | 40 | | 35 | | |  |  |  |
| H5 | 66 | hepatocellular carcinoma | | death | | 3 | | whole membrane | | 60 | 40 | | 0 | 140 | | 30 | | 0 | 0 | | | 30 | 0 | | 0 | | 0 | | 0 | | 30 | |  |  |  |  |  |  |
| I5 | 66 | adjacent normal liver | | death | | 3 | | whole membrane | | 20 | 0 | | 0 | 20 | | 0 | | 0 | 0 | | | 0 | 0 | | 0 | | 0 | | 0 | | 0 | |  | | |  |  |  |
| A6 | 67 | hepatocellular carcinoma | | death | | 59 | | whole membrane | | 60 | 40 | | 0 | 140 | | 10 | | 0 | 0 | | | 10 | 0 | | 0 | | 0 | | 0 | | 10 | | 10 | | |  |  |  |
| B6 | 67 | hepatocellular carcinoma | | death | | 59 | | whole membrane | | 60 | 40 | | 0 | 140 | | 10 | | 0 | 0 | | | 10 | 0 | | 0 | | 0 | | 0 | | 10 | |  |  |  |  |  |  |
| C6 | 67 | adjacent normal liver | | death | | 59 | | whole membrane | | 0 | 30 | | 70 | 270 | | 20 | | 0 | 0 | | | 20 | 0 | | 0 | | 0 | | 0 | | 20 | |  | | |  |  |  |
| D6 | 68 | hepatocellular carcinoma | | death | | 39 | | whole membrane | | 60 | 30 | | 10 | 150 | | 30 | | 20 | 10 | | | 100 | 10 | | 0 | | 0 | | 10 | | 110 | | 110 | | |  |  |  |
| E6 | 68 | hepatocellular carcinoma | | death | | 39 | | whole membrane | | 50 | 40 | | 10 | 160 | | 50 | | 30 | 0 | | | 110 | 0 | | 0 | | 0 | | 0 | | 110 | |  |  |  |  |  |  |
| F6 | 68 | adjacent normal liver | | death | | 39 | | whole membrane | | 50 | 50 | | 0 | 150 | | 10 | | 0 | 0 | | | 10 | 0 | | 0 | | 0 | | 0 | | 10 | |  | | |  |  |  |
| G6 | 69 | hepatocellular carcinoma | | survival | | 50 | | whole membrane | | 20 | 80 | | 0 | 180 | | 30 | | 60 | 0 | | | 150 | 0 | | 0 | | 0 | | 0 | | 150 | | 135 | | |  |  |  |
| H6 | 69 | hepatocellular carcinoma | | survival | | 50 | | whole membrane | | 30 | 70 | | 0 | 170 | | 80 | | 20 | 0 | | | 120 | 0 | | 0 | | 0 | | 0 | | 120 | |  |  |  |  |  |  |
| I6 | 69 | adjacent normal liver | | survival | | 50 | | whole membrane | | 40 | 40 | | 20 | 180 | | 20 | | 0 | 0 | | | 20 | 0 | | 0 | | 0 | | 0 | | 20 | |  | | |  |  |  |
| A7 | 70 | hepatocellular carcinoma | | survival | | 72 | | polarized to apical membrane | | 60 | 20 | | 0 | 100 | | 20 | | 0 | 0 | | | 20 | 0 | | 0 | | 0 | | 0 | | 20 | | 25 | | |  |  |  |
| B7 | 70 | hepatocellular carcinoma | | survival | | 72 | | whole membrane | | 50 | 30 | | 0 | 110 | | 30 | | 0 | 0 | | | 30 | 0 | | 0 | | 0 | | 0 | | 30 | |  |  |  |  |  |  |
| C7 | 70 | adjacent normal liver | | survival | | 72 | | whole membrane | | 10 | 50 | | 40 | 230 | | 20 | | 0 | 0 | | | 20 | 0 | | 0 | | 0 | | 0 | | 20 | |  | | |  |  |  |
| D7 | 71 | hepatocellular carcinoma | | survival | | 65 | | polarized to apical membrane | | 30 | 30 | | 20 | 150 | | 0 | | 0 | 0 | | | 0 | 0 | | 0 | | 0 | | 0 | | 0 | | 0 | | |  |  |  |
| E7 | 71 | hepatocellular carcinoma | | survival | | 65 | | whole membrane | | NA | NA | | NA | NA | | NA | | NA | NA | | | NA | NA | | NA | | NA | | NA | | 0 | |  |  |  |  |  |  |
| F7 | 71 | adjacent normal liver | | survival | | 65 | | whole membrane | | 60 | 30 | | 10 | 150 | | 10 | | 0 | 0 | | | 10 | 0 | | 0 | | 0 | | 0 | | 10 | |  | | |  |  |  |
| G7 | 72 | hepatocellular carcinoma | | death | | 46 | | whole membrane | | 20 | 20 | | 40 | 180 | | 60 | | 0 | 0 | | | 60 | 0 | | 0 | | 0 | | 0 | | 60 | | 40 | | |  |  |  |
| H7 | 72 | hepatocellular carcinoma | | death | | 46 | | whole membrane | | 80 | 20 | | 0 | 120 | | 20 | | 0 | 0 | | | 20 | 0 | | 0 | | 0 | | 0 | | 20 | |  |  |  |  |  |  |
| I7 | 72 | adjacent normal liver | | death | | 46 | | whole membrane | | 40 | 50 | | 10 | 170 | | 20 | | 0 | 0 | | | 20 | 0 | | 0 | | 0 | | 0 | | 20 | |  | | |  |  |  |
| A8 | 73 | hepatocellular carcinoma | | survival | | 70 | | polarized to apical membrane | | 30 | 0 | | 0 | 30 | | 0 | | 0 | 0 | | | 0 | 0 | | 0 | | 0 | | 0 | | 0 | | 5 | | |  |  |  |
| B8 | 73 | hepatocellular carcinoma | | survival | | 70 | | whole membrane | | 60 | 0 | | 0 | 60 | | 10 | | 0 | 0 | | | 10 | 0 | | 0 | | 0 | | 0 | | 10 | |  |  |  |  |  |  |
| C8 | 73 | adjacent normal liver | | survival | | 70 | | whole membrane | | 0 | 80 | | 20 | 220 | | 20 | | 0 | 0 | | | 20 | 0 | | 0 | | 0 | | 0 | | 20 | |  | | |  |  |  |
| D8 | 74 | hepatocellular carcinoma | | death | | 63 | | whole membrane | | 10 | 70 | | 20 | 210 | | 40 | | 20 | 0 | | | 80 | 0 | | 0 | | 0 | | 0 | | 80 | | 90 | | |  |  |  |
| E8 | 74 | hepatocellular carcinoma | | death | | 63 | | whole membrane | | 20 | 70 | | 10 | 190 | | 80 | | 10 | 0 | | | 100 | 0 | | 0 | | 0 | | 0 | | 100 | |  |  |  |  |  |  |
| F8 | 74 | adjacent normal liver | | death | | 63 | | whole membrane | | 40 | 50 | | 10 | 170 | | 80 | | 0 | 0 | | | 80 | 0 | | 0 | | 0 | | 0 | | 80 | |  | | |  |  |  |
| G8 | 75 | hepatocellular carcinoma | | death | | 14 | | whole membrane | | 80 | 20 | | 0 | 120 | | 30 | | 0 | 0 | | | 30 | 0 | | 0 | | 0 | | 0 | | 30 | | 25 | | |  |  |  |
| H8 | 75 | hepatocellular carcinoma | | death | | 14 | | whole membrane | | 90 | 10 | | 0 | 110 | | 20 | | 0 | 0 | | | 20 | 0 | | 0 | | 0 | | 0 | | 20 | |  |  |  |  |  |  |
| I8 | 75 | adjacent normal liver | | death | | 14 | | whole membrane | | 80 | 20 | | 0 | 120 | | 20 | | 0 | 0 | | | 20 | 0 | | 0 | | 0 | | 0 | | 20 | |  | | |  |  |  |
| A9 | 76 | hepatocellular carcinoma | | death | | 2 | | whole membrane | | 30 | 50 | | 10 | 160 | | 30 | | 10 | 0 | | | 50 | 0 | | 0 | | 0 | | 0 | | 50 | | 40 | | |  |  |  |
| B9 | 76 | hepatocellular carcinoma | | death | | 2 | | whole membrane | | 40 | 60 | | 0 | 160 | | 30 | | 0 | 0 | | | 30 | 0 | | 0 | | 0 | | 0 | | 30 | |  |  |  |  |  |  |
| C9 | 76 | adjacent normal liver | | death | | 2 | | whole membrane | | 70 | 10 | | 0 | 90 | | 0 | | 0 | 0 | | | 0 | 0 | | 0 | | 0 | | 0 | | 0 | |  | | |  |  |  |
| D9 | 77 | hepatocellular carcinoma | | death | | 65 | | whole membrane | | 70 | 30 | | 0 | 130 | | 10 | | 0 | 0 | | | 10 | 0 | | 0 | | 0 | | 0 | | 10 | | 10 | | |  |  |  |
| E9 | 77 | hepatocellular carcinoma | | death | | 65 | | whole membrane | | 70 | 20 | | 10 | 140 | | 10 | | 0 | 0 | | | 10 | 0 | | 0 | | 0 | | 0 | | 10 | |  |  |  |  |  |  |
| F9 | 77 | adjacent normal liver | | death | | 65 | | whole membrane | | 50 | 50 | | 0 | 150 | | 10 | | 0 | 0 | | | 10 | 0 | | 0 | | 0 | | 0 | | 10 | |  | | |  |  |  |
| G9 | 78 | hepatocellular carcinoma | | death | | 29 | | whole membrane | | 70 | 30 | | 0 | 130 | | 30 | | 0 | 0 | | | 30 | 0 | | 0 | | 0 | | 0 | | 30 | | 25 | | |  |  |  |
| H9 | 78 | hepatocellular carcinoma | | death | | 29 | | whole membrane | | 80 | 20 | | 0 | 120 | | 20 | | 0 | 0 | | | 20 | 0 | | 0 | | 0 | | 0 | | 20 | |  |  |  |  |  |  |
| I9 | 78 | adjacent normal liver | | death | | 29 | | whole membrane | | 20 | 50 | | 30 | 210 | | 10 | | 0 | 0 | | | 10 | 0 | | 0 | | 0 | | 0 | | 10 | |  | | |  |  |  |
| A10 | 79 | hepatocellular carcinoma | | death | | 16 | | whole membrane | | 50 | 30 | | 0 | 110 | | 0 | | 0 | 0 | | | 0 | 0 | | 0 | | 0 | | 0 | | 0 | | 10 | | |  |  |  |
| B10 | 79 | hepatocellular carcinoma | | death | | 16 | | whole membrane | | 60 | 40 | | 0 | 140 | | 20 | | 0 | 0 | | | 20 | 0 | | 0 | | 0 | | 0 | | 20 | |  |  |  |  |  |  |
| C10 | 79 | adjacent normal liver | | death | | 16 | | whole membrane | | 100 | 0 | | 0 | 100 | | 0 | | 0 | 0 | | | 0 | 0 | | 0 | | 0 | | 0 | | 0 | |  | | |  |  |  |
| D10 | 80 | hepatocellular carcinoma | | survival | | 64 | | whole membrane | | 60 | 30 | | 10 | 150 | | 10 | | 0 | 0 | | | 10 | 0 | | 0 | | 0 | | 0 | | 10 | | 30 | | |  |  |  |
| E10 | 80 | hepatocellular carcinoma | | survival | | 64 | | whole membrane | | 30 | 40 | | 20 | 170 | | 30 | | 10 | 0 | | | 50 | 0 | | 0 | | 0 | | 0 | | 50 | |  |  |  |  |  |  |
| F10 | 80 | adjacent normal liver | | survival | | 64 | | whole membrane | | 40 | 60 | | 0 | 160 | | 10 | | 0 | 0 | | | 10 | 0 | | 0 | | 0 | | 0 | | 10 | |  | | |  |  |  |
| G10 | 81 | hepatocellular carcinoma | | death | | 8 | | polarized to apical membrane | | 40 | 30 | | 0 | 100 | | 40 | | 0 | 0 | | | 40 | 40 | | 0 | | 0 | | 40 | | 80 | | 75 | | |  |  |  |
| H10 | 81 | hepatocellular carcinoma | | death | | 8 | | whole membrane | | 30 | 20 | | 0 | 70 | | 30 | | 0 | 0 | | | 30 | 20 | | 10 | | 0 | | 40 | | 70 | |  |  |  |  |  |  |
| I10 | 81 | adjacent normal liver | | death | | 8 | | whole membrane | | 0 | 60 | | 40 | 240 | | 10 | | 0 | 0 | | | 10 | 0 | | 0 | | 0 | | 0 | | 10 | |  | | |  |  |  |
| A11 | 82 | hepatocellular carcinoma | | survival | | 64 | | whole membrane | | 70 | 0 | | 0 | 70 | | 90 | | 0 | 0 | | | 90 | 0 | | 0 | | 0 | | 0 | | 90 | | 50 | | |  |  |  |
| B11 | 82 | hepatocellular carcinoma | | survival | | 64 | | whole membrane | | 20 | 0 | | 0 | 20 | | 10 | | 0 | 0 | | | 10 | 0 | | 0 | | 0 | | 0 | | 10 | |  |  |  |  |  |  |
| C11 | 82 | adjacent normal liver | | survival | | 64 | | whole membrane | | 100 | 0 | | 0 | 100 | | 0 | | 0 | 0 | | | 0 | 0 | | 0 | | 0 | | 0 | | 0 | |  | | |  |  |  |
| D11 | 83 | hepatocellular carcinoma | | survival | | 64 | | whole membrane | | 30 | 50 | | 20 | 190 | | 30 | | 10 | 0 | | | 50 | 0 | | 0 | | 0 | | 0 | | 50 | | 45 | | |  |  |  |
| E11 | 83 | hepatocellular carcinoma | | survival | | 64 | | whole membrane | | 50 | 30 | | 20 | 170 | | 20 | | 10 | 0 | | | 40 | 0 | | 0 | | 0 | | 0 | | 40 | |  |  |  |  |  |  |
| F11 | 83 | adjacent normal liver | | survival | | 64 | | whole membrane | | 60 | 40 | | 0 | 140 | | 20 | | 0 | 0 | | | 20 | 0 | | 0 | | 0 | | 0 | | 20 | |  | | |  |  |  |
| G11 | 84 | hepatocellular carcinoma | | death | | 13 | |  | | 0 | 0 | | 0 | 0 | | 0 | | 0 | 0 | | | 0 | 0 | | 0 | | 0 | | 0 | | 0 | | 0 | | |  |  |  |
| H11 | 84 | hepatocellular carcinoma | | death | | 13 | | whole membrane | | 0 | 0 | | 0 | 0 | | 0 | | 0 | 0 | | | 0 | 0 | | 0 | | 0 | | 0 | | 0 | |  |  |  |  |  |  |
| I11 | 84 | adjacent normal liver | | death | | 13 | | whole membrane | | 20 | 60 | | 20 | 200 | | 20 | | 0 | 0 | | | 20 | 0 | | 0 | | 0 | | 0 | | 20 | |  | | |  |  |  |
| A12 | 85 | hepatocellular carcinoma | | survival | | 63 | | whole membrane | | 10 | 80 | | 10 | 200 | | 80 | | 10 | 0 | | | 100 | 0 | | 0 | | 0 | | 0 | | 100 | | 90 | | |  |  |  |
| B12 | 85 | hepatocellular carcinoma | | survival | | 63 | | whole membrane | | 60 | 30 | | 10 | 150 | | 80 | | 0 | 0 | | | 80 | 0 | | 0 | | 0 | | 0 | | 80 | |  |  |  |  |  |  |
| C12 | 85 | adjacent normal liver | | survival | | 63 | | whole membrane | | 80 | 20 | | 0 | 120 | | 0 | | 0 | 0 | | | 0 | 0 | | 0 | | 0 | | 0 | | 0 | |  | | |  |  |  |
| D12 | 86 | hepatocellular carcinoma | | death | | 18 | | polarized to apical membrane | | 30 | 20 | | 40 | 190 | | 60 | | 10 | 0 | | | 80 | 0 | | 0 | | 0 | | 0 | | 80 | | 95 | | |  |  |  |
| E12 | 86 | hepatocellular carcinoma | | death | | 18 | | polarized to apical membrane | | 40 | 30 | | 0 | 100 | | 50 | | 30 | 0 | | | 110 | 0 | | 0 | | 0 | | 0 | | 110 | |  |  |  |  |  |  |
| F12 | 86 | adjacent normal liver | | death | | 18 | | whole membrane | | 80 | 20 | | 0 | 120 | | 10 | | 0 | 0 | | | 10 | 0 | | 0 | | 0 | | 0 | | 10 | |  | | |  |  |  |
| G12 | 87 | hepatocellular carcinoma | | death | | 10 | | polarized to apical membrane | | 40 | 30 | | 20 | 160 | | 80 | | 0 | 0 | | | 80 | 0 | | 0 | | 0 | | 0 | | 80 | | 125 | | |  |  |  |
| H12 | 87 | hepatocellular carcinoma | | death | | 10 | | whole membrane | | 0 | 0 | | 0 | 0 | | 30 | | 70 | 0 | | | 170 | 0 | | 0 | | 0 | | 0 | | 170 | |  |  |  |  |  |  |
| I12 | 87 | adjacent normal liver | | death | | 10 | | whole membrane | | 20 | 0 | | 0 | 20 | | 0 | | 0 | 0 | | | 0 | 0 | | 0 | | 0 | | 0 | | 0 | |  | | |  |  |  |
| A13 | 88 | hepatocellular carcinoma | | survival | | 54 | | whole membrane | | 60 | 30 | | 0 | 120 | | 30 | | 0 | 0 | | | 30 | 0 | | 0 | | 0 | | 0 | | 30 | | 30 | | |  |  |  |
| B13 | 88 | hepatocellular carcinoma | | survival | | 54 | | whole membrane | | 70 | 30 | | 0 | 130 | | 30 | | 0 | 0 | | | 30 | 0 | | 0 | | 0 | | 0 | | 30 | |  |  |  |  |  |  |
| C13 | 88 | adjacent normal liver | | survival | | 54 | | whole membrane | | 0 | 80 | | 20 | 220 | | 20 | | 0 | 0 | | | 20 | 0 | | 0 | | 0 | | 0 | | 20 | |  | | |  |  |  |
| D13 | 89 | hepatocellular carcinoma | | survival | | 62 | | whole membrane | | 50 | 40 | | 10 | 160 | | 20 | | 10 | 0 | | | 40 | 0 | | 0 | | 0 | | 0 | | 40 | | 45 | | |  |  |  |
| E13 | 89 | hepatocellular carcinoma | | survival | | 62 | | whole membrane | | 60 | 30 | | 10 | 150 | | 30 | | 10 | 0 | | | 50 | 0 | | 0 | | 0 | | 0 | | 50 | |  |  |  |  |  |  |
| F13 | 89 | adjacent normal liver | | survival | | 62 | | whole membrane | | 20 | 50 | | 30 | 210 | | 10 | | 30 | 0 | | | 70 | 0 | | 0 | | 0 | | 0 | | 70 | |  | | |  |  |  |
| G13 | 90 | hepatocellular carcinoma | | death | | 54 | | whole membrane | | 50 | 30 | | 10 | 140 | | 30 | | 10 | 0 | | | 50 | 40 | | 0 | | 0 | | 40 | | 90 | | 120 | | |  |  |  |
| H13 | 90 | hepatocellular carcinoma | | death | | 54 | | whole membrane | | 20 | 50 | | 30 | 210 | | 50 | | 20 | 0 | | | 90 | 60 | | 0 | | 0 | | 60 | | 150 | |  |  |  |  |  |  |
| I13 | 90 | adjacent normal liver | | death | | 54 | | whole membrane | | 0 | 20 | | 80 | 280 | | 0 | | 20 | 0 | | | 40 | 0 | | 0 | | 0 | | 0 | | 40 | |  | | |  |  |  |
| A14 | 91 | hepatocellular carcinoma | | survival | | 63 | | whole membrane | | 80 | 20 | | 0 | 120 | | 10 | | 0 | 0 | | | 10 | 0 | | 0 | | 0 | | 0 | | 10 | | 5 | | |  |  |  |
| B14 | 91 | hepatocellular carcinoma | | survival | | 63 | | whole membrane | | 50 | 30 | | 0 | 110 | | 0 | | 0 | 0 | | | 0 | 0 | | 0 | | 0 | | 0 | | 0 | |  |  |  |  |  |  |
| C14 | 91 | adjacent normal liver | | survival | | 63 | | whole membrane | | 100 | 0 | | 0 | 100 | | 10 | | 0 | 0 | | | 10 | 0 | | 0 | | 0 | | 0 | | 10 | |  | | |  |  |  |
| D14 | 92 | hepatocellular carcinoma | | death | | 46 | | whole membrane | | 50 | 50 | | 0 | 150 | | 0 | | 100 | 0 | | | 200 | 0 | | 0 | | 0 | | 0 | | 200 | | 185 | | |  |  |  |
| E14 | 92 | hepatocellular carcinoma | | death | | 46 | | whole membrane | | 70 | 30 | | 0 | 130 | | 40 | | 50 | 10 | | | 170 | 0 | | 0 | | 0 | | 0 | | 170 | |  |  |  |  |  |  |
| F14 | 92 | adjacent normal liver | | death | | 46 | | whole membrane | | 80 | 20 | | 0 | 120 | | 10 | | 0 | 0 | | | 10 | 0 | | 0 | | 0 | | 0 | | 10 | |  | | |  |  |  |
| G14 | 93 | hepatocellular carcinoma | | death | | 36 | | whole membrane | | 50 | 20 | | 10 | 120 | | 70 | | 10 | 0 | | | 90 | 0 | | 0 | | 0 | | 0 | | 90 | | 75 | | |  |  |  |
| H14 | 93 | hepatocellular carcinoma | | death | | 36 | | whole membrane | | 40 | 20 | | 0 | 80 | | 40 | | 10 | 0 | | | 60 | 0 | | 0 | | 0 | | 0 | | 60 | |  |  |  |  |  |  |
| I14 | 93 | adjacent normal liver | | death | | 36 | | whole membrane | | 60 | 40 | | 0 | 140 | | 0 | | 0 | 0 | | | 0 | 0 | | 0 | | 0 | | 0 | | 0 | |  | | |  |  |  |
| A15 | 94 | hepatocellular carcinoma | | survival | | 62 | | whole membrane | | 20 | 0 | | 0 | 20 | | 0 | | 0 | 0 | | | 0 | 0 | | 0 | | 0 | | 0 | | 0 | | 0 | | |  |  |  |
| B15 | 94 | hepatocellular carcinoma | | survival | | 62 | | whole membrane | | 40 | 10 | | 0 | 60 | | 0 | | 0 | 0 | | | 0 | 0 | | 0 | | 0 | | 0 | | 0 | |  |  |  |  |  |  |
| C15 | 94 | adjacent normal liver | | survival | | 62 | | whole membrane | | 50 | 40 | | 10 | 160 | | 10 | | 0 | 0 | | | 10 | 0 | | 0 | | 0 | | 0 | | 10 | |  | | |  |  |  |
| D15 | 95 | hepatocellular carcinoma | | survival | | 61 | | whole membrane | | 60 | 30 | | 10 | 150 | | 70 | | 30 | 0 | | | 130 | 0 | | 0 | | 0 | | 0 | | 130 | | 100 | | |  |  |  |
| E15 | 95 | hepatocellular carcinoma | | survival | | 61 | | whole membrane | | 30 | 70 | | 0 | 170 | | 70 | | 0 | 0 | | | 70 | 0 | | 0 | | 0 | | 0 | | 70 | |  |  |  |  |  |  |
| F15 | 95 | adjacent normal liver | | survival | | 61 | | whole membrane | | 20 | 80 | | 0 | 180 | | 20 | | 0 | 0 | | | 20 | 0 | | 0 | | 0 | | 0 | | 20 | |  | | |  |  |  |
| G15 | 96 | hepatocellular carcinoma | | death | | 1 | | polarized to apical membrane | | 40 | 20 | | 20 | 140 | | 90 | | 0 | 0 | | | 90 | 0 | | 0 | | 0 | | 0 | | 90 | | 85 | | |  |  |  |
| H15 | 96 | hepatocellular carcinoma | | death | | 1 | | polarized to apical membrane | | 40 | 30 | | 10 | 130 | | 80 | | 0 | 0 | | | 80 | 0 | | 0 | | 0 | | 0 | | 80 | |  |  |  |  |  |  |
| I15 | 96 | adjacent normal liver | | death | | 1 | | whole membrane | | 30 | 50 | | 20 | 190 | | 40 | | 0 | 0 | | | 40 | 0 | | 0 | | 0 | | 0 | | 40 | |  | | |  |  |  |
| A16 | 97 | hepatocellular carcinoma | | survival | | 61 | | polarized to apical membrane | | 40 | 20 | | 10 | 110 | | 0 | | 0 | 0 | | | 0 | 0 | | 0 | | 0 | | 0 | | 0 | | 0 | | |  |  |  |
| B16 | 97 | hepatocellular carcinoma | | survival | | 61 | | polarized to apical membrane | | 40 | 20 | | 10 | 110 | | 0 | | 0 | 0 | | | 0 | 0 | | 0 | | 0 | | 0 | | 0 | |  |  |  |  |  |  |
| C16 | 97 | adjacent normal liver | | survival | | 61 | | whole membrane | | 80 | 20 | | 0 | 120 | | 10 | | 0 | 0 | | | 10 | 0 | | 0 | | 0 | | 0 | | 10 | |  | | |  |  |  |
| D16 | 98 | hepatocellular carcinoma | | survival | | 35 | | whole membrane | | 80 | 20 | | 0 | 120 | | 10 | | 0 | 0 | | | 10 | 0 | | 0 | | 0 | | 0 | | 10 | | 20 | | |  |  |  |
| E16 | 98 | hepatocellular carcinoma | | survival | | 35 | | whole membrane | | 50 | 40 | | 10 | 160 | | 30 | | 0 | 0 | | | 30 | 0 | | 0 | | 0 | | 0 | | 30 | |  |  |  |  |  |  |
| F16 | 98 | adjacent normal liver | | survival | | 35 | | whole membrane | | 60 | 20 | | 0 | 100 | | 10 | | 0 | 0 | | | 10 | 0 | | 0 | | 0 | | 0 | | 10 | |  | | |  |  |  |
| G16 | 99 | hepatocellular carcinoma | | death | | 10 | | whole membrane | | 70 | 30 | | 0 | 130 | | 0 | | 0 | 0 | | | 0 | 0 | | 0 | | 0 | | 0 | | 0 | | 30 | | |  |  |  |
| H16 | 99 | hepatocellular carcinoma | | death | | 10 | | polarized to apical membrane | | 50 | 20 | | 10 | 120 | | 60 | | 0 | 0 | | | 60 | 0 | | 0 | | 0 | | 0 | | 60 | |  |  |  |  |  |  |
| I16 | 99 | adjacent normal liver | | death | | 10 | | whole membrane | | 20 | 60 | | 20 | 200 | | 30 | | 0 | 0 | | | 30 | 0 | | 0 | | 0 | | 0 | | 30 | |  | | |  |  |  |
| A17 | 100 | hepatocellular carcinoma | | survival | | 60 | | whole membrane | | 70 | 30 | | 0 | 130 | | 0 | | 0 | 0 | | | 0 | 0 | | 0 | | 0 | | 0 | | 0 | | 0 | | |  |  |  |
| B17 | 100 | hepatocellular carcinoma | | survival | | 60 | | whole membrane | | 70 | 30 | | 0 | 130 | | 0 | | 0 | 0 | | | 0 | 0 | | 0 | | 0 | | 0 | | 0 | |  |  |  |  |  |  |
| C17 | 100 | adjacent normal liver | | survival | | 60 | | whole membrane | | 0 | 90 | | 10 | 210 | | 30 | | 0 | 0 | | | 30 | 0 | | 0 | | 0 | | 0 | | 30 | |  | | |  |  |  |
| D17 | 101 | hepatocellular carcinoma | | survival | | 61 | | whole membrane | | 100 | 0 | | 0 | 100 | | 0 | | 0 | 0 | | | 0 | 0 | | 0 | | 0 | | 0 | | 0 | | 0 | | |  |  |  |
| E17 | 101 | hepatocellular carcinoma | | survival | | 61 | | whole membrane | | 100 | 0 | | 0 | 100 | | 0 | | 0 | 0 | | | 0 | 0 | | 0 | | 0 | | 0 | | 0 | |  |  |  |  |  |  |
| F17 | 101 | adjacent normal liver | | survival | | 61 | | whole membrane | | 60 | 40 | | 0 | 140 | | 10 | | 0 | 0 | | | 10 | 0 | | 0 | | 0 | | 0 | | 10 | |  | | |  |  |  |
| G17 | 102 | hepatocellular carcinoma | | death | | 9 | | whole membrane | | 80 | 20 | | 0 | 120 | | 80 | | 20 | 0 | | | 120 | 50 | | 20 | | 0 | | 90 | | 210 | | 235 | | |  |  |  |
| H17 | 102 | hepatocellular carcinoma | | death | | 9 | | whole membrane | | 70 | 30 | | 0 | 130 | | 70 | | 30 | 0 | | | 130 | 60 | | 20 | | 10 | | 130 | | 260 | |  |  |  |  |  |  |
| I17 | 102 | adjacent normal liver | | death | | 9 | | whole membrane | | 20 | 80 | | 0 | 180 | | 20 | | 0 | 0 | | | 20 | 0 | | 0 | | 0 | | 0 | | 20 | |  | | |  |  |  |
